# Electrochemical upcycling of spent ITO via charge-induced deconstruction

**DOI:** 10.1038/s41467-026-75921-w

**Published:** 2026-07-28

**Authors:** Rongcen Zhao, Xiaotao Lv, Zepeng Lv, Shaolong Li, Yong Fan, Jilin He, Jianxun Song

**Affiliations:** 1 Zhongyuan Critical Metals Laboratory, Zhengzhou University, Zhengzhou, Henan China; 2 National Key Laboratory of Special Rare Metal Materials, Zhengzhou University, Zhengzhou, Henan China; 3 State Key Laboratory of Critical Metals Beneficiation, Metallurgy and Purification, Zhengzhou University, Zhengzhou, Henan PR China; 4 Wuhan University of Science and Technology, Wuhan, China

**Keywords:** Electrochemistry, Metals and alloys, Theory and computation

## Abstract

Spent indium tin oxide (s-ITO, the mass ratio of In_2_O_3_ to SnO_2_ is approximately 9:1) represents the main secondary resource for the rare metal indium, possessing exceptionally potential for reutilization. However, traditional hydrometallurgical or pyrometallurgical recycling methods grapple with substantial challenges, including low product purity, lengthy processes, and environmental pollution. This paper presents an electrochemical upcycling strategy for spent ITO based on charge-induced deconstruction, effectively addressing the aforementioned issues. In this approach, spent ITO serves as the anode in an aqueous electrolysis system. The number of oxygen vacancies on the ITO surface increases drastically under charge excitation, leading to weakened metal-oxygen bonds and the subsequent release of indium and tin ions into the electrolyte. These ions then migrate to the cathode, where high-purity indium tin alloy is obtained by leveraging the differences in electrochemical properties between impurity ions and the target metal ions. Notably, the electrolyte employed in this process can be reused, which significantly reduces wastewater generation. Through systematic optimization of electrolysis parameters, a cathode current efficiency of 71.12% and an energy consumption of 4.87 kWh kg^−1^ are achieved in a hundred-ampere scale trial, fully demonstrating the industrial applicability of this method.

## Abstract (web summary)

An electrochemical upcycling strategy for spent indium-tin oxide (ITO) via charge-induced deconstruction. Spent ITO acts directly as the anode, where charge excitation creates oxygen vacancies to release indium and tin ions, deposited as a high-purity In-Sn alloy.

## Introduction

Indium (In) is extensively utilized in the electronic device and semiconductor industries owing to its superior optoelectronic properties and robust chemical stability^1^. Over 70% of global In consumption is attributed to the production of indium tin oxide (ITO, the mass ratio of In_2_O_3_ to SnO_2_ is approximately 9:1), an indispensable thin-film material for conductive glass in liquid crystal displays^2–5^. Nevertheless, the utilization efficiency of ITO during target fabrication, processing and sputtering deposition is below 30%, generating massive amounts of spent ITO (s-ITO)^6^. As a crucial secondary resource of In, s-ITO possesses considerable recycling value. Consequently, the recovery of in from s-ITO exhibit great market potential, which has aroused widespread concern in both academia and industry.

S-ITO is mainly recycled through traditional pyrometallurgical and hydrometallurgical processes, both of which have distinct industrial merits. Pyrometallurgical recovery is characterized by strong industrial adaptability, excellent process robustness, and high scalability, rendering it suitable for large-scale s-ITO feedstock processing; hydrometallurgical recovery possesses relatively high metal selectivity and a well-established engineering application system^7–9^. Despite these advantages, both traditional processes have inherent limitations. Pyrometallurgy, which typically employs carbon-based materials as reducing agents, is plagued by massive CO_2_ emissions and high energy consumption^10^. Hydrometallurgy involves the extensive use of acids, alkalis, and other reagents, generates substantial chemical wastewater, features complex processes and lengthy processing cycles, and faces severe environmental pressure from wastewater treatment^11,12^. In recent years, electrolysis methods, featuring short process flows and environmental benignity, have also been adopted for s-ITO recycling. Unlike the electrolysis process in hydrometallurgical recovery, which merely serves as a process for extracting metals via electrolysis after prolonged pre-leaching^6,13,14^, the electrolysis methods mentioned in this paper serve as the core process and enable the direct extraction of metals from s-ITO. A typical representative of such electrolysis methods is the molten salt electro-deoxidation technology^15^. Our group has conducted extensive research on the recycling of s‑ITO via electro‑deoxidation. The utilization of s-ITO as the cathode during electrolysis in CaCl_2_-based molten salt allows the direct production of In-Sn alloy on the cathode. However, the depolarization effect of the liquid alloy formed after electro‑deoxidation can lead to the underpotential deposition of metallic calcium on the cathode product. Furthermore, the employment of carbon anodes generates carbon oxide gases, and the anode materials increases the risk of elevated impurities in the product^16,17^. Preliminary studies in our group demonstrate that direct electrolysis with s‑ITO as the anode in aqueous solution results in pronounced electrochemical dissolution, facilitating the release of metal ions into the electrolyte^18^. Driven by this observation and the limitations of traditional recycling approaches, we herein propose an electrochemical recycling strategy based on the charge‑induced deconstruction of s‑ITO.

In this route, a H_2_SO_4_-based system is selected as the electrolyte, with a certain concentration of NaCl incorporated. S-ITO is directly utilized as anode, and In-Sn alloy is deposited on the titanium cathode plate. Under charge excitation, the reactivity of oxygen on the s-ITO anode surface is activated, thereby weakening the interaction between metal and oxygen ions. Indium and tin ions are released into the electrolyte, migrate to the cathode, and are reduced. The addition of NaCl to the electrolyte can further promote anodic dissolution and cathodic deposition.

## Results

### Verification of charge‑promoted anodic dissolution of s-ITO

**Fig. 1:**
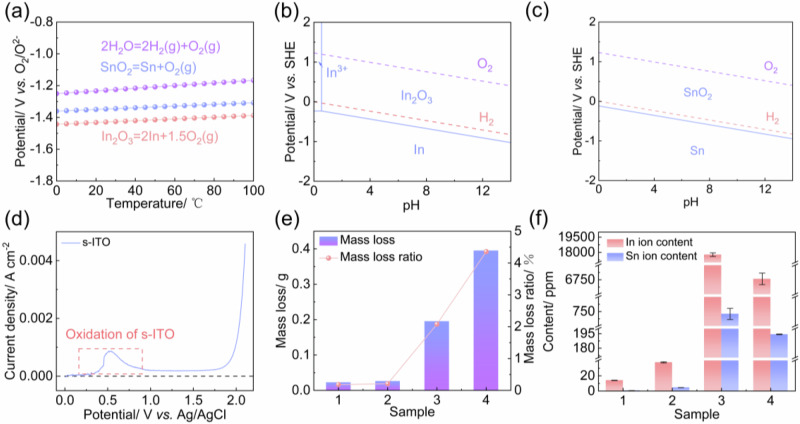
Feasibility investigation of charge‑promoted anodic dissolution of s-ITO. **a** Theoretical reduction potentials of In_2_O_3_, SnO_2_, and H_2_O (data from HSC Chemistry 6.0, The potential calculation method is detailed in Section 1 of Supplementary Information); Potential-pH (E-pH) diagrams of **b** In-H_2_O system and **c** Sn-H_2_O system (data from HSC Chemistry 6.0); **d** Linear sweep voltammetry (LSV) curve of s-ITO in H_2_SO_4_-NaCl solution (scan rate: 0.1 V s^−1^); In the electrolyte under different leaching/electrolysis conditions (To ensure consistency, Samples 1–4 were prepared from the same s-ITO source through wire-cutting and then treated under varying conditions. 1: 5.52 mol L^−1^ H_2_SO_4_, 12 h; 2: 5.52 mol L^−1^ H_2_SO_4_ + 1.0 mol L^−1^ NaCl, 12 h; 3: 5.52 mol L^−1^ H_2_SO_4_, 0.072 A cm^−2^, 1.5 h; 4: 5.52 mol L^−1^ H_2_SO_4_ + 1.0 mol L^−1^ NaCl, 0.072 A cm^−2^, 1.5 h), **e** the changes in mass loss of s-ITO anode (The mass loss ratio is obtained by the following formula, ${{{{\rm{\eta }}}}}_{1}=\frac{{m}_{b}-{m}_{a}}{{m}_{b}}$ Where ${\eta }_{1}$ is the mass loss ratio, %; m_a_ is the mass of s-ITO after treatment; m_b_ is the mass of s-ITO before processing.), **f** In and Sn ions concentrations in electrolytes. Data for (**f**) is presented as mean values ± standard deviation (SD) (*n* = 3).

According to the results of thermodynamic calculations (Fig. 1a), the theoretical reduction potentials (*vs*. O_2_/O^2−^) of the metal ions in In_2_O_3_ and SnO_2_ are slightly more negative than the hydrogen evolution reaction (HER) potential (*vs*. O_2_/O^2−^) in water^19,20^. In practice, hydrogen evolution usually requires a substantial potential more negative than −1.5 V (*vs*. O_2_/O^2−^) due to the high overpotential required for HER. This fact lays the groundwork for the electrolysis recovery of s-ITO in an aqueous solution. As depicted in the E-pH diagrams (Fig. 1b, c), with decreasing pH, metallic indium and tin exhibit expanded thermodynamic stability domains relative to their respective oxide phases. This creates a thermodynamically favorable region where metallic indium and tin become the predominant stable phases. At relatively more positive potentials, metallic indium and tin can be obtained. Linear sweep voltammetry (LSV) curves (Fig. 1d and Supplementary Fig. 1) also demonstrate that when s-ITO is employed as the working electrode, an obvious current response appears prior to electrolyte decomposition with the potential scanned positively (see the dashed box region in Fig. 1d). When glassy carbon is used as the working electrode, no current response on LSV curve is observed prior to electrolyte decomposition. It can be concluded from the comparison of LSV curves that the current response corresponds to the electrochemical dissolution of s-ITO, including the oxidation of oxygen ions and the release of indium and tin ions. Figure 1e illustrates the dissolution of s‑ITO under various conditions (Calculation reference Supplementary Information). After acid leaching in 5.52 mol L^−1^ H_2_SO_4_ aqueous solution for 12 h, the mass loss of the s‑ITO sample is 0.022 g, corresponding to a mass loss ratio of 0.18%. Upon introduction of 1.0 mol L^−1^ NaCl, the mass loss (0.026 g) increases only slightly, with a negligible enhancing effect. Upon applying current in the above electrolytes, pronounced mass loss of the s‑ITO anode is observed after only 1.5 h of electrolysis: 0.195 g (mass loss ratio of 2.07%) in H_2_SO_4_ solution and 0.396 g (mass loss ratio of 4.35%) in H_2_SO_4_‑NaCl solution, respectively. These results clearly demonstrate that current can effectively promote the dissolution of s‑ITO, while the introduction of NaCl further enhances this process. In the absence of an applied current, the effect of Cl^−^ on promoting the dissolution of s-ITO is limited (Fig. 1e). Upon the application of current, Cl^−^ on the anode surface may be oxidized to Cl_2_, which can further react with water to generate HCl. Therefore, the potential effects of other chlorine species on the dissolution of s-ITO were also investigated. Specifically, Cl_2_ was introduced into the H_2_SO_4_-NaCl electrolyte (the preparation method of Cl_2_ is detailed in Supplementary Fig. 2a), and the Cl_2_ gas escaped from the edge of the s-ITO electrode. S-ITO experienced a mass loss of only 0.018 g after a continuous introduction period. Additionally, an acid leaching experiment of s-ITO was conducted in the H_2_SO_4_-HCl solution, and s-ITO experienced a mass loss of only 0.013 g (Supplementary Fig. 2b). These results indicate that in the absence of current, these chlorine species (Cl_2_ and HCl) are hardly capable of independently accelerating the dissolution of s-ITO. The Tafel curves (Supplementary Fig. 3) further reveal that the incorporation of NaCl into the H_2_SO_4_ electrolyte induces a negative shift in the corrosion potential of s-ITO, which confirms that the promotional effect of Cl^-^ on s-ITO dissolution can be effectively exerted under the action of charge. Subsequently, the concentrations of indium and tin ions in the solution were investigated under the same experimental conditions as those in Fig. 1e, with the results presented in Fig. 1f. For traditional acid leaching alone, the accumulation of indium and tin ions in the solution remained extremely limited after 12 h, with both concentrations below 50 ppm. In contrast, upon the application of current for 1.5 h, the concentrations of indium and tin ions in the electrolyte increased drastically. In the H_2_SO_4_ solution, the concentrations of indium and tin ions reached 17774.7 and 742.0 ppm, respectively; while in the H_2_SO_4_-NaCl solution, the concentrations were 6765.4 ppm and 194.3 ppm, respectively. These results confirm that the introduction of current significantly accelerates the anodic dissolution of s-ITO compared with the acid leaching alone. The decrease of ion concentrations upon adding NaCl to the H_2_SO_4_ solution after electrolysis is attributed to the reduction of anodically dissolved metal ions at the cathode, which will be discussed in subsequent section on cathodic deposition.

### Anodic dissolution mechanism of s-ITO

**Fig. 2:**
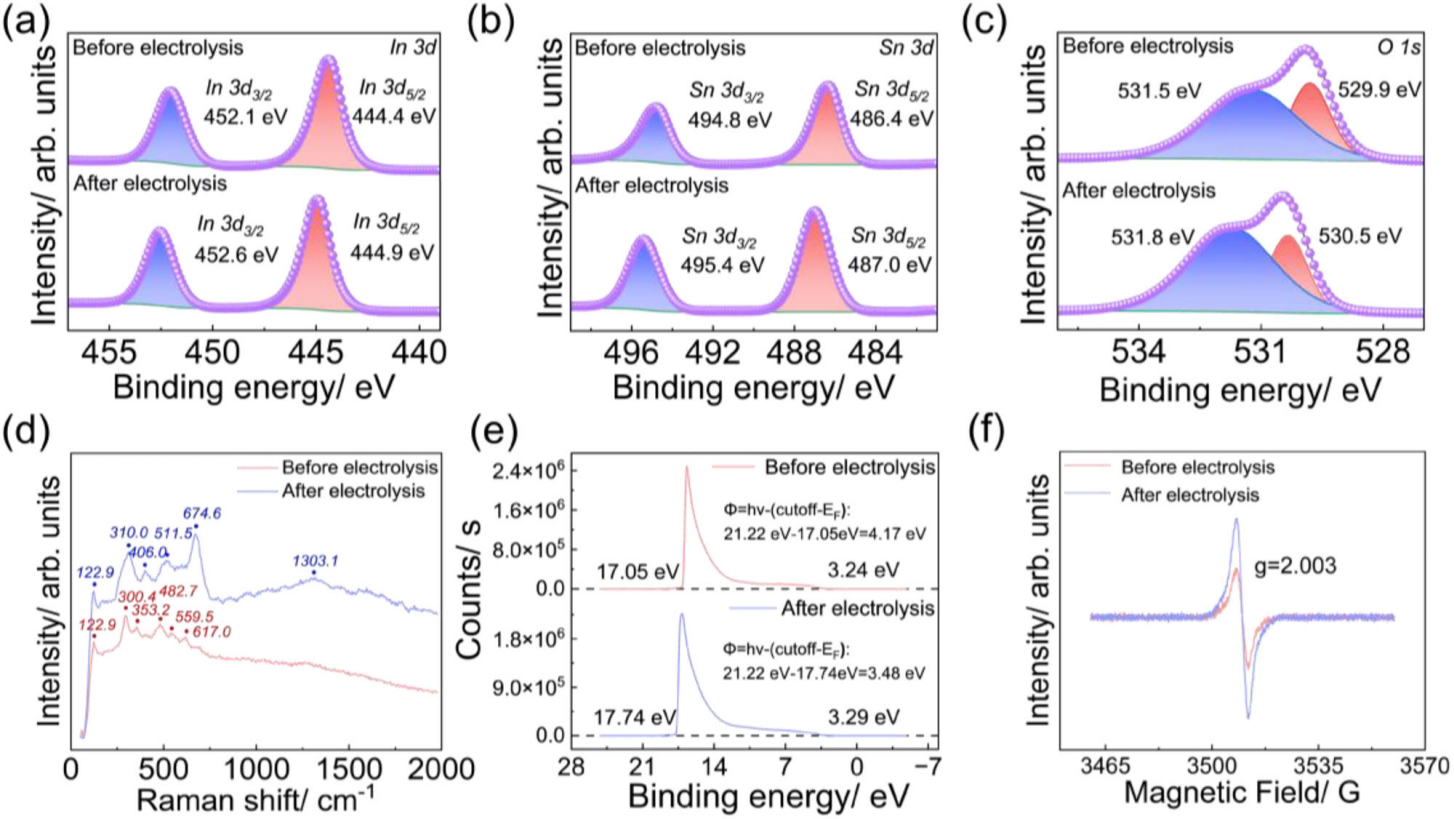
Research on anodic deconstruction mechanism. X-ray photoelectron spectroscopy (XPS) spectra of s-ITO before and after electrolysis: **a** *In 3d*, **b** *Sn 3d*, **c** *O 1s*; Raman spectra (**d**), Ultraviolet photoelectron spectroscopy (UPS) spectra (**e**) and Electron paramagnetic resonance (EPR) spectra (**f**) of s-ITO before and after electrolysis.

In order to elucidate the effect of charge on the dissolution process of s-ITO, detailed analysis of In, Sn, and O in s-ITO was conducted using X-ray photoelectron spectroscopy (XPS). Examination of the *In 3d* spectrum (Fig. 2a) unveiled subtle changes, characterized by a notable high-energy shift in the positions of the *In 3d*_*3/2*_ and *In 3d*_*5/2*_ peaks, accompanied by an increase in binding energy of 0.5 eV. Similarly, in the *Sn 3d* spectrum (Fig. 2b), the *Sn 3d*_*3/2*_ and *3d*_*5/2*_ peaks exhibited the same movement trend as the *In 3d* peak, with each peak increasing by 0.6 eV. This phenomenon is primarily attributed to the metal ions in s-ITO attracting Cl^−^ with higher electronegativity after passing the current^21^, necessitating the metal ions to overcome greater attractive forces. Despite the changes in the binding energy of indium and tin ions, the peak intensity of these metal ions remained consistent before and after electrolysis. In stark contrast, the intensity of the *O 1s* peak at 529.9 eV^22^ in ITO exhibited a marked decline following electrolysis (Fig. 2c). This result indicates a decrease in the lattice oxygen content, which suggests an increase in the number of oxygen defects in s-ITO induced by the electrolysis process^23,24^. Simultaneously, after the electrolysis process, both the lattice oxygen peak at 529.9 eV and the adsorbed oxygen peak at 531.5 eV^25^ underwent a high-energy direction shift, by 0.6 and 0.3 eV, respectively. This is attributed to the adsorption of highly electronegative ions such as oxygen and chlorine on the anode surface, which leads to changes in electron cloud density^26^. To eliminate the potential errors arising from the surface sensitivity of XPS measurements, depth-profiling XPS measurements were further performed on the post-electrolysis samples, and the results are shown in Supplementary Fig. 4. A spot with a smaller size was adopted for the tests. Compared with the unreacted central region in the depth profile, the near-surface reaction region exhibited significant changes, which were consistent with the results illustrated in Fig. 2a–c. This further confirms the preferential interaction between charges and oxygen in s-ITO.

Prior to electrolysis, the Raman spectra of s-ITO (Fig. 2d) exhibited characteristic vibration modes at *122.9*, *300.4*, *353.2*, *482.7*, *559.5*, and *617.0 *cm^−1^, which were consistent with the expected peaks of the In_2_O_3_ crystal structure^27,28^. Subtle shifts of these characteristic peaks were observed, attributed to Sn dopant lattice incorporation that introduced Sn-O vibrational contributions interacting with In-O modes^29,30^. After electrolysis, significant transformations occurred in the surface spectral features of s-ITO: the In-O vibration mode corresponding to the InO_6_ structural unit at *122.9 *cm^−1^ remained unchanged^31^, while new distinct peaks emerged at *283.0*, *406.0*, *511.5*, and *674.6 *cm^−1^. The post-electrolysis characteristic peaks showed a pronounced blue shift, which was ascribed to lattice distortion induced by oxygen vacancy defects generated via charge transfer processes^32,33^; this distortion modified metal-oxygen vibrational modes, evidenced by slight peak asymmetry in the post- electrolysis spectra^34^. Notably, a broad peak at *1303.1 *cm^−1^ emerged in the post-electrolysis spectrum, consistent with previously reported oxygen-related Raman features^35–37^. This peak, corresponding to O–O interactions, exhibited significantly increased intensity post-electrolysis due to enhanced oxygen vacancy concentration on the substrate surface. Further analysis of Raman half-width variations revealed substantial broadening of characteristic peaks after electrolytic treatment, further confirming oxygen defect generation on the s-ITO surface during the anodic process^38–40^.

As shown in Fig. 2e, the work function of s-ITO decreased from 4.17 to 3.48 eV after electrolysis, indicating weakened electron attraction of s-ITO and easier electron escape^41^. This is attributed to structural changes caused by the increased number of anodic defects^42^, and oxygen defects promote the accumulation of surrounding electrons in these vacancies^43,44^. Electron paramagnetic resonance (EPR) spectroscopy was employed to analyze the changes in the number of defects in s-ITO before and after electrolysis. As shown in Fig. 2f, s-ITO exhibits an obvious signal at *g* = 2.003, indicating the presence of oxygen vacancy defects in the material. After electrolysis, the signal intensity at the same position increases significantly, demonstrating that the defect concentration in the s-ITO anode is elevated^45^. In summary, under the action of electric charge, oxygen in s-ITO is more likely to escape after gaining energy, leading to the generation of oxygen vacancy defects.

**Fig. 3:**
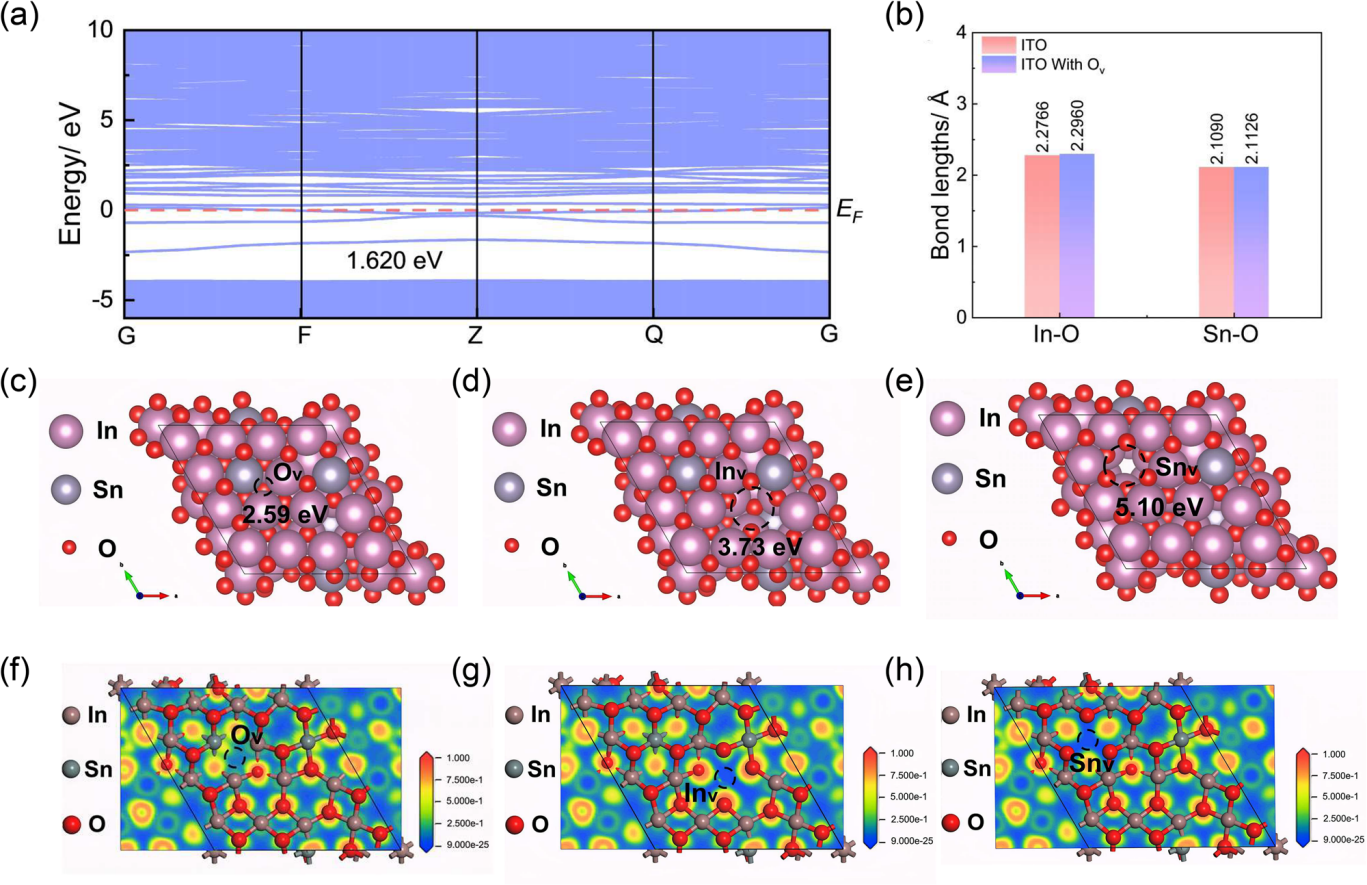
Research on the influence mechanism of oxygen vacancies on the crystal structure and electronic structure of ITO. **a** Band structure diagram of ITO (SnO_2_ doped In_2_O_3_: 9.375 at.%, after removing O_50_); **b** The effect of oxygen vacancies on atomic bond length in ITO; ITO (2 2 2) vacancy model and formation energy: **c** O_v_ (after removing O_50_), **d** In_v_ (after removing In_15_), **e** Sn_v_ (after removing Sn_22_); Electron localization function (ELF; The background color describes the degree of spatial localization of electrons, ranging from 0 to 1. The closer it is to 1, the redder the color, and the higher the degree of electron localization; The closer it is to 0, the bluer the color, and the lower the electron localization): **f** O_v_ (after removing O_50_), **g** In_v_ (after removing In_15_), **h** Sn_v_ (after removing Sn_22_).

First-principles calculations were performed to elucidate the intrinsic mechanism by which oxygen vacancies promote the anodic deconstruction of s-ITO (The lattice optimization of the model (Supplementary Fig. 5) and partial energy calculations (Supplementary Fig. 6) are provided in Section 2 of Supplementary Information). Combined with the X-ray diffraction (XRD) pattern of s-ITO (Supplementary Fig. 7a) and previous studies^46^, the (2 2 2) crystal plane of ITO exhibits the strongest diffraction peak and the lowest surface energy, indicating that the (2 2 2) plane is the most stable facet of ITO. Therefore, the (2 2 2) crystal plane was selected for cleavage, and the model was maintained at 5 atomic layers. The projection diagram of the ITO (2 2 2) crystal plane model in the Z direction is shown in Supplementary Fig. 7b, where different atoms are labeled with serial numbers for easy distinction. An oxygen vacancy was constructed on the ITO surface by removing O_50_, and the *E*_*g*_ of ITO further decreased to 1.620 eV (Fig. 3a), with enhanced electrical conductivity. This indicates that the introduction of oxygen vacancies brings additional electrons, increasing the number of carriers and promoting electron transfer during the reaction process^47^. As shown in Fig. 3b, the formation of oxygen vacancies causes the In–O bond length around the vacancies to increase from 2.277 to 2.296 Å, while the Sn–O bond length increases from 2.109 to 2.113 Å. The change in bond length reflects the lattice distortion induced by oxygen vacancies; the elongation of the bonds implies weakened bonding force between the metal and oxygen, which facilitates oxygen escape. Released from the constraint of oxygen, In and Sn are released into the electrolyte as ionic species.

The formation energy of oxygen vacancies (O_v_) is determined to be 2.59 eV (Fig. 3c), while that of indium vacancies (In_v_) and tin vacancies (Sn_v_) is 3.73 eV (Figs. 3d) and 5.10 eV (Fig. 3e), respectively. Since the vacancy formation process is endothermic, all calculated formation energies are positive. Notably, the formation energy of O_v_ is lower than that of In_v_, with Sn_v_ exhibiting the highest formation energy among the three types of vacancies. This observation demonstrates that oxygen vacancies exhibit the highest formation tendency, whereas tin vacancies are the most challenging to generate.

As a dimensionless scalar field, the electron localization function (ELF) describes the degree of electron localization in space within the range of 0 to 1. The redder the color, the closer it is to 1. This represents highly localized electrons. The bluer the color, the closer it is to 0, indicating extremely low electronic positioning. ELF of the oxygen vacancy formed by removing O_50_ is shown in Fig. 3f. A blue-green region appears at the center of the oxygen vacancy, which is caused by the two electrons released after the removal of oxygen being shared by the adjacent indium and tin ions. The electron localization density of the adjacent indium and tin ions increases, especially that of the adjacent tin ions, which increases significantly. The electrons generated by the oxygen vacancy are mainly shared by the adjacent tin ions, reflecting an increase in carriers. The ELF of the ITO (2 2 2) crystal plane with indium vacancies is shown in Fig. 3g. A blue-green region appears at the center of the indium vacancy; since the three electrons generated by the indium vacancy are shared by the adjacent oxygen ions, the electron clouds of the adjacent oxygen ions are strongly attracted, leading to a significant expansion of the electron localization density of the adjacent oxygen ions. As shown in Fig. 3h, the orange-yellow regions around the oxygen ions adjacent to the tin vacancy are significantly expanded, indicating a significant enhancement of the electron localization density of the oxygen ions. This is caused by the fact that the effective charge of tin ions is higher than that of indium ions, resulting in a stronger polarization effect on the electron cloud of oxygen ions. In summary, oxygen is the easiest to dissolve and form oxygen vacancies. The ionic radius of oxygen (1.71 Å) is significantly smaller than that of indium (2.46 Å) and tin (2.48 Å)^48^; therefore, the lattice distortion caused by the formation of oxygen vacancies is smaller, and the surrounding lattice relaxation energy is larger. Meanwhile, the charge difference of 2 electrons introduced after oxygen dissolution is significantly smaller than that caused by indium and tin dissolution, requiring less compensation energy; thus, oxygen vacancies are easier to form.

### Existential forms and electrochemical behaviors of indium ions and tin ions

**Fig. 4:**
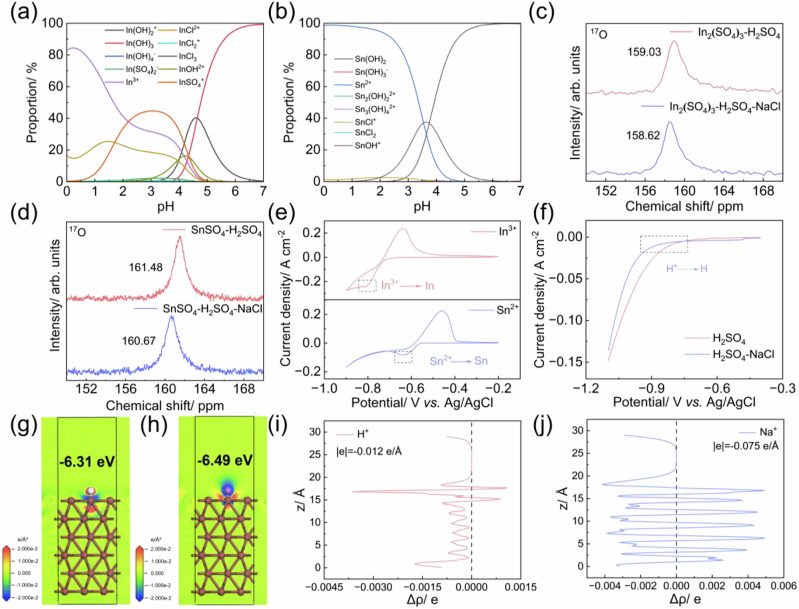
Research on the existential forms and electrochemical behaviors of In ions and Sn ions. The ion distribution maps of In^3+^ (**a**) and Sn^2+^ (**b**) in NaCl-H_2_SO_4_ solution; ^17^O nuclear magnetic resonance (NMR) spectra of solutions before and after adding NaCl, **c** In_2_(SO_4_)_3_-H_2_SO_4_ solution, **d** SnSO_4_-H_2_SO_4_ solution; **e** Cyclic voltammetry (CV) curves of indium ions and tin ions (H_2_SO_4_ concentration: 5.52 mol L^−1^, NaCl concentration: 1.2 mol L^−1^, indium ions concentration: 0.01 mol L^−1^, tin ions concentration: 0.01 mol L^−1^, scan rate: 0.02 V s^−1^); **f** Linear sweep voltammetry (LSV) curves obtained in H_2_SO_4_ and H_2_SO_4_-NaCl solutions (H_2_SO_4_ concentration: 5.52 mol L^−1^, NaCl concentration: 1.2 mol L^−1^, scan rate: 0.1 V s^−1^); Differential charge density sectional projection: **g** H^+^ adsorbed on the Ti cathode, **h** Na^+^ adsorbed on the Ti cathode; Spatial distribution of cumulative differential charge density projected along the *z*-axis: **i** H^+^ adsorbed on the Ti cathode, **j** Na^+^ adsorbed on the Ti cathode.

In the H_2_SO_4_ solution, as the pH decreases, In^3+^ becomes the main existing form, and the content of InSO_4_^+^ complex ion decreases (Supplementary Fig. 8a). Similarly, in the low-pH environment, Sn^2+^ tends to exist independently without complexing with SO_4_^2−^ (Supplementary Fig. 8b). However, with the addition of NaCl, the content of InCl^2+^ increases significantly in the low-pH environment, indicating that In^3+^ has a stronger affinity for Cl^−^ (Fig. 4a). The generation of SnCl^+^ indicates that the binding tendency of Sn^2+^ with Cl^−^ is much greater than that with SO_4_^2−^ (Fig. 4b). The results demonstrate that Cl^−^ exhibits a strong binding affinity toward In^3+^ and Sn^2+^, leading to the formation of chloro-coordinated complex ions. The nuclear magnetic resonance (NMR) spectroscopic results reveal that the addition of NaCl to H_2_SO_4_ solution leads to a pronounced negative shift of the ^17^O signal, which is ascribed to the decreased electron density around the oxygen atoms, indicating that NaCl weakens the interaction between SO_4_^2−^ and In^3+^^49^ (Fig. 4c). Notably, the introduction of Cl^-^ also gives rise to a reduction in the chemical shifts of Sn^2+^ and SO_4_^2−^ (Fig. 4d), which is attributed to the strong binding affinity of Cl^−^ with Sn^2+^ ^50^.

In the H_2_SO_4_ solution containing tin ions, a prominent current signal was observed at −0.61 V (*vs*. Ag/AgCl) on the cyclic voltammetry (CV) curve (Supplementary Fig. 9a), which indicates the occurrence of the reduction reaction of tin ions. In contrast, no distinguishable reduction peak could be identified on the CV curve recorded in the H_2_SO_4_ solution with indium ions; however, a reduction signal emerged at −0.72 V (*vs*. Ag/AgCl) on the square wave voltammetry (SWV) curve with higher detection sensitivity (Supplementary Fig. 9b), suggesting that indium ion is reducible in the H_2_SO_4_ solution. Nevertheless, upon the addition of NaCl to the H_2_SO_4_ solution, the reduction signal of tin ions on the CV curve was significantly enhanced (Fig. 4e). Likewise, a well-defined reduction peak corresponding to indium ions was observed on the CV curve. Accordingly, it can be deduced that the introduction of NaCl promoted the reduction of these ions at the cathode, with a more pronounced effect exerted on indium ions. An in-depth exploration of the reduction behaviors of indium ions and tin ions were conducted, with key findings derived from the corresponding electrochemical curves (Supplementary Figs. 10 and 11). For indium ions, with the increase of scan rate, the peak current on the CV curve is enhanced, accompanied by a negative shift of the reduction potential. Linear fitting analyses reveal a direct proportional relationship between the peak current density and the square root of the scan rate, while a linear correlation is also observed between the reduction peak potential and the logarithm of the scan rate. These findings collectively demonstrate that the reduction of indium ions is a diffusion-controlled quasi-reversible process. Similarly, tin ions exhibit analogous electrochemical reduction behaviors at the cathode, confirming that the reduction of tin ions is also governed by diffusion and behaves as a quasi-reversible process.

LSV was employed to investigate the effect of H_2_SO_4_ concentration on HER. The increase in H_2_SO_4_ concentration leads to a continuous positive shift of the hydrogen evolution potential (Supplementary Fig. 12), indicating that the H^+^ introduced by H_2_SO_4_ promotes the occurrence of HER. After the addition of NaCl, the H_2_SO_4_-NaCl solution exhibited a more negative hydrogen evolution potential (Fig. 4f), indicating that the addition of NaCl effectively inhibited HER. As shown in Fig. 4g, the adsorption energy of H^+^ on the Ti cathode surface is −6.31 eV. The blue region indicates electron gain, and the red region indicates electron loss^51^. In the differential charge density diagram of the vertical section, an obvious red electron cloud appears around H^+^, and an obvious blue electron cloud is generated at the adsorption site, indicating that electrons are transferred from H⁺ to the electrode surface. In contrast, the adsorption energy of Na^+^ is −6.49 eV (Fig. 4h), indicating that Na^+^ is more likely to be adsorbed on the Ti cathode than H^+^. An obvious blue electron cloud appears around Na^+^, and the adsorption site presents a red electron cloud, indicating that electrons on the electrode surface are transferred to Na^+^. Figure 4i shows that the average differential charge density of H^+^ in the *Z* direction is −0.012 e Å^−1^, indicating that H^+^ adsorption leads to electron enrichment on the surface^52^. The results in Fig. 4j show that the average differential charge density of Na^+^ is −0.075 e Å^−1^, and Na^+^ adsorption leads to more electron accumulation^53^. The electron distribution on the electrode surface has an obvious impact on the attraction and reduction process of metal ions. Compared with H^+^ adsorption, Na^+^ adsorption leads to an increase in the electron content on the electrode surface, and a significant change in the surface charge density, forming a localized electron-rich structure^54^, which results in a stronger interaction between the It cathode and indium/tin ions^55,56^, thereby promoting the adsorption and reduction processes of indium ions and tin ions on the cathode surface.

**Fig. 5:**
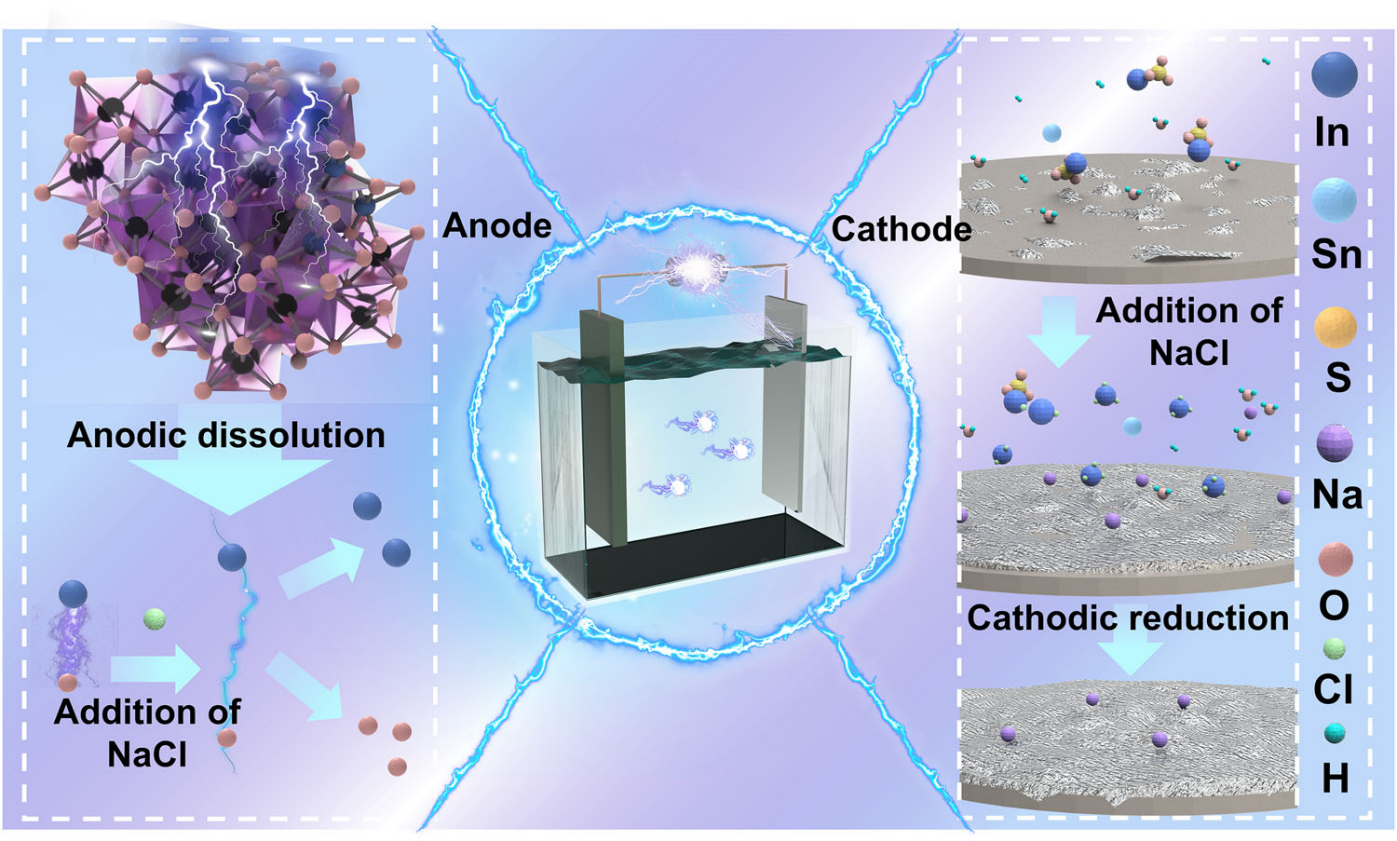
Schematic illustration of the electrochemical reaction mechanism of s-ITO. It depicts charge-induced deconstruction of s-ITO accompanied by metal ion migration into electrolyte, as well as the promotional effect of NaCl on both anodic dissolution and cathodic deposition.

Based on the above results, the reaction mechanism of s-ITO during electrolysis is summarized in Fig. 5. The s-ITO anode undergoes charge activation, which weakens the bonding strength between metal cations and oxygen ions. The preferential escape of oxygen from s-ITO induces the formation of oxygen vacancy defects. These oxygen vacancies trigger lattice distortion and carrier accumulation in the anode, further weakening ionic interactions and facilitating anodic ion leaching. The oxygen deficiency in s-ITO reduces the bonding between indium, tin and oxygen. Consequently, indium and tin dissolve into the electrolyte in ionic form and subsequently migrate to the cathode for reduction. The introduction of NaCl negatively shifts the corrosion potential of s-ITO, thereby accelerating anodic dissolution. Furthermore, the strong adsorption of Na⁺ on the cathode suppresses H⁺ adsorption and inhibits the HER. Meanwhile, Na⁺ adsorption induces substantial electron accumulation at the cathode, strengthens the interaction between indium ions, tin ions and the cathode surface, and promotes the reduction of indium ions and tin ions.

### Optimization of electrolysis parameters

Electrolysis recovery was carried out using s-ITO as the anode and a Ti plate as the cathode (Supplementary Fig. 13). After electrolysis in H_2_SO_4_ solution, a thin layer of silvery-white metal was deposited on the cathode, and XRD analysis confirmed that the product was In-Sn alloy (Supplementary Fig. 14a). With the addition of NaCl, the cathode product exhibited a porous and sponge-like morphology with a significantly increased mass. XRD results verified that the product was still In-Sn alloy (Supplementary Fig. 14b). These findings not only underscore the feasibility of s‑ITO recovery via this method, but also confirm the beneficial role of NaCl in the electrolysis recovery of s‑ITO. This accounts for the lower measured concentrations of indium and tin ions in the electrolyte after electrolysis with NaCl addition compared to those without NaCl (Fig. 1f), which is attributed to the accelerated cathodic reduction of ions induced by NaCl. The effects of H_2_SO_4_ concentration, NaCl concentration, current density, and indium ions concentration on the current efficiency (the calculation method of current efficiency is provided in Section 3 of Supplementary Information) were further investigated.

**Fig. 6:**
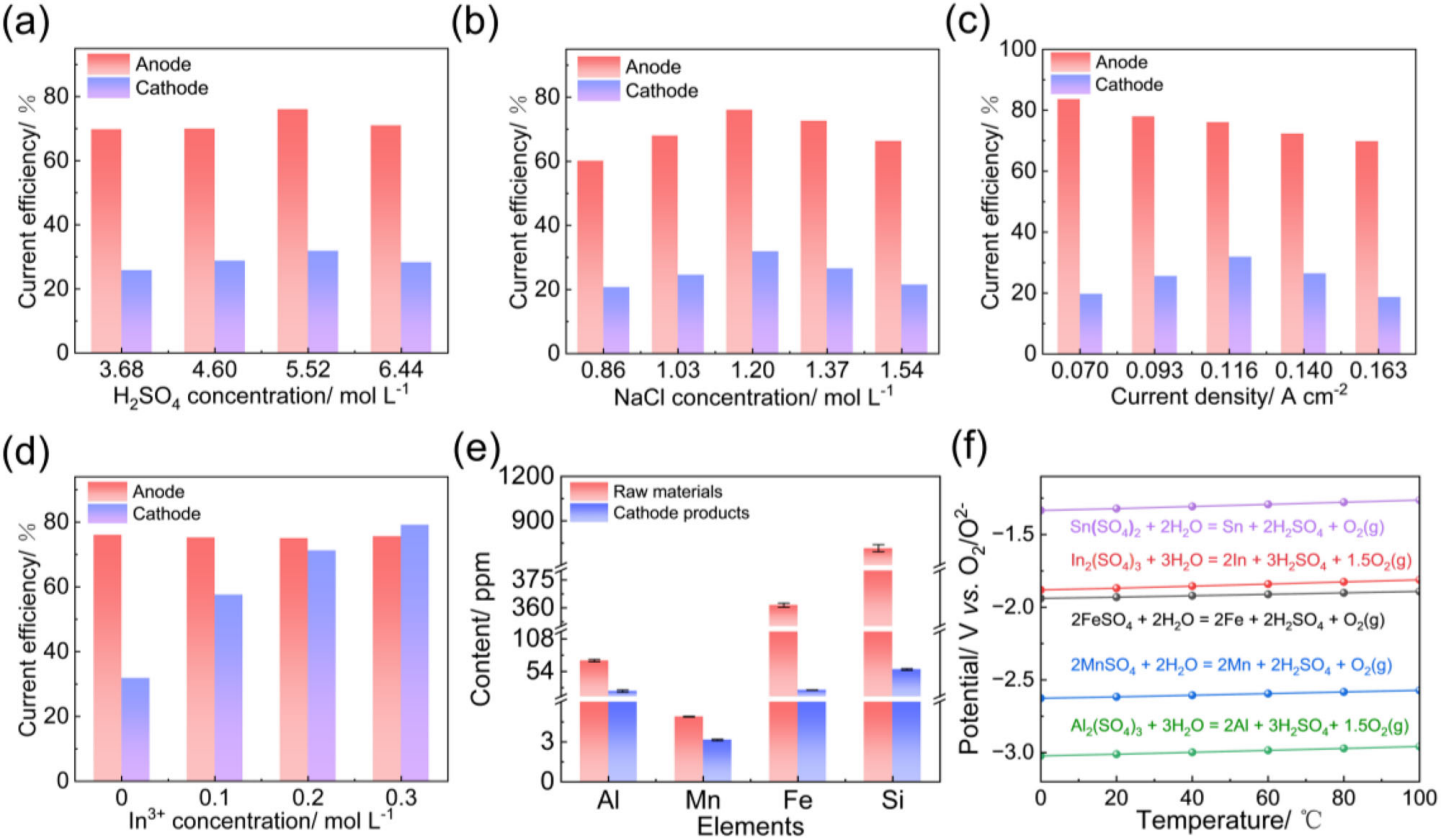
Research on optimization of process parameters. Anodic and cathodic and current efficiencies under different H_2_SO_4_ concentrations (**a**), NaCl concentrations (**b**), current densities (**c**), and initial indium ion concentrations (**d**), respectively. **e** Content of main impurities of raw material (s-ITO) and cathode product (H_2_SO_4_ concentration: 5.52 mol L^−1^; NaCl concentration: 1.2 mol L^−1^; current density: 0.116 A cm^−2^); **f** Theoretical reduction potentials of sulfates (data from HSC Chemistry 6.0, The potential calculation method is detailed in Section 1 of Supplementary Information). Data for (**e**) is presented as mean values ± standard deviation (SD) (*n* = 3).

The effects of H_2_SO_4_ on the current efficiencies of the cathode and anode are presented in Fig. 6a. With increasing H_2_SO_4_ concentration, the current efficiencies of both the anode and cathode first increase and then decrease. At a H_2_SO_4_ concentration of 5.52 mol L^−1^, the anodic current efficiency reaches a peak value of 76.39%, and the cathodic current efficiency achieves a maximum value of 31.96%. The above phenomenon is attributed to the fact that the increase of H_2_SO_4_ concentration promotes the anodic dissolution of s-ITO, leading to more indium and tin ions entering the electrolyte. However, an excessively high H_2_SO_4_ concentration may intensify the HER, thereby reducing the current efficiency. Figure 6b depicts the relationship between current efficiency and NaCl concentration. With increasing NaCl concentration, both the anodic and cathodic current efficiencies exhibit a trend of first increasing and then decreasing. The optimal anodic and cathodic current efficiencies are achieved at a NaCl concentration of 1.2 mol L^−1^. NaCl induces a negative shift in the corrosion potential of s-ITO, thus facilitating anodic dissolution. Nevertheless, an excessively high NaCl level increases the electrolyte viscosity^57^, which hinders ion migration and consequently lowers the current efficiency. The effect of current density on current efficiency was investigated (Fig. 6c). The anodic current efficiency shows a monotonically decreasing trend with increasing current density, which is attributed to the fact that a high current density induces more side reactions such as electrolyte decomposition. The cathodic current efficiency first increases and then decreases with increasing current density, and a relatively high cathodic current efficiency is achieved at a current density of 0.116 A cm^−2^. At low current densities, fewer metal ions dissolve into the electrolyte, and the cathodic discharge is dominated by the HER, resulting in low current efficiency. The cathodic current efficiency rises with increasing current density, yet an overly high current density also induces intense hydrogen evolution, thus lowering the current efficiency.

Evidently, during the initial stage of electrolysis, the insufficient concentration of indium ions in the electrolyte triggers severe hydrogen evolution at the cathode, leading to significant current loss and consequently low cathodic current efficiency. To monitor the variations in the concentration of metal ions during the electrolysis process, samples were collected from different regions of the electrolyte at various electrolysis stages for the determination of indium and tin ion concentrations (Supplementary Fig. 15). Prior to the electrolysis process, no indium and tin ions were present in the electrolyte. After 20 min of electrolysis, the concentrations of indium and tin ions increased significantly, with the highest concentration appearing at the anode side. As electrolysis continued, the metal ion concentrations increased further. At 80 min, the metal ion concentration at the anode became nearly stable, while the concentration at the cathode was comparable to that at the anode. Therefore, to achieve a synergy between the anodic and cathodic current efficiencies, additional indium ions should be introduced into the electrolyte to compensate for the current loss caused by insufficient ion concentration at the initial stage. Indium ions were introduced into the electrolyte in the form of In_2_(SO_4_)_3_. As shown in Fig. 6d, the anodic current efficiency did not change significantly with increasing indium ion concentration, indicating that the addition of indium ions does not affect anodic dissolution. In contrast, the cathodic current efficiency was markedly improved. Specifically, when the indium ion concentration was increased to 0.3 mol L^−1^, the cathodic current efficiency (79.11%) exceeded the anodic current efficiency (75.44%). This suggests that part of the indium in the cathodic product originates from the added In_2_(SO_4_)_3_ rather than from indium ions dissolved at the anode. When the concentration of In_2_(SO_4_)_3_ was 0.2 mol L^−1^, the cathodic current efficiency (71.36%) was slightly lower than the anodic current efficiency (74.97%), which is regarded as a favorable condition for a nearly balanced reaction rate at the anode and cathode.

The Scanning electron microscopy (SEM)-energy dispersive spectroscopy (EDS) results of the cathodic product are shown in Supplementary Fig. 16. The product is composed of particles with various sizes and different pore structures. Indium and tin are uniformly distributed in the In-Sn alloy. TEM results of the cathode product further demonstrate that it exhibits a polycrystalline structure with low crystallinity (Supplementary Fig. 17a) and uniform elemental distribution at the nanoscale (Supplementary Fig. 17b, c).

The impurity contents in the raw s-ITO and the In-Sn alloy are presented in Fig. 6e. The contents of main impurities in the product obtained after electrolysis are significantly reduced. Specifically, the Al content decreased from 71.9 to 21.3 ppm, the Mn content decreased from 4.9 to 3.2 ppm, the Fe content decreased from 361.5 to 23.1 ppm, and the Si content decreased from 715.4 to 57.3 ppm. The reduction in impurity content can be ascribed to the following factors. Oxide impurities such as Al_2_O_3_, MnO_2_, SiO_2_, and FeO exhibit relatively slow dissolution rates in H_2_SO_4_^58–60^, resulting in less leaching of impurity metal ions. Furthermore, even if a small amount of impurity metal ions enters the electrolyte, the reduction potentials of Al^3+^, Mn^2+^, and Fe^2+^ are more negative than those of the target metal ions (Fig. 6f), making them difficult to reduce at the cathode.

### Expanded experimental exploration and multi-faceted evaluation

**Fig. 7:**
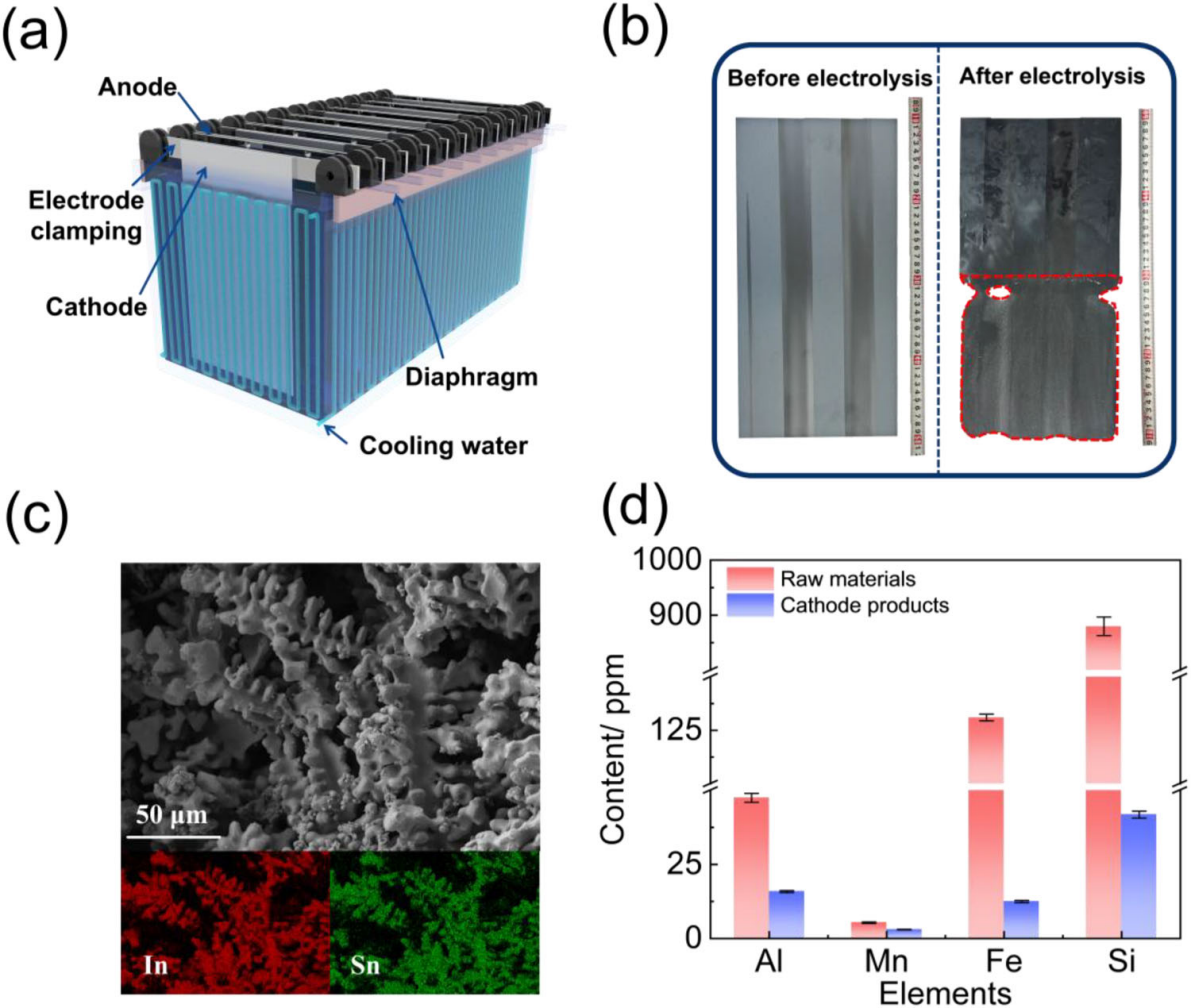
Expanded electrolysis recycling. **a** Schematic diagram of enlarged electrolysis cell; **b** Optical images of s-ITO before and after electrolysis (mass and size before electrolysis: 3.62 kg, 400 × 200 × 5 mm); **c** Scanning electron microscopy (SEM) image of cathode product and energy dispersive spectroscopy (EDS) images of In and Sn; **d** Impurity contents in s-ITO raw materials and electrolysis product. Data for (**d**) is presented as mean values ± standard deviation (SD) (*n* = 3).

Under the optimal process parameters, the current was scaled up to the hundred-ampere level for electrolysis. The technical advantages and application potential of this method were comprehensively evaluated by investigating the product purity, energy consumption and current efficiency. Figure 7a shows a schematic diagram of the self-designed industrial electrolyzer in the laboratory. s-ITO was used as the anode and a titanium plate as the cathode. The electrodes were fixed by electrode clamps and arranged in parallel at intervals. A 400-mesh diaphragm was installed between the cathode and the anode to prevent the migration of impurities from the anode to the cathode. The wall of the electrolyzer was equipped with water channels for the circulation of water, aiming to maintain temperature stability inside the electrolyzer. After electrolysis, the volume of the s-ITO anode immersed in the electrolyte decreased significantly (Fig. 7b and Supplementary Fig. 18). SEM images of the product (Fig. 7c) reveal that the In-Sn alloy exhibits a distinct dendritic morphology (Supplementary Fig. 19). The microstructure of the In-Sn alloy shows a porous and loose spongy texture, which is consistent with the grayish-white spongy appearance in the optical photographs. EDS results confirm the uniform distribution of indium and tin elements in the product. The impurity content in the electrolysis product is significantly reduced (Fig. 7d). Specifically, the Al content decreases from 47.5 to 15.8 ppm, the Mn content decreases from 5.3 to 3.0 ppm, the Fe content decreases from 131.0 to 12.5 ppm, the Si content decreases from 879.7 to 41.8 ppm. The impurity purification performance is not weakened with the scale-up of the recovery process. The mass of the product was 101.71 g, and the current efficiency was calculated to be 71.12%, which is close to that obtained in previous gram-scale recovery, confirming the stability of current efficiency after scaling up the technology. The time-voltage curve is displayed in Supplementary Fig. 20, with an average voltage of approximately 4.96 V. The electrolysis energy consumption was calculated to be 4.87 kWh kg^−1^ (calculation method is provided in Section 4 of Supplementary Information).

The electrolyte employed in this technology exhibits excellent reusability. After 10 cycles of repeated electrolysis (each cycle duration: 1.5 h, current density: 0.116 A cm^−2^), the H⁺ concentration in the electrolyte remained nearly constant (Supplementary Fig. 21a; the measurement method of H⁺ concentration is provided in Section 5 of Supplementary Information), whereas the concentrations of the main impurities increased gradually (Supplementary Fig. 21b). This observation indirectly demonstrates the impurity purification effect of the proposed process, in which most impurities are retained in the electrolyte during electrolysis. Accordingly, the contents of main impurities in the final cathodic product were still markedly reduced (Supplementary Fig. 21c), similar to those of the product obtained from a single electrolysis run. With an increasing number of cycles, the electrolysis process gradually stabilized, and the current efficiency in the later stage remained steady at approximately 71% (Supplementary Fig. 21d). These results clearly confirm that the proposed technology enables the repeated reuse of the electrolyte, thereby significantly reducing wastewater generation.

## Discussion

In summary, this paper proposes an electrochemical recovery strategy based on the charge-induced anodic deconstruction s-ITO. The results reveal that charge excitation enhances the activity of anodic oxygen ions and weakens their bonding with metal ions. The preferential escape of oxygen gives rise to oxygen vacancy defects and promotes the anodic release of indium and tin ions. The dissolved metal ions migrate toward the cathode, where they are selectively reduced to form high-purity In-Sn alloys based on the differences in electrochemical properties between the target metal ions and the impurity ions. Notably, NaCl acts as a key additive that significantly accelerates anodic dissolution and cathodic reduction by negatively shifting the anodic corrosion potential and promoting electron accumulation on the cathode surface. Favorable recovery performance can be achieved with an electrolyte consisting of 5.52 mol L^−1^ H_2_SO_4_ and 1.2 mol L^−1^ NaCl at a current density of 0.116 A cm^−2^. The addition of a certain amount of initial indium ions into the electrolyte can markedly improve the cathodic current efficiency. Hundred-ampere-level scale-up experiments further demonstrate that under the above conditions, the current efficiency is 71.12%, the energy consumption is 4.87 kWh kg^−1^, verifying the stability and industrialization potential of this technology. This technology is expected to not only open a route for the industrial-scale recovery of s-ITO, but also provide a solution to the global challenge of indium resource recycling.

## Methods

### Materials

S-ITO was derived from machining scraps and sputtering residues produced in our laboratory. In this study, all solutions employed for acid leaching, electrochemical dissolution, and electrolysis recovery were H_2_SO_4_ and H_2_SO_4_-NaCl solutions. H_2_SO_4_ (98%) was purchased from Shanghai Aladdin Biochemical Technology Co., Ltd. NaCl (purity 99.9%), In_2_(SO_4_)_3_ (purity 99.99%) and SnSO_4_ (purity 99%) were purchased from Shanghai McLean Biochemical Technology Co., Ltd.

### Acid leaching and anodic dissolution of s-ITO

Acid leaching and electrochemical dissolution were employed in H_2_SO_4_ and H_2_SO_4_-NaCl solutions. The concentration of H_2_SO_4_ in the H_2_SO_4_ solution was 5.52 mol L^−1^, while those of H_2_SO_4_ and NaCl in the H_2_SO_4_-NaCl solution were 5.52 and 1.0 mol L^−1^, respectively. The anode used for electrochemical dissolution was s-ITO obtained after wire cutting, with a size of approximately 40 × 8 × 5 mm, and the cathode was a high-purity titanium plate (99.99%) with a size of 100 × 10 × 2 mm, and the current density was 0.072 A cm^−2^. After acid leaching for 12 h and electrolysis for 1.5 h, the mass loss of s-ITO and the concentrations of indium and tin ions in the electrolyte were determined.

### Electrochemical measurement

Electrochemical testing was performed using an Autolab PGSTAT 302 N instrument, controlled by the Nova 2.1 software package, within a three-electrode system. In this configuration, a platinum rod served as the auxiliary electrode, while a saturated Ag/AgCl electrode acted as the reference electrode. LSV and Tafel measurements were performed in H_2_SO_4_ (5.52 mol L^−1^) and H_2_SO_4_-NaCl (5.52 mol L^−1^–1.2 mol L^−1^) solutions. For LSV measurements, s-ITO (effective area immersed in electrolyte is 0.08 cm^2^) and glassy carbon (D = 2 mm, effective area immersed in electrolyte is 0.0314 cm^2^) were used as the working electrodes at a scan rate of 0.1 V s^−1^. For Tafel measurements, s-ITO (effective area immersed in electrolyte is 0.08 cm^2^) was employed as the working electrode at a scan rate of 0.02 V s^−1^. CV and SWV were conducted in the same solutions with the addition of 0.01 mol L^−1^ In^3+^ and 0.01 mol L^−1^ Sn^2+^, using a Ti plate (effective area immersed in electrolyte is 0.1 cm^2^) as the working electrode. The scan rates for CV were 0.02, 0.04, 0.06, and 0.08 V s^−1^, and the frequency for SWV was 2 Hz.

### Electrolysis recovery of s-ITO

#### Exploration of electrolysis parameters

The anode employed here was s-ITO obtained after wire cutting, with dimensions of approximately 40 × 8 × 5 mm. The cathode was a high-purity titanium plate (99.99%) with dimensions of 100 × 10 × 2 mm. The electrolysis cell employed was a cylindrical glass reactor with a maximum working volume of 100 mL. The interelectrode distance between the anode and cathode was fixed at 2.0 cm, and no stirring was applied throughout the electrolysis process. A DC power supply (model UTP1310-Ⅱ) was used, with a voltage control range of 0–64 V and a current control range of 0–16 A.

Effect of H_2_SO_4_ concentration: Electrolysis was carried out at 0.116 A cm^−2^ for 1.5 h without initial In^3+^ addition, at a fixed NaCl concentration of 1.2 mol L^−1^. The concentrations of H_2_SO_4_ were 3.68, 4.60, 5.52, and 6.44 mol L^−1^, respectively.

Effect of NaCl concentration: Electrolysis was performed at 0.116 A cm^−2^ for 1.5 h without initial In^3+^ addition, at a fixed H_2_SO_4_ concentration of 5.52 mol L^−1^. The concentrations of NaCl were 0.86, 1.03, 1.20, 1.37, and 1.53 mol L^−1^, respectively.

Effect of current density: Electrolysis was conducted for 1.5 h without initial In^3+^ addition, at fixed concentrations of 5.52 mol L^−1^ H_2_SO_4_ and 1.2 mol L^−1^ NaCl. The current densities were set to 0.070, 0.093, 0.116, 0.140, and 0.163 A cm^−2^, respectively.

Effect of initial In^3+^ concentration: Electrolysis was carried out for 1.5 h at 0.116 A cm^−2^, at fixed concentrations of 5.52 mol L^−1^ H_2_SO_4_ and 1.2 mol L^−1^ NaCl, with different initial In^3+^ concentrations added. The concentrations of In^3+^ were 0.1, 0.2 and 0.3 mol L^−1^, respectively.

After electrolysis, the cathodic and anodic current efficiencies were calculated. The detailed calculation method is provided in Section 3 of Supplementary Information.

### Expanded electrolysis recovery of s-ITO

Expanded electrolysis was conducted in an electrolyte containing 5.52 mol L^−1^ H_2_SO_4_ and 1.2 mol L^−1^ NaCl. A constant current density of 0.116 A cm^−2^ (current is 100 A) was applied. A DC power supply (AGCO Cyber, PDC0317SH) was employed, with a voltage regulation range of 0–30 V and a current regulation range of 0–170 A. The anode was a s-ITO plate (mass: 3.62 kg; dimensions: 400 × 200 × 5 mm), and the cathode was a titanium plate with the same dimensions (Supplementary Fig. 22). The s-ITO anode was pre-electrolyzed at 100 A for 2 h to ensure sufficient ion accumulation in the electrolyte. Subsequently, a cathode of identical dimensions was installed, and electrolysis was conducted for 1 h.

After electrolysis, the current efficiency and energy consumption were calculated. The detailed calculation method is provided in Section 4 of Supplementary Information.

### Characterization

XPS (Thermo Fisher Scientific K-Alpha) was used to investigate the valence state changes of In, Sn, and O on the surface and profile of s-ITO before and after electrolysis.

For surface analysis of s-ITO before and after electrolysis: S-ITO was cleaned with deionized water and ethanol and then dried. S-ITO was electrolyzed in H_2_SO_4_-NaCl solution (5.52 mol L^−1^ H_2_SO_4_ and 1.2 mol L^−1^ NaCl) at a current density of 0.116 A cm^−2^ for 1.5 h. After electrolysis, the s-ITO was rinsed with deionized water and dried

For profile analysis of s-ITO after electrolysis: S-ITO was electrolyzed in H_2_SO_4_-NaCl solution (5.52 mol L^−1^ H_2_SO_4_ and 1.2 mol L^−1^ NaCl) at a current density of 0.116 A cm^−2^ for 1.5 h. After electrolysis, the s-ITO was rinsed with deionized water. The surface of the reacted s-ITO was polished with 2000-mesh sandpaper until the unreacted internal region was exposed, yielding a profile with the reaction zone at the edge and the unreacted zone in the inner part. Subsequently, the profile was cleaned with deionized water and ethanol, followed by drying. XPS analysis was performed using a laser spot with a size of 200 μm, and the analyzed regions were the reacted edge and the unreacted central region.

Raman spectroscopy (Horiba Aplora Plus confocal Raman spectrometer) with a 532 nm laser was employed to investigate surface change of s-ITO before and after electrolysis. S-ITO was used as the anode and electrolyzed in H_2_SO_4_-NaCl solution (5.52 mol L^−1^ H_2_SO_4_ and 1.2 mol L^−1^ NaCl) at a current density of 0.116 A cm^−2^ for 1.5 h. After electrolysis, the s-ITO anode was rinsed with deionized water and dried prior to characterization.

Ultraviolet photoelectron spectroscopy (UPS, Thermo Fisher Scientific Escalab, USA) was utilized to measure the work function, employing an Ar monatomic ion source with hv = 21.21 eV as the cleaning ion source. The pristine s-ITO was cleaned with deionized water and ethanol before being characterized as the reference sample. S-ITO was used as the anode and electrolyzed in H_2_SO_4_-NaCl solution (5.52 mol L^−1^ H_2_SO_4_ and 1.2 mol L^−1^ NaCl) at a current density of 0.116 A cm^−2^ for 1.5 h. After electrolysis, the s-ITO anode was rinsed with deionized water and dried prior to UPS characterization.

Electron paramagnetic resonance (EPR, Bruker Emxano, Germany) was employed to investigate the concentration of oxygen vacancies in s-ITO. The pristine s-ITO was cleaned with deionized water and ethanol for oxygen vacancy characterization. S-ITO was employed as the anode and electrolyzed in H_2_SO_4_-NaCl solution (5.52 mol L^−1^ H_2_SO_4_ and 1.2 mol L^−1^ NaCl) at a current density of 0.116 A cm^−2^ for 1.5 h. After electrolysis, the s-ITO anode was rinsed with deionized water and dried before quantitative analysis of oxygen vacancies.

Liquid-state NMR (Bruker 600 MHz, Germany) was employed to detect the change in the ^17^O spectra in different solutions at magnetic field strengths of 400/500 MHz, using deuterated chloroform as the solvent. All solutions were prepared at room temperature with the same volume, and their compositions were as follows: 5.52 mol L^−1^ H_2_SO_4_ and 0.01 mol L^−1^ indium ions (added as In_2_(SO_4_)_3_); 5.52 mol L^−1^ H_2_SO_4_, 0.01 mol L^−1^ In^3+^ (added as In_2_(SO_4_)_3_), and 1.2 mol L^−1^ NaCl; 5.52 mol L^−1^ H_2_SO_4_ and 0.01 mol L^−1^ tin ions (added as SnSO_4_); 5.52 mol L^−1^ H_2_SO_4_, 0.01 mol L^−1^ tin ions (added as SnSO_4_), and 1.2 mol L^−1^ NaCl.

Transmission electron microscopy (TEM, Thermo Fisher Scientific Talos F200X G2, USA) equipped with EDS system was used to investigate the cathode product, with an operating voltage of 200 kV. The cathode product was dispersed in ethanol and ultrasonicated for 5 min, followed by sample preparation on a copper grid.

SEM (Zeiss GeminiSEM 300) and the corresponding EDS were used to investigate the morphology, microstructure, and element distribution of the cathodic products.

XRD (Empyrean) with Cu Kα radiation was employed to identify the phase compositions of the s-ITO and the cathode product.

Inductively coupled plasma mass spectrometry (ICP-MS, Agilent 7800 Series) was used to determine the concentrations of impurity ions in the raw s-ITO, the recycled electrolyte, and the cathode product. For solid samples, acid digestion was conducted prior to ICP-MS analysis. The digestion solution was a mixture composed of 9 mL of aqua regia and 1 mL of hydrofluoric acid. Subsequently, the vessel was sealed, microwave digestion was carried out, and finally, the solution was diluted to within the calibrated linear range of the ICP-MS instrument for measurement.

### First-principles calculation

Perdew Wang (PW91) method^61^ in the Generalized Gradient Approximation (GGA) of Density Functional Theory is used as the exchange correlation functional, and the pseudopotential is selected as the ultrasoft pseudopotential^62^. Due to In_2_O_3_ being a strongly correlated electronic system, and GGA not fully considering the electronic interactions of strongly correlated electronic systems, whereas the Coulomb potentials of *In 4d*, *Sn 5d*, and *O 2p* electrons were taken into consideration. The Hubbard *U* values of *In 4d*, *O 2p*, and *Sn 5d* electrons are taken as 2, 5, and 5 eV, respectively^63,64^. In the geometric structure optimization, the plane wave truncation energy is set to 380 eV, and the Brillouin zone k-point is taken as 2 × 2 × 2^65^. After 100 iterations, the energy convergence range can be ensured to be less than 1.0 × 10^−5^ eV atom^−1^, and the force convergence of each atom is less than 0.03 eV Å^–1^.

### Calculation of ionic complex structure

Visual MINTEQ 3.1 software was employed to investigate the ionic complex structure. The ion concentration ratio was adjusted according to the optimal process conditions, with the H_2_SO_4_ concentration of 0.00552 mol L^−1^, NaCl concentration of 0.0012 mol L^−1^, In ion concentration of 0.0001 mol L^−1^, and Sn ion concentration of 0.0001 mol L^−1^. The pH calculation range was set to 0–7 with a calculation interval of 0.1.

### Statistics and reproducibility

To validate the reproducibility and reliability of the experimental results, error bars are incorporated into all bar charts and point-line graphs associated with the recycling experiments and compositional characterization. The data points represent the average of three sets of experimental results, and the error bars represent the standard deviation of three experimental results. Acid leaching and electrolysis experiments were conducted in three independent runs to account for and minimize unforeseen experimental variability. For ICP-based elemental quantification of raw materials, recovered products, and electrolyte compositions, each sample was analyzed in triplicate under identical testing conditions to ensure the accuracy and precision of the reported elemental concentrations.

### Reporting summary

Further information on research design is available in the Nature Portfolio Reporting Summary linked to this article.

## Supplementary information

Supplementary Information

Reporting Summary

Transparent Peer Review file

## Source data

Source Data

## Data Availability

The data supporting the findings of this study are included in the published article and its Supplementary Information or available from the corresponding authors on request. Source data are provided with this paper.
