# Supplementary material for: Electrochemical upcycling of spent ITO via charge-induced deconstruction
Source: Nat Commun. 2026 Jul 28;17:7562. doi: 10.1038/s41467-026-75921-w (PMC13415520; doi:10.1038/s41467-026-75921-w)
Supplement: Supplementary file 1 — Supplementary Information [file 41467_2026_75921_MOESM1_ESM.pdf]

Supplementary Information

Electrochemical upcycling of spent ITO via charge-induced deconstruction

Supplementary Information Content

Section 1: Calculation of theoretical reduction potential

Section 2: Calculation of mass loss ratio

Section 3: Lattice optimization and energy calculation of the basic model

Section 4: Calculation of current efficiency

Section 5: Calculation of energy consumption

Section 6: Reusability experiment of the electrolyte

Section 7: Figures

Section 8: Supplementary references

Section 1: Calculation of theoretical reduction potential

The theoretical reduction potential was obtained by the following formula, where ΔG was derived from HSC Chemistry 6.0.

$$\Delta G = -n\mathrm{F}E \tag{1}$$

Where ΔG *is* Gibbs free energy change, kJ; n is number of electrons transferred in the reaction; F is Faraday constant, 96485 C mol^{-1}; E is theoretical reduction potential.

Section 2: Calculation of mass loss ratio

The mass loss ratio is obtained by the following formula.

$$\eta_1 = \frac{m_b - m_a}{m_b} \tag{2}$$

Where η_1 is the mass loss ratio, %; m_a is the mass of s-ITO after treatment; m_b is the mass of s-ITO before processing.

Section 3: Lattice optimization and energy calculation of the basic model

In_2O_3 has a cubic structure and belongs to the point group *Ia3*[1], with the unit cell encompassing 80 atoms, equivalent to 16 In_2O_3 molecules. Indium ions occupy two different coordinate positions in the lattice, called "8b" and "24d" positions, while the position of oxygen ions is called "48e", as shown in **Supplementary Figure 5**. After geometric structure optimization, the lattice constants of In_2O_3 doped with different Sn were obtained, the lattice constant of In_2O_3 is 10.202 Å, which is close to the experimental value of 10.118 Å with an

error of less than 1%, indicating the reliability of the calculated results[2]. After Sn doping, the lattice constant slightly increases, but remains between 10.2 Å and 10.3 Å. The reason is that Sn doping generates residual stress in the lattice, leading to lattice distortion, and Sn^{4+} contributes one more free electron than In^{3+}, which enhances the repulsion between excess positive charges and increases the total energy of the system, resulting in an increase in volume[3]. Upon achieving structural convergence, the band gap (E_g) of In_2O_3 was determined to be 2.594 eV (**Supplementary Figure 6a**), which is consistent with the results reported in the literature. The total density of states (TDOS) and partial density of states (PDOS) were calculated to understand the band structure of In_2O_3 and the contribution of each atomic orbital. The calculation results in **Supplementary Figure 6b** indicate that the top of the valence band in In_2O_3 is mainly contributed by the *O 2p* and *In 4d* states, while the valence band at the low-energy end is primarily derived from the interaction between the *O 2s*, *In 5s*, and *In 5p* orbitals. The bottom of the conduction band is mainly attributed to the contribution of the *In 5s* orbitals. However, when Sn is doped at a concentration of 9.375 at.% to replace In at the 8b site, E_g of ITO decreases to 2.245 eV, as shown in **Supplementary Figure 6c**. The band structure shifts toward lower energy levels, and the Fermi level (E_F) moves toward the conduction band, exhibiting characteristics of metallic conductivity. Thus, the E_g of ITO decreases, accompanied by an increase in electrical conductivity. After Sn doping, the density of states (DOS) shifts toward lower energy, and E_F enters the conduction band, which is consistent with the band structure results shown in **Supplementary Figure 6d**. Notably, electrons in the In 5s and Sn 5s orbitals mainly occupy the bottom of the conduction band, which explains the enhanced electrical properties of ITO after Sn doping. Since Sn^{4+} donates one additional free electron compared to In^{3+}, Sn doping increases the number of free carriers. The donor levels formed near E_F degenerate with the conduction band, forming a so-called degenerate semiconductor and enhancing electrical conductivity. This observation is consistent with the intrinsic reduction in the E_g of ITO after Sn doping, as revealed by the band structure analysis. The model with a Sn doping concentration of 9.375 at.% matches the mass ratio of In_2O_3 to SnO_2 (9:1) in ITO; therefore, this doping concentration was selected for the construction of the ITO model and subsequent calculations.

Section 4: Calculation of current efficiency

Current efficiency is an important indicator for measuring the electrolysis effect, and it was calculated according to the following formula[4]:

$$m=\frac{It\mathrm{M}}{\mathrm{F}z} \tag{3}$$

$$\eta_2=\frac{m_I}{m} \tag{4}$$

In the formula, m and m_I are the theoretical and actual masses of the product obtained by electrolysis, g; I represents the current, A; t is the electrolysis time, s; M is the atomic mass; Z represents the number of moles of transferred electrons.

A calculation was performed using a certain electrolysis process as an example. The electrolysis time was 1.5 h, the current density was 0.116 A cm^{-2} (with a current of 0.5 A), and the actual mass of the cathodic product (In-Sn alloy) was 0.5555 g. According to the XRD results, the atomic ratio of In to Sn in the obtained alloy was 0.959: 0.041. The relative atomic mass of In is 114.82, and that of Sn is 118.71; thus, the relative atomic mass of the In-Sn alloy was calculated as 114.82×0.959 + 118.71×0.041 = 114.98. The number of electrons transferred during the reduction of In^{3+} to metal In is 3, and that during the reduction of Sn^{4+} to metal Sn is 4; therefore, the total number of electrons transferred during the reduction to form the alloy was 3×0.959 + 4×0.041 = 3.041. Based on the above formula, the theoretical product mass was 1.058 g, and the current efficiency was 52.5%.

When the influence of a small amount of Sn was not considered and pure indium was assumed to be obtained, the number of transferred electrons and the relative atomic mass were set as 3 and 114.82, respectively. The theoretical product mass was 1.071 g and the calculated current efficiency was 51.87%. The difference between this value and the result obtained using the In-Sn alloy was very small. In view of the low Sn content in the cathodic product, using In instead of the In-Sn alloy for current efficiency calculation had little impact on the final result and would not affect the trend of efficiency change. Therefore, to simplify the calculation of current efficiency, In was used instead of the In-Sn alloy. In the calculation, the value of z was set to 3, and the relative atomic mass was 114.82.

Section 5: Calculation of energy consumption

According to the expansion experiment, the current during the electrolysis process is 100 A, and 101.71 g of In-Sn alloy can be recovered after 1 h of electrolysis. The average voltage of the entire electrolysis process is approximately 4.96 V. The energy consumption of s-ITO

recycling can be calculated according to the following formula[5]:

$$W = U \times I \times t = 0.496\ \text{kWh} \quad (5)$$

W represents energy consumption, kwh; *U* is the voltage during electrolysis, V.

The energy consumption required to recover 101.71 g of In-Sn alloy is 0.496 kWh, indicating that the energy consumption required to recover 1 kg of s-ITO is 4.87 kWh.

Section 6: Reusability experiment of the electrolyte

To evaluate the reusability of the electrolyte, the same electrolysis process was continuously repeated in the original electrolyte after each electrolysis run, with a total of 10 consecutive runs conducted. The duration of each electrolysis is 1.5 h. The electrolyte was composed of 100 mL of 5.52 mol L^{-1} H_2SO_4 and 1.2 mol L^{-1} NaCl. For each electrolysis run, cathodes (titanium plates) and anodes (s-ITO) with the same dimensions were employed, and both the electrode spacing and current density (0.116 A cm^{-2}) were maintained constant. Impurity analysis was conducted on the products and electrolyte collected subsequent to the second run, accompanied by quantitative determination of the hydrogen ion concentration within the electrolyte. Subsequent sampling and characterization were implemented at intervals of every other cycle throughout the entire electrolysis process.

The determination of hydrogen ion concentration was achieved by measuring the pH value of the diluted solution: 100 μL of the electrolyte was pipetted and diluted to 10 mL with deionized water, followed by pH measurement using a PHS-25 benchtop pH meter, and finally the hydrogen ion concentration in the solution was calculated according to Formula 5. The calculated H^+ concentration was then multiplied by 100 to obtain the H^+ concentration in the original electrolyte.

$$\text{pH} = -\lg C \quad (6)$$

where *C* is the hydrogen ion concentration (mol L^{-1}).

Section 7: Figures

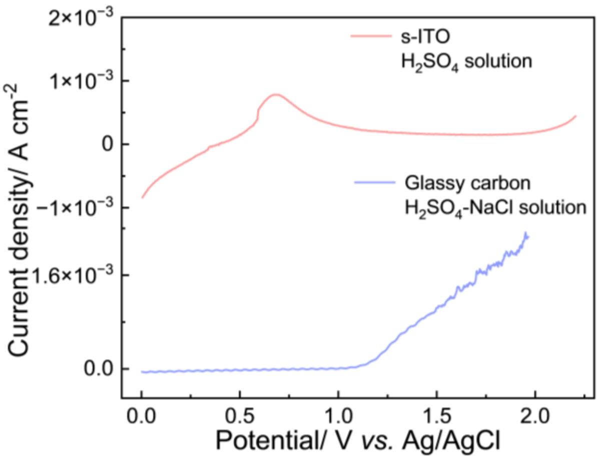

Supplementary Figure 1. Linear sweep voltammetry (LSV) curves obtained using s-ITO and glassy carbon as working electrodes in H_2SO_4 and H_2SO_4-NaCl solutions (H_2SO_4 concentration: 5.52 mol L^{-1}, NaCl concentration: 1.2 mol L^{-1}, scan rate: 0.1 V s^{-1}).

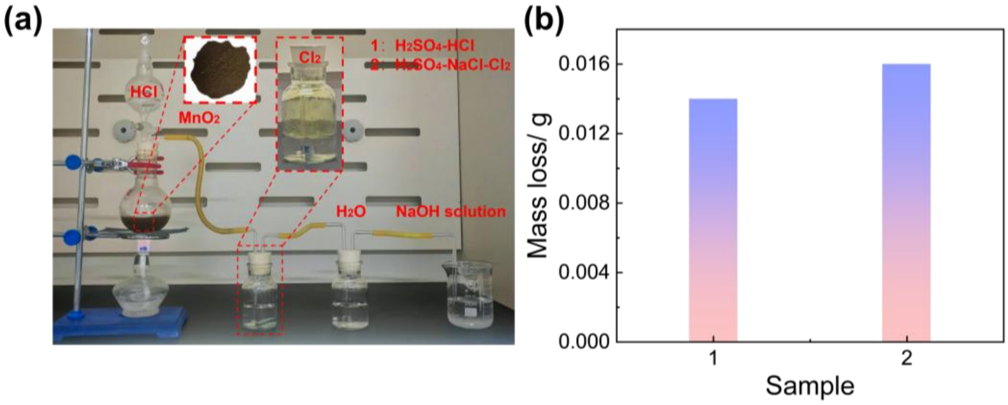

Supplementary Figure 2. Chlorine generation and leaching experiments under different chlorine species conditions. (a) Cl_2 generation device (reaction equation: $4HCl+MnO_2=MnCl_2+Cl_2(g)+2H_2O$; the concentration of HCl is 36%-38%, 30 mL (in excess); 5.22 g of MnO_2 (approximately 0.06 mol)); (b) Acid leaching results of s-ITO under different conditions (1: 5.52 mol L^{-1} H_2SO_4+1.2 mol L^{-1} HCl, 2: 5.52 mol L^{-1} H_2SO_4+1.2 mol L^{-1} NaCl+0.06 mol Cl_2; Duration: 1.5 h).

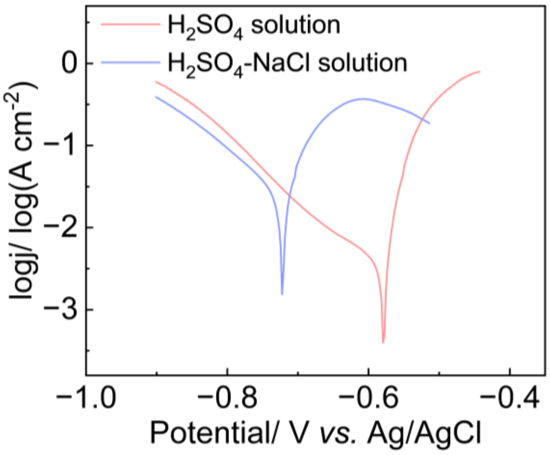

Supplementary Figure 3. Tafel curves of s-ITO in H_2SO_4 solution before and after adding NaCl (H_2SO_4 concentration: 5.52 mol L^{-1}, NaCl concentration: 1.2 mol L^{-1}, scan rate: 0.02 V s^{-1}).

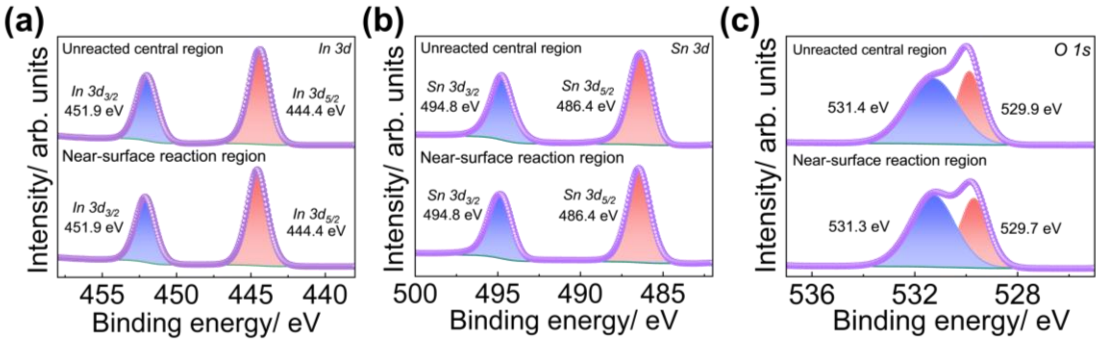

Supplementary Figure 4. Microzone X-ray photoelectron spectroscopy (XPS) spectra of s-ITO profile after electrolysis: (a) *In 3d*, (b) *Sn 3d*, (c) *O 1s*.

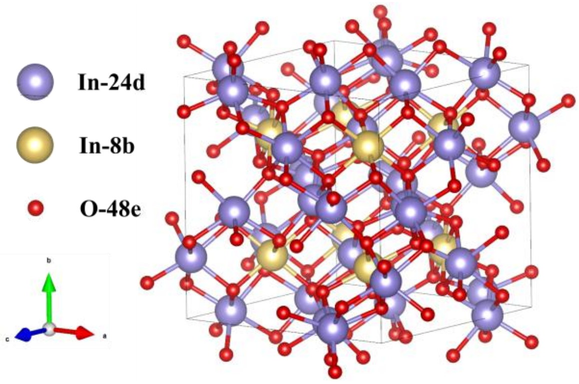

Supplementary Figure 5. The In_2O_3 model used for calculation.

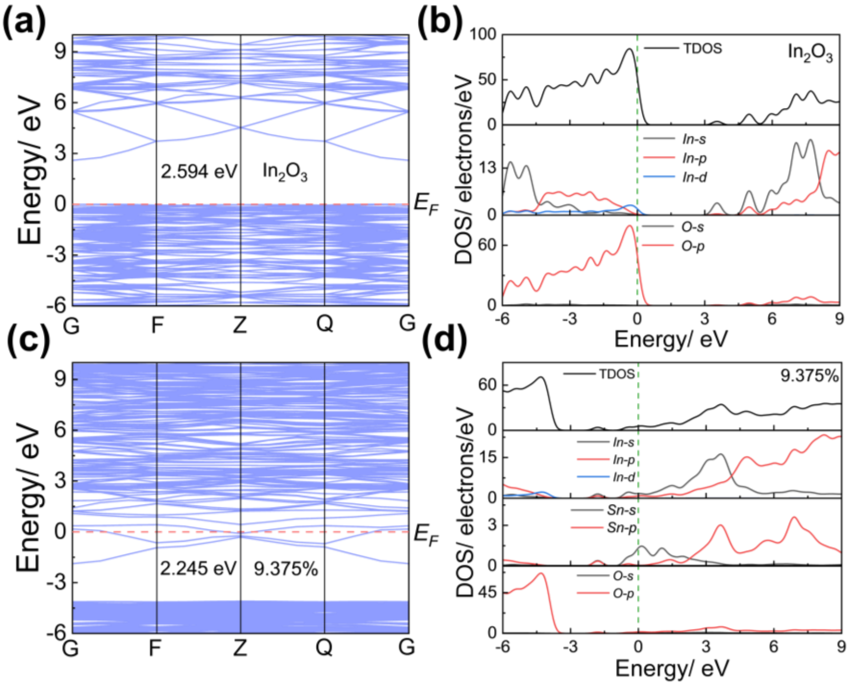

Supplementary Figure 6. Calculation of In_2O_3 and Sn doped In_2O_3. (a) Band structure diagram of In_2O_3; (b) Density of states diagram of In_2O_3; (c) Band structure diagram of ITO (SnO_2 doped In_2O_3: 9.375 at. %); (d) Density of states diagram of ITO (SnO_2 doped In_2O_3: 9.375 at.%).

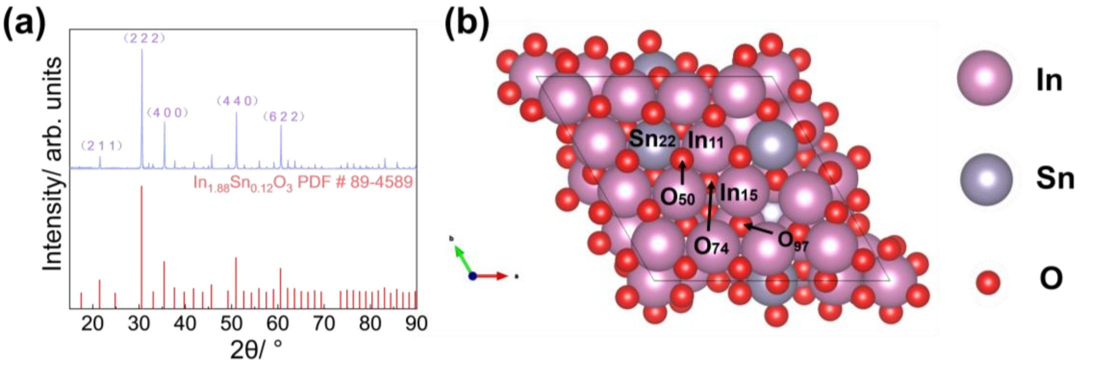

Supplementary Figure 7. Crystal orientation of s-ITO. (a) X-ray diffraction (XRD) pattern of s-ITO; (b) Model of the (2 2 2) crystal plane orientation of s-ITO.

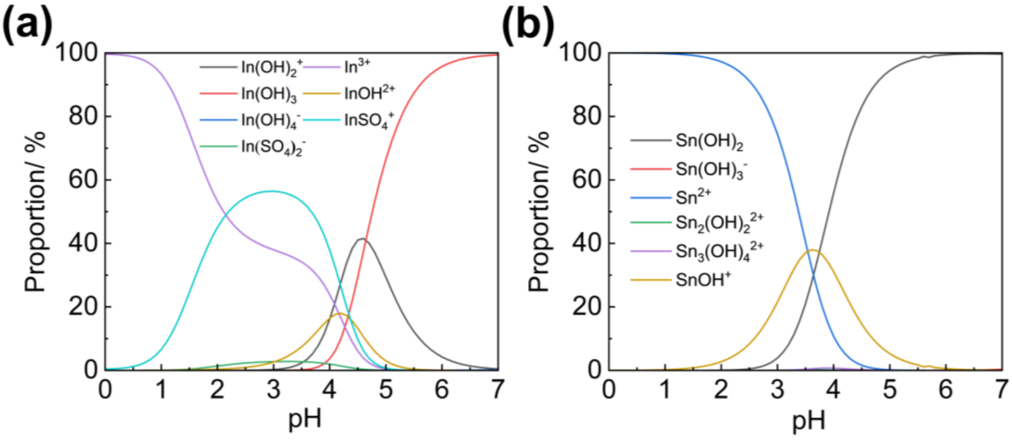

Supplementary Figure 8. Ion distribution map in H_2SO_4 solution drawn using Visual MINTEQ 3.1 (H_2SO_4: 0.0552 mol L^{-1}): (a) Indium ions (indium ion concentration: 0.001 mol L^{-1}), (b) Tin ions (tin ions concentration: 0.001 mol L^{-1}).

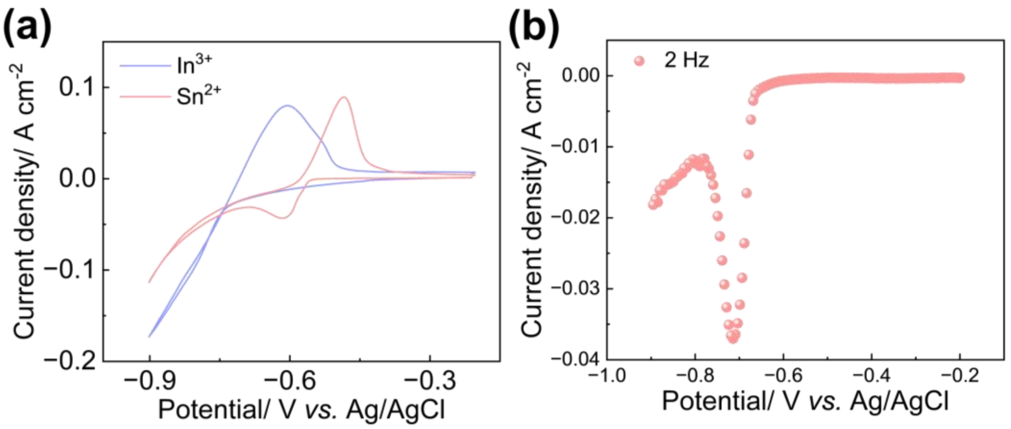

Supplementary Figure 9. Cyclic voltammetry (CV) and square wave voltammetry (SWV) curves in 5.52 mol L^{-1} H_2SO_4 solution. (a) CV curves of indium ion and tin ion (indium ion concentration: 0.01 mol L^{-1}, Sn^{2+} concentration: 0.01 mol L^{-1}, scan rate: 0.1 V s^{-1}); (b) SWV curve of indium ion (indium ion concentration: 0.01 mol L^{-1})

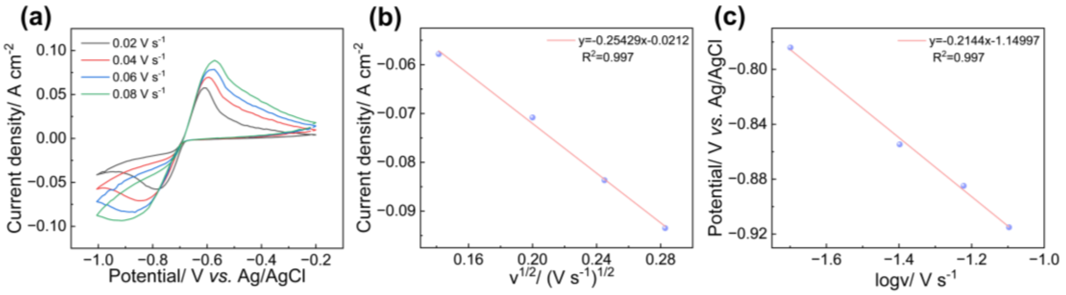

Supplementary Figure 10. Cyclic voltammetry (CV) and derived curves in H_2SO_4-NaCl solution containing indium ions (indium ion concentration: 0.01 mol L^{-1}, H_2SO_4 concentration: 5.52 mol L^{-1}, NaCl concentration: 1.2 mol L^{-1}): (a) CV curves at different scan rates; (b) The fitting curve of peak current and square root of scan rate; (c) The fitting curve of potential and logarithm of the scan rate.

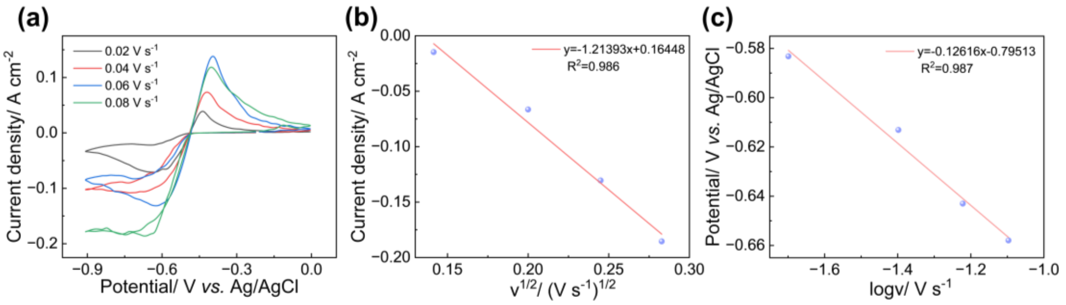

Supplementary Figure 11. Cyclic voltammetry (CV) and derived curves in H_2SO_4-NaCl solution containing tin ions (tin ion concentration: 0.01 mol L^{-1}, H_2SO_4 concentration: 5.52 mol L^{-1}, NaCl concentration: 1.2 mol L^{-1}): (a) CV curves at different scan rates; (b) The fitting curve of peak current and square root of scan rate; (c) The fitting curve of potential and logarithm of the scan rate.

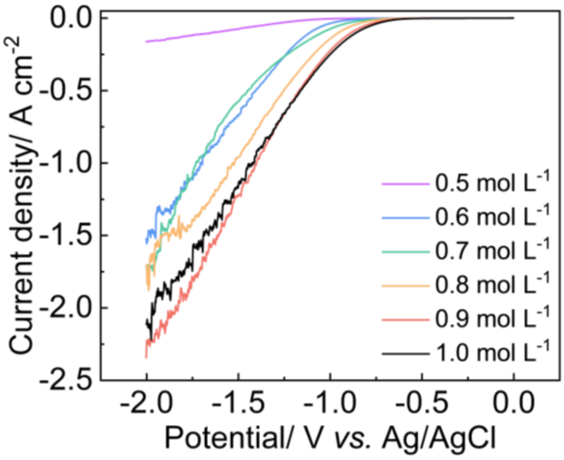

Supplementary Figure 12. Linear sweep voltammetry (LSV) curves with Ti working electrode in H_2SO_4 solution with different concentrations (scan rate: 0.1 V s^{-1}).

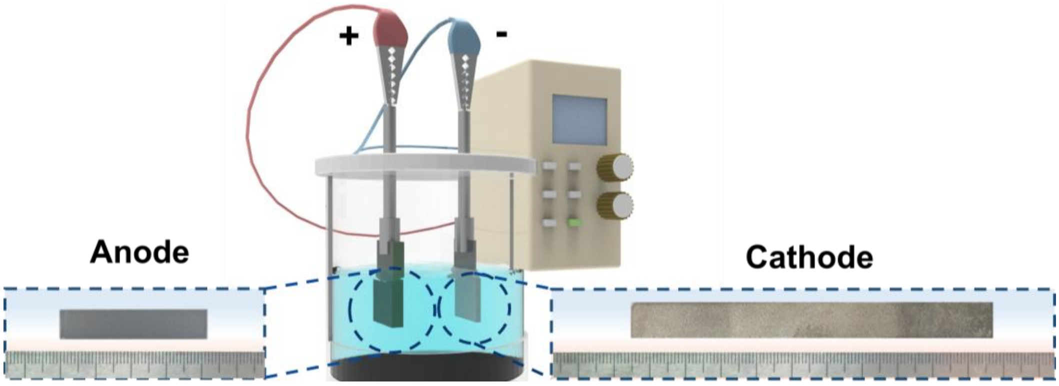

Supplementary Figure 13. Schematic diagram of electrolysis device and optical photos of s-ITO anode and Ti cathode.

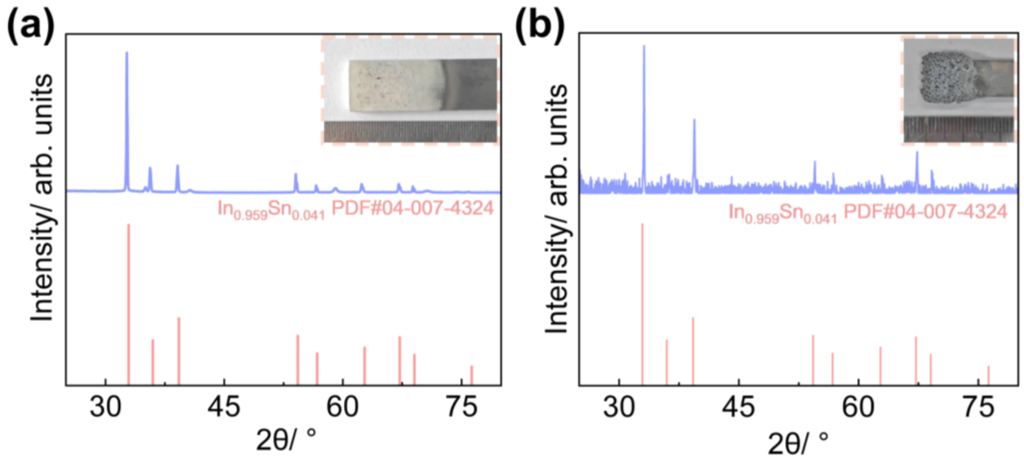

Supplementary Figure 14. X-ray diffraction (XRD) pattern of cathodic products (Inserts: Optical photos of cathode products): (a) H_2SO_4 solution, (b) H_2SO_4-NaCl solution (H_2SO_4 concentration: 5.52 mol L^{-1}, NaCl concentration: 1.0 mol L^{-1}, current density: 0.072 A cm^{-2}, duration: 1.5 h).

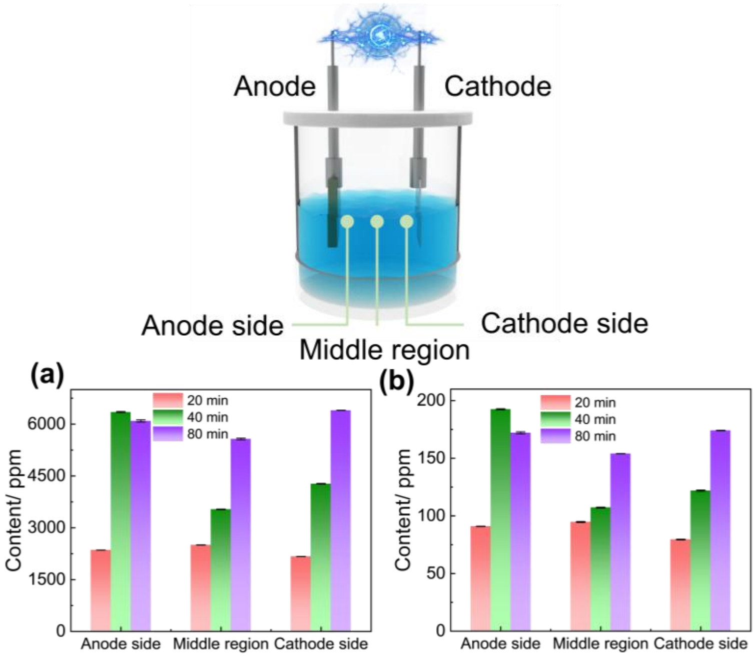

Supplementary Figure 15. Concentrations of indium and tin ions at different electrolysis time in H_2SO_4-NaCl aqueous solution using spent ITO target as the anode (H_2SO_4 concentration: 5.52 mol L^{-1}; NaCl concentration: 1.2 mol L^{-1}; current density: 0.116 A cm^{-2}): (a) Indium ion concentrations; (b) Tin ion concentrations. Data for (a-b) are presented as mean values ± standard deviation (SD) (n = 3).

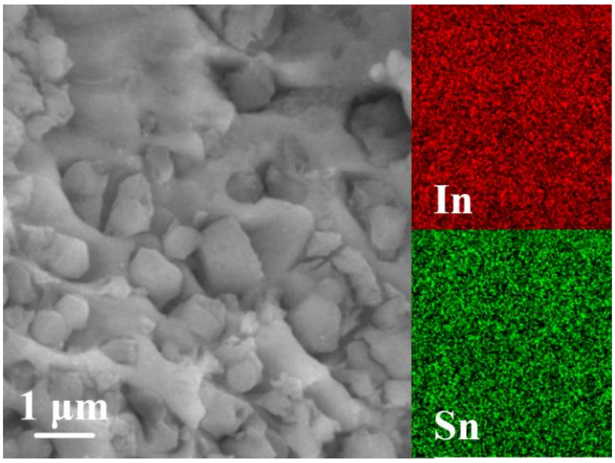

Supplementary Figure 16. Scanning electron microscopy (SEM) images of cathode products and energy dispersive spectroscopy (EDS) spectra of In and Sn elements (current density: 0.116 A cm^{-2}, duration: 1.5 h, H_2SO_4 concentration: 5.52 mol L^{-1}, NaCl concentration: 1.2 mol L^{-1}).

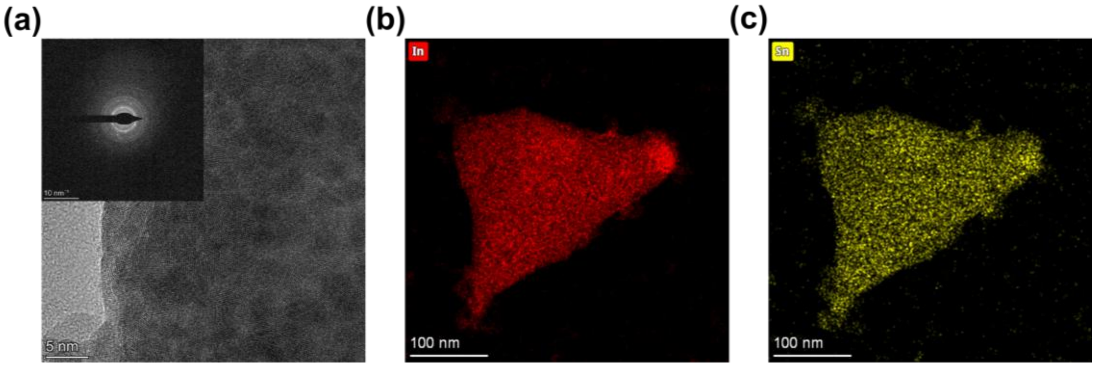

Supplementary Figure 17. Cathode product analysis (Current density: 0.116 A cm^{-2}, duration: 1.5 h, H_2SO_4 concentration: 5.52 mol L^{-1}, NaCl concentration: 1.2 mol L^{-1}): (a) Transmission electron microscopy (TEM) images of cathode products (Insert: Electron diffraction patterns); Energy dispersive spectroscopy (EDS) spectra of (b) In and (c) Sn elements.

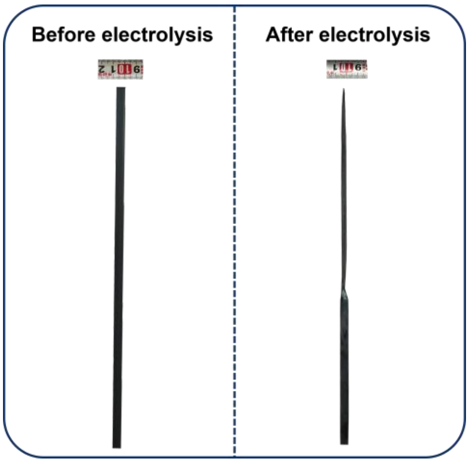

Supplementary Figure 18. Optical photos of s-ITO anode before and after expanded electrolysis.

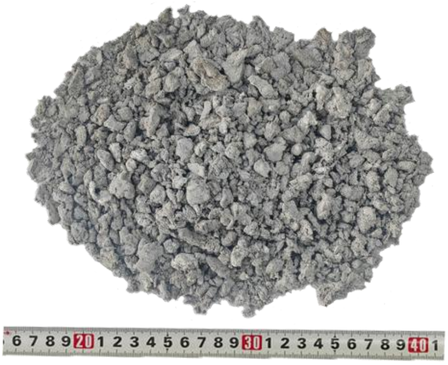

Supplementary Figure 19. Optical photograph of cathode product.

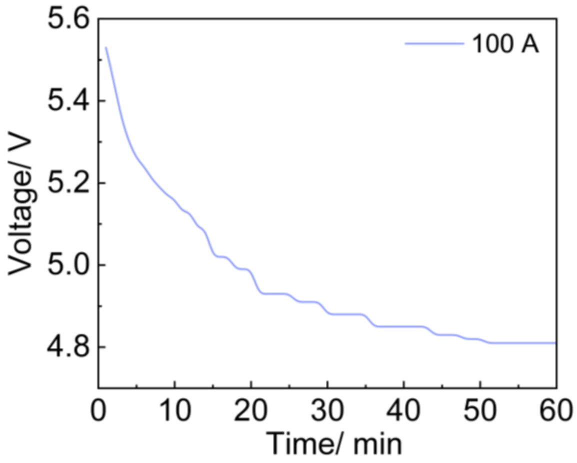

Supplementary Figure 20. Time-voltage curve of s-ITO electrolysis process.

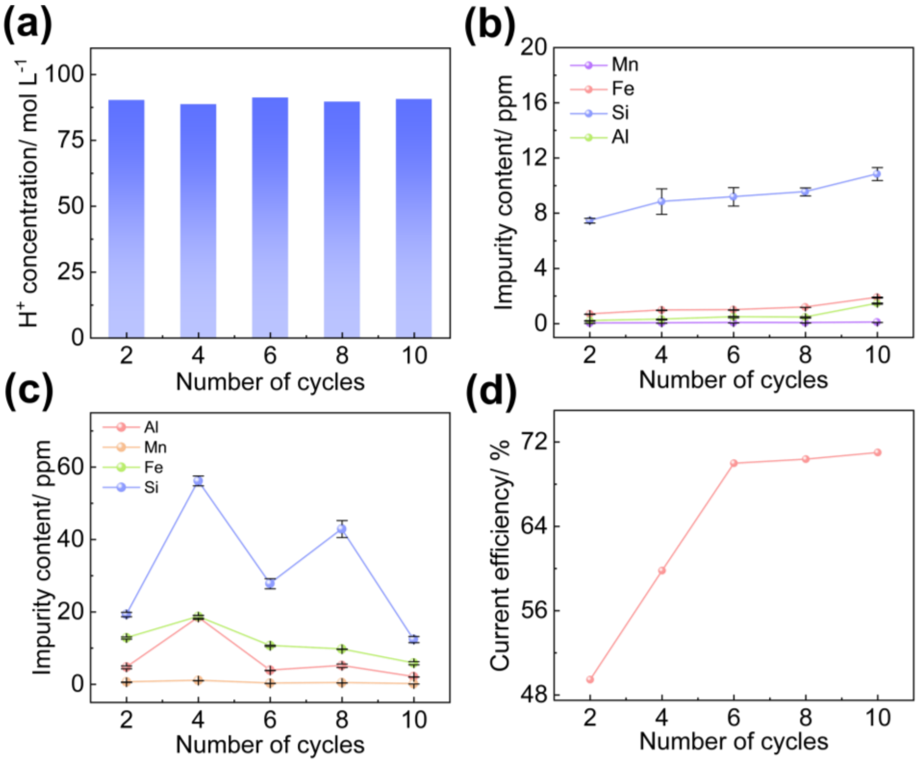

Supplementary Figure 21. Evaluation of electrolyte stability under multi-cycle electrolysis

operation (the duration of each electrolysis is 1.5 h and current density is 0.116 A cm^{-2}; the electrolyte is composed of 100 mL of 5.52 mol L^{-1} H_2SO_4 and 1.2 mol L^{-1} NaCl.). (a) Changes of H^+ concentration of electrolyte; The changes of the main impurity content after 10 cycles of electrolysis: (b) electrolyte, (c) cathode product; (d) Cathode current efficiency. Data for (b-c) are presented as mean values ± standard deviation (SD) (n = 3).

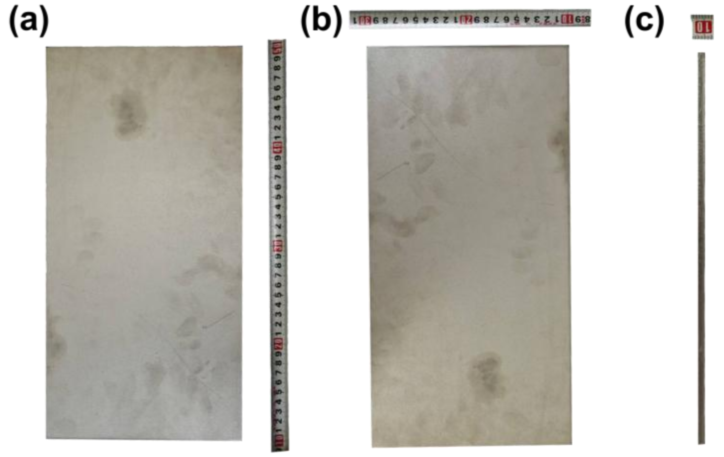

Supplementary Figure 22. Optical photos of the cathode titanium plate used in expanded electrolysis: (a) length (400 mm), (b) width (200 mm), (c) thickness (5 mm).

Section 8: Supplementary references

1. Ziemba, M., Schumacher, L. & Hess, C. Reduction behavior of cubic In_2O_3 nanoparticles by combined multiple *In Situ* spectroscopy and DFT. *J. Phys. Chem. Lett.* **12**, 3749–3754 (2021).

2. Song, L. *et al.* Sr-doped cubic In_2O_3 /rhombohedral In_2O_3 homojunction nanowires for highly sensitive and selective breath ethanol sensing: Experiment and DFT simulation studies. *ACS Appl. Mater. Interfaces* **12**, 1270–1279 (2020).

3. Schimpf, A. M., Lounis, S. D., Runnerstrom, E. L., Milliron, D. J. & Gamelin, D. R. Redox Chemistries and plasmon energies of photodoped In_2O_3 and Sn-doped In_2O_3 (ITO) nanocrystals. *J. Am. Chem. Soc.* **137**, 518–524 (2015).

4. Hong, S. *et al.* Determination of impressed current efficiency during accelerated corrosion of reinforcement. *Cement and Concrete Composites* **108**, 103536 (2020).

5. Maksic, A. D., Miulovic, S. M., Nikolic, V. M., Perovic, I. M. & Marceta Kaninski, M. P. Energy consumption of the electrolytic hydrogen production using Ni–W based activators—Part I. *Applied Catalysis A: General* **405**, 25–28 (2011).
