# Supplementary material for: Electrochemical upcycling of spent ITO via charge-induced deconstruction
Source: Nat Commun. 2026 Jul 28;17:7562. doi: 10.1038/s41467-026-75921-w (PMC13415520; doi:10.1038/s41467-026-75921-w)
Supplement: Supplementary file 2 — Reporting Summary [file 41467_2026_75921_MOESM2_ESM.pdf]

nature portfolio

Corresponding author(s): Jianxun Song, Shaolong Li

Last updated by author(s): Mar 28, 2026

Reporting Summary

Nature Portfolio wishes to improve the reproducibility of the work that we publish. This form provides structure for consistency and transparency in reporting. For further information on Nature Portfolio policies, see our Editorial Policies and the Editorial Policy Checklist.

Statistics

For all statistical analyses, confirm that the following items are present in the figure legend, table legend, main text, or Methods section.

n/a	Confirmed	
☐	☒	The exact sample size (*n*) for each experimental group/condition, given as a discrete number and unit of measurement
☒	☐	A statement on whether measurements were taken from distinct samples or whether the same sample was measured repeatedly
☒	☐	The statistical test(s) used AND whether they are one- or two-sided<br>*Only common tests should be described solely by name; describe more complex techniques in the Methods section.*
☒	☐	A description of all covariates tested
☒	☐	A description of any assumptions or corrections, such as tests of normality and adjustment for multiple comparisons
☐	☒	A full description of the statistical parameters including central tendency (e.g. means) or other basic estimates (e.g. regression coefficient) AND variation (e.g. standard deviation) or associated estimates of uncertainty (e.g. confidence intervals)
☒	☐	For null hypothesis testing, the test statistic (e.g. *F*, *t*, *r*) with confidence intervals, effect sizes, degrees of freedom and *P* value noted<br>*Give P values as exact values whenever suitable.*
☒	☐	For Bayesian analysis, information on the choice of priors and Markov chain Monte Carlo settings
☒	☐	For hierarchical and complex designs, identification of the appropriate level for tests and full reporting of outcomes
☒	☐	Estimates of effect sizes (e.g. Cohen's *d*, Pearson's *r*), indicating how they were calculated

Our web collection on statistics for biologists contains articles on many of the points above.

Software and code

Policy information about availability of computer code

Data collection	Our research does not involve computer code.
Data analysis	Our research does not involve computer code.

For manuscripts utilizing custom algorithms or software that are central to the research but not yet described in published literature, software must be made available to editors and reviewers. We strongly encourage code deposition in a community repository (e.g. GitHub). See the Nature Portfolio guidelines for submitting code & software for further information.

Data

Policy information about availability of data

All manuscripts must include a data availability statement. This statement should provide the following information, where applicable:

- Accession codes, unique identifiers, or web links for publicly available datasets
- A description of any restrictions on data availability
- For clinical datasets or third party data, please ensure that the statement adheres to our policy

All data generated or analyzed during this study are included in this article (and its supplementary information files), and the data supporting the results of this research is publicly available.

Research involving human participants, their data, or biological material

Policy information about studies with human participants or human data. See also policy information about sex, gender (identity/presentation), and sexual orientation and race, ethnicity and racism.

Reporting on sex and gender	This paper does not involve human research participants.
Reporting on race, ethnicity, or other socially relevant groupings	This paper does not involve human research participants.
Population characteristics	This paper does not involve human research participants.
Recruitment	This paper does not involve human research participants.
Ethics oversight	Zhengzhou University

Note that full information on the approval of the study protocol must also be provided in the manuscript.

Field-specific reporting

Please select the one below that is the best fit for your research. If you are not sure, read the appropriate sections before making your selection.

☐ Life sciences ☐ Behavioural & social sciences ☒ Ecological, evolutionary & environmental sciences

For a reference copy of the document with all sections, see nature.com/documents/nr-reporting-summary-flat.pdf

Ecological, evolutionary & environmental sciences study design

All studies must disclose on these points even when the disclosure is negative.

Study description	This research investigated the electrolytic recovery mechanism and ionic reaction process of s-ITO, and conducted in-depth research on process parameters, product morphology, and scale-up stability. The principle, procedure and advantages of an s-ITO electrolytic recovery process were comprehensively elaborated. The experimental work was divided into four parts: anode, cathode, process optimization and scale-up experiments. The same solution was used for both testing and electrolysis. Electrolytic experiments were carried out based on the control variable method.
Research sample	This research focuses on the research of recycling mechanism, electrolysis parameters, current efficiency, and product impurity content. The raw materials are all laboratory prepared s-ITO, and the composition and structure of the raw materials are consistent, without involving other variability research samples.
Sampling strategy	This experiment does not involve sampling strategy.
Data collection	The data was collected and analyzed by the first author Rongcen Zhao, and verified by the corresponding author Shaolong Li. The data is collected after the same processing to avoid errors caused by operations.
Timing and spatial scale	The data collection in this experiment is only related to the experimental process itself and has no effect on seasonal time and spatial changes.
Data exclusions	No data were excluded from the research protocol to ensure data integrity.
Reproducibility	To verify reproducibility, key experiments were repeated multiple times in independent batches. All repeated experimental attempts were successful, and the target product was successfully obtained in each case. For the exploration of process parameters, the experimental results for current efficiency under identical parameters were consistent across batches (variation < 2%).
Randomization	Randomization is not related to this observational study, as the samples were obtained during experimental procedures and the exploration of process parameters was conducted using the controlled variable method.
Blinding	Blinding is not related to this study, as all measurements were conducted using tools, eliminating human observer bias.

Did the study involve field work? ☐ Yes ☒ No

Reporting for specific materials, systems and methods

We require information from authors about some types of materials, experimental systems and methods used in many studies. Here, indicate whether each material, system or method listed is relevant to your study. If you are not sure if a list item applies to your research, read the appropriate section before selecting a response.

Materials & experimental systems

n/a	Involved in the study
☒	☐ Antibodies
☒	☐ Eukaryotic cell lines
☒	☐ Palaeontology and archaeology
☒	☐ Animals and other organisms
☒	☐ Clinical data
☒	☐ Dual use research of concern
☒	☐ Plants

Methods

n/a	Involved in the study
☒	☐ ChIP-seq
☒	☐ Flow cytometry
☒	☐ MRI-based neuroimaging

Plants

Seed stocks	This research does not involve seeds or plant materials.
Novel plant genotypes	This research does not involve genetic related content.
Authentication	This research does not involve the identification process of plant seeds or new genotypes.
