# Supplementary material for: Electrochemical upcycling of spent ITO via charge-induced deconstruction
Source: Nat Commun. 2026 Jul 28;17:7562. doi: 10.1038/s41467-026-75921-w (PMC13415520; doi:10.1038/s41467-026-75921-w)
Supplement: Supplementary file 3 — Transparent Peer Review file [file 41467_2026_75921_MOESM3_ESM.pdf]

# Peer Review File

# Electrochemical upcycling of spent ITO via charge-induced deconstruction

Corresponding Author: Dr Shaolong Li

**This file contains all reviewer reports in order by version, followed by all author rebuttals in order by version.**

Version 0:

Reviewer comments:

Reviewer #1

(Remarks to the Author)
First, I would like to congratulate the authors on the extensive and good work. The topic is relevant, and the use of electrochemistry to improve and enable leaching processes is an interesting approach.

The last author, Jianxun Song, has similar publications in the area. Like the recently published article: https://doi.org/10.1016/j.susmat.2025.e01710. A first question is, are these works complementary or do they present the same ideas? In my opinion, the current work is deeper, in terms of discussion, especially of the mechanisms. But there is some overlap; I do not believe this invalidates the work, but the authors should pay attention to the similarities.

Regarding the work itself, the English must be improved overall; for example, in the abstract it states: “primary secondary resource,” and the more correct expression would be something like “main secondary source.” Problems of this kind are present throughout the text, and I will try to point out the main ones, but I recommend an English revision. There are many occurrences of plural mistakes and an excessive, in my opinion, use of the first person “we.”

Line 52, the cited paper (11) is really about ITO? Or is it IGZO… please check that.

In the methodology, there is something that concerns me: when using scraps from processes in your own laboratory from different “flawed products,” how do you ensure the homogeneity of the sample? If you cannot guarantee that the material used for electrolysis is always the same, or at least homogeneous, you will be varying two variables at the same time, the composition of the ITO and the parameter you are studying. This needs to be clarified in the text. As it is currently written, it seems that random scraps were cut into cubes and used, but their initial composition and morphology were disregarded. Is the only characterization performed on the initial material the Raman spectroscopy mentioned in the product characterization chapter?

There are other issues related to the characterization. The authors state that the characterization of the cathodic product is performed by ICP. This material needs to be digested for this procedure, since the analysis is carried out by wet chemistry in ICP. Which methodology was used?

In section 2.3 First principles calculations, it seems more like a discussion of phenomena than a methodology. I would be more direct: I would place the method itself in this section and move the discussion to the Results and Discussion section.

In an attempt to summarize a large amount of information in a few figures, the authors combined many figures into a single one, which makes the text uncomfortable to read because the reader must keep going back and forth across many pages between the text and the figure, and it also creates ridiculously long titles; Figure 1, for example, has a 15-line title.

The methodology and the results do not correspond; many experiments and techniques are not clearly described in the methodology. I recommend a complete reorganization of both sections so that each experiment is properly cited.

It is not difficult to find studies in the literature that use $H_2SO_4$ for the solubilization of ITO, which differs from the result shown in Figure 1F. Wouldn’t it be better to carry out this leaching first and, after this process, apply electrolysis? Regenerating the acid in the process and recovering the metals? Is the use of ITO as an electrode really that advantageous? I was not

convinced.

Reviewer #2

(Remarks to the Author)
I co-reviewed this manuscript with one of the reviewers who provided the listed reports. This is part of the Nature Communications initiative to facilitate training in peer review and to provide appropriate recognition for Early Career Researchers who co-review manuscripts.

Reviewer #3

(Remarks to the Author)
1. Figure 1 demonstrates a sharp increase in the dissolution rate of anodic ITO under applied current compared to non-electrified conditions. This intriguing phenomenon underpins the authors' approach to recycling spent ITO. The mentioned electrolysis was conducted in acidic media, would anodic dissolution occur in neutral or alkaline electrolytes? Moreover, would the dissolved metal ions undergo direct precipitation? Such behavior could potentially diversify the recoverable products of this technology, and the authors are encouraged to briefly discuss this possibility.
2. In order to improve the cathodic current efficiency, the authors introduced initial indium ions into the electrolyte to reduce current wastage during the initial electrolysis stage—a strategy strongly supported by the key data in Figure 5d. How does the indium ion concentration evolve throughout the electrolytic process, and to what extent can dynamic equilibrium be maintained? It is recommended that the authors perform time-dependent measurements of the electrolyte's indium ion concentration to elucidate this behavior.
3. The fitted curves in Figures S6 and S7 are intended to further explain the electrochemical behavior of indium and tin ions in electrolytes, but the fitting degree of the curves is not high. Although this outcome does not invalidate the authors' conclusions, it is advisable to repeat the experiments to obtain better-fitted curves to address the minor issue.
4. The anode itself is composed of spent ITO target material, which eliminates the need for heterogeneous anodes and thereby prevents the risk of impurity introduction from foreign materials—a key advantage of this technology. However, as electrolysis progresses, impurities inevitably accumulate in the electrolyte. When the product purity falls below the requirements, electrolyte replacement becomes necessary. How might the spent electrolyte be treated? Is it analogous to the treatment strategies for electrolytes in copper electrorefining?
5. The 100 A scale-up experiment depicted in Figure 6 convincingly validates the technical scalability and establishes foundation for industrial implementation. Regarding a minor issue: Are there specific requirements for the dimensions and geometry of waste ITO targets when used as anodes? and do the spent ITO need to be pre-processed?
6. The gas symbol for the reaction product in Figure 1 has been omitted by the author, and it should be supplemented. The font size in the figures of the manuscript is somewhat small (such as in Figure 3a), and the authors can slightly increase it to improve readability. The author should carefully proofread the text to eliminate grammatical errors.

Reviewer #4

(Remarks to the Author)
Overall Assessment
The manuscript addresses an important topic related to electrochemical recovery of indium from spent ITO materials. However, in its current form, the conclusions are frequently drawn without sufficient mechanistic evidence or experimental validation. In several instances, interpretations rely heavily on speculation rather than direct data or appropriate literature support, which does not meet the level of rigor expected for Nature Communications. In addition, critical experimental details are missing throughout the manuscript, limiting reproducibility and undermining confidence in the reported results. Taken together, the conceptual overstatements, lack of mechanistic substantiation, and insufficient methodological transparency represent serious shortcomings. As a result, I believe, the manuscript, even after revision, would not be suitable for publication in Nature Communications.

Specific Comments
1. The Introduction would benefit from a more balanced and structured comparison between pyrometallurgical and hydrometallurgical recycling routes. While the limitations of conventional pyro- and hydro-metallurgical processes are emphasized, their continued industrial adoption suggests that these routes also possess practical advantages (e.g., robustness, scalability, or tolerance to feedstock variability) that should be acknowledged. A concise discussion of both strengths and weaknesses would establish a more objective baseline for positioning electrochemical strategies. In addition, Line 46 mentions electrochemical treatments integrated with hydrometallurgy; however, the distinction between previously reported electrochemical approaches and the specific electrochemical strategy employed in this work remains unclear. Given that the present study is fundamentally electrochemical in nature, the authors should explicitly clarify how their approach differs from, or advances beyond, existing electrochemical recycling and recovery methods. Expanding the discussion of prior electrochemical metal recovery studies that exploit cathodic or combined anodic–cathodic reactions for high-purity metal recovery would significantly strengthen the Introduction (e.g., The Scientific World Journal, 2015, 494015; Energy Storage Materials, 2025, 82, 104646).

2. An important unresolved issue concerns whether the observed deposits correspond to simple codeposition or true alloy formation. Considering that the In-O bond is weaker than the Sn-O bond, preferential formation of In-rich phases rather than homogeneous alloys would be expected during electrolysis. Quantitative ICP analysis of In and Sn in the deposits should be provided to substantiate claims of alloy formation. In addition, high-resolution TEM and STEM analyses are necessary to

clarify the phase structure and elemental distribution at the nanoscale.

3. The observed metal release behavior may partially overlap with chloride-induced corrosion processes rather than being purely electrochemical in origin. Additional characterization is required to distinguish electrochemical dissolution from chemical corrosion, particularly under Cl2-evolving or chloride-mediated conditions. Further investigation into the role of chlorine species is therefore necessary.

4. There is a fundamental terminological inconsistency regarding voltage and potential. In Line 146, the authors refer to “voltage exceeding 1.5 V,” whereas Fig. 1a presents the y-axis as potential (V vs $O_2/O_2^-$). Voltage and potential are not interchangeable terms and must be clearly distinguished. It should be clarified whether the authors are referring to the cell voltage in a two-electrode configuration or the electrode potential referenced to a specific redox couple.

5. In Lines 148–149, the statement that “with decrease in pH metallic indium and tin demonstrate notable regional advantage” is unclear and insufficiently explained. The meaning of “regional advantage” is ambiguous and should be clarified with a more precise electrochemical or physicochemical interpretation.

6. In Line 154, the authors attribute the observed current fluctuation at the initial stage to electrochemical dissolution of s-ITO. However, this conclusion appears to be drawn solely from current responses without direct supporting evidence. Could these fluctuations instead arise from early-stage surface adsorption, restructuring, or transient interfacial processes? This interpretation requires further justification or additional supporting data.

7. In Fig. 2, conclusions are drawn primarily from XPS analysis. Given the highly surface-sensitive nature of XPS, it is difficult to draw representative conclusions about bulk composition or phase evolution. Depth-profile XPS or complementary bulk-sensitive characterization should be provided to support the interpretations.

8. In several instances, conclusions are presented with insufficient caution. For example, in Line 375, the statement that “this shift is primarily attributed to the shield effect” is made without clear experimental or theoretical evidence. Such claims should be either substantiated with data or revised to reflect a more tentative interpretation.

9. In Line 386, the authors refer to “the square root of the peak current density,” whereas the classical electrochemical relationship involves the square root of the scan rate and the peak current itself. This statement appears to be incorrect and should be carefully revised.

10. The experimental details provided for Section 3.4 are severely insufficient. While photographs of electrodes are included in the Supporting Information, essential information such as cell configuration, electrode dimensions, cell geometry, agitation or stirring conditions, and electrolyte flow rate is missing. In particular, the rationale for selecting a current density of 0.072 A cm-2 is not provided. The authors should also clarify why potentiostatic operation was chosen instead of galvanostatic control and provide a sound theoretical justification for this choice.

11. According to Lines 460-470, indium-only recovery is achieved in the absence of NaCl, whereas In–Sn alloy formation occurs when NaCl is added (Fig. S10). However, this observation appears inconsistent with the CV data shown in Fig. S5 and Fig. 3e, where indium deposition is predicted to be suppressed without NaCl, while tin deposition should remain feasible. The discrepancy between electrochemical predictions and experimental outcomes must be carefully addressed.

12. The Supporting Information also lacks sufficient detail. For example, in the calculation of current efficiency, the number of electrons transferred (z) is mentioned but not explicitly defined. The actual values used in the calculations should be clearly stated to ensure transparency and reproducibility.

13. Although the manuscript focuses primarily on recovery purity, the behavior of the system under prolonged electrodeposition aimed at achieving high recovery yields is not discussed. How does extended operation affect selectivity, recovery efficiency, and solution pH? These aspects are essential for evaluating the practical viability of the process and should be addressed.

14. In Fig. S17, reusability is assessed mainly from the perspective of impurity accumulation. However, a more holistic evaluation is required, including recovery efficiency, recovery purity, and current efficiency over multiple cycles. Without such data, the claim of process reusability remains insufficiently supported.

15. The authors compare the reported energy consumption primarily with pyro- and hydrometallurgical processes, which does not reflect the current state of the art. A more meaningful comparison would be with acid-leaching-based hydrometallurgical routes. For example, the statement “Through systematic optimization of electrolysis parameters, this method achieved a cathode current efficiency of 71.12% and an energy consumption of 4.87 kWh $kg^{-1}$ in hundred-ampere-scale trials” lacks proper context without benchmarking against comparable electrochemical or hydrometallurgical processes.

16. During s-ITO leaching, experiments were conducted using 5.52 M $H_2SO_4$ and 1–1.2 M NaCl (approximately a 5:1 ratio, Fig. 5). However, the analyses presented in Fig. 3 were performed under different electrolyte compositions, where the $H_2SO_4$-to-NaCl ratio is 1:1 and overall concentrations also differ. It is therefore unclear whether the conclusions drawn from Fig. 3 are directly applicable to the actual s-ITO leaching conditions. Clarification is required as to whether similar trends would be observed under electrolyte compositions identical to those used in the leaching experiments.

17. Related to the previous point, the manuscript should explicitly demonstrate whether the mechanistic interpretations derived from simplified electrolyte systems remain valid under the highly concentrated acid and chloride conditions employed for practical s-ITO leaching. Without such validation, extrapolation of the conclusions to realistic operating conditions remains questionable.

Version 1:

Reviewer comments:

Reviewer #1

(Remarks to the Author)
First, we would like to congratulate the authors on the hard work invested in improving the form and structure of the manuscript. In our opinion, the document is now much clearer to read. The results are well explained, and the methodology is solid.

From our side, we consider the revision complete. It is now up to the editor to review the final version and decide on the relevance of the topic for the journal. In our view, this is a well-executed piece of work.

Reviewer #2

(Remarks to the Author)
I co-reviewed this manuscript with one of the reviewers who provided the listed reports. This is part of the Nature Communications initiative to facilitate training in peer review and to provide appropriate recognition for Early Career Researchers who co-review manuscripts.

Reviewer #3

(Remarks to the Author)
After reviewing the comments provided by the authors, I think the reviesed manuscript can be accepted for publication in this journal.

Reviewer #4

(Remarks to the Author)
I have reviewed the revised version of manuscript and find that the authors have satisfactorily addressed the points raised in the previous review, and in my opinion the paper is now acceptable for publication.

**Open Access** This Peer Review File is licensed under a Creative Commons Attribution 4.0 International License, which

permits use, sharing, adaptation, distribution and reproduction in any medium or format, as long as you give appropriate credit to the original author(s) and the source, provide a link to the Creative Commons license, and indicate if changes were made.
In cases where reviewers are anonymous, credit should be given to 'Anonymous Referee' and the source.
The images or other third party material in this Peer Review File are included in the article’s Creative Commons license, unless indicated otherwise in a credit line to the material. If material is not included in the article’s Creative Commons license and your intended use is not permitted by statutory regulation or exceeds the permitted use, you will need to obtain permission directly from the copyright holder.
To view a copy of this license, visit https://creativecommons.org/licenses/by/4.0/

## Point-by-point response to the reviewers' comments

Dear Editor and Reviewers:

We thank the editor and reviewers' efforts on our manuscript entitled "**Electrochemical upcycling of n-type spent ITO via charge-induced deconstruction**" **(Manuscript ID: NCOMMS-25-92325)**. These valuable suggestions have enabled us to revise the manuscript more rigorously, improve our understanding of the content, and deepen our analysis. All modifications made in response to the reviewers' comments have been highlighted in blue in this response letter and the revised manuscript.

Once again, thank you very much for your comments and suggestions.

With best regards,

Sincerely,

Jianxun Song & Shaolong Li

==============================================================

**Reviewer #1:**

First, I would like to congratulate the authors on the extensive and good work. The topic is relevant, and the use of electrochemistry to improve and enable leaching processes is an interesting approach.

**Discussion:**

We greatly appreciate your kind feedback and recognition of our work. The manuscript has been revised according to your suggestions.

**Issue 1:**

The last author, Jianxun Song, has similar publications in the area. Like the recently published article: https://doi.org/10.1016/j.susmat.2025.e01710. A first question is, are these works complementary or do they present the same ideas? In my opinion, the current work is deeper, in terms of discussion, especially of the mechanisms. But there is some overlap; I do not believe this invalidates the work, but the authors should pay attention to the similarities.

**Discussion:**

We sincerely thank the reviewers for bringing this important point to our attention. We have carefully reviewed the recent paper by Song et al. published in *Sustainable Materials and*

*Technologies* (DOI: https://doi.org/10.1016/j.susmat.2025.e01710) and appreciate the opportunity to clarify the relationship between our present work and this publication.

Although both studies fall within the same general research field, they differ significantly and fundamentally. The above-mentioned work only observed the promoting effect of current on the dissolution of s-ITO anodes, without further mechanistic investigation provided. As the reviewers astutely pointed out, our current manuscript presents a substantial and in-depth investigation of the underlying mechanism. Our study combines first-principles calculations with advanced characterization techniques, including electron paramagnetic resonance (EPR), ultraviolet photoelectron spectroscopy (UPS), and nuclear magnetic resonance (NMR) spectroscopy. Through the synergistic integration of experimental characterizations and theoretical calculations, we provide a far more comprehensive and in-depth understanding of the dissolution and recovery processes of s-ITO anodes, thereby offering mechanistic insights well beyond the scope of the earlier publication. Furthermore, we explore the synergistic mechanism of anodic and cathodic efficiencies, perform scaled-up recovery based on these findings, and conduct a comprehensive evaluation of the technology.

In summary, the present study focuses on elucidating the anodic dissolution mechanism and evaluating the comprehensive technical performance, providing a new pathway for the recycling of other semiconductor materials—all of which extend far beyond the scope of the aforementioned paper. We have cited and briefly described this relevant work in the revised manuscript.

**The related revision in the main text:**

**Lines 63-68 in Introduction Section:**

Preliminary studies in our group demonstrate that direct electrolysis with s-ITO as the anode in aqueous solution results in pronounced electrochemical dissolution, facilitating the release of metal ions into the electrolyte[18]. Driven by this observation and the limitations of traditional recycling approaches, we herein propose an electrochemical recycling strategy based on the charge-induced deconstruction of s-ITO.

18. Lv, X. *et al.* Upcycling waste ITO powders through integrated leaching enhancement and selective deposition. *Sustain. Mater. Technol.* **46**, e01710 (2025).

**Issue 2:**

Regarding the work itself, the English must be improved overall; for example, in the abstract it states: “primary secondary resource,” and the more correct expression would be something like “main secondary source.” Problems of this kind are present throughout the text, and I will try to point out the main ones, but I recommend an English revision. There are many occurrences of plural mistakes and an excessive, in my opinion, use of the first person “we.”

**Discussion:**

We sincerely thank the reviewer for this constructive feedback regarding the language quality of our manuscript. We fully acknowledge the validity of these concerns and have taken comprehensive measures to address them.

**English Language Revision:** Following the reviewer's recommendation, the entire manuscript has undergone professional English editing by a native-speaking scientific editor with expertise in materials science and electrochemistry. This has ensured that the language is not only grammatically correct but also conforms to the conventions of scientific writing in our discipline.

**Specific Corrections:** We have carefully revised the title, abstract and throughout the text to eliminate awkward phrasing. For instance, "primary secondary resource" has been corrected to "main secondary source" as suggested. We have systematically identified and corrected all instances of pluralization errors and other grammatical inconsistencies.

**Reduction of First-Person Usage:** We recognize the reviewer's observation regarding the excessive use of "we" and have substantially revised the manuscript to minimize first-person pronouns. Wherever appropriate, we have adopted passive voice constructions or impersonal expressions (e.g., "it was observed that," "the results indicate," "this study demonstrates") to maintain objectivity and align with standard scientific writing conventions. First-person usage has been retained only in specific instances where it is conventional or necessary for clarity, such as in the description of our research objectives or hypotheses.

We believe these revisions have significantly enhanced the readability and professional quality of the manuscript. We are grateful to the reviewer for highlighting these important issues, which have undoubtedly strengthened the presentation of our work.

**The representative revision in the main text:**

**Lines 1-2 in Manuscript Title:**

Electrochemical upcycling of spent ITO via charge-induced deconstruction

**Lines 15-16 in Abstract Section:**

Spent ITO (s-ITO, the mass ratio of $In_2O_3$ to $SnO_2$ is approximately 9:1) represents the main secondary resource for the rare metal indium

**Lines 69-75 Introduction Section:**

In this route, a $H_2SO_4$-based system is selected as the electrolyte, with a certain concentration of NaCl incorporated. S-ITO is directly utilized as anode, and In-Sn alloy is deposited on the titanium cathode plate. Under charge excitation, the reactivity of oxygen on the s-ITO anode surface is activated, thereby weakening the interaction between metal and oxygen ions. Indium and tin ions are released into the electrolyte, migrate to the cathode, and are reduced. The addition of NaCl to the electrolyte can further promote anodic dissolution and cathodic deposition.

**Lines 185-186 in Results Section:**

Electron paramagnetic resonance (EPR) spectroscopy was employed to analyze the changes in the number of defects in s-ITO before and after electrolysis.

**Lines 196-199 in Results Section:**

First-principles calculations were performed to elucidate the intrinsic mechanism by which oxygen vacancies promote the anodic deconstruction of s-ITO (The lattice optimization of the model (**Figure S5**) and partial energy calculations (**Figure S6**) are provided in **Section 2** of **Supplementary Information**).

**Lines 285 in Results Section:**

LSV was employed to investigate the effect of $H_2SO_4$ concentration on HER.

**Lines 319-320 in Results Section:**

Based on the aforementioned research, the reaction mechanism of s-ITO during electrolysis can be summarized, as illustrated in **Figure 5**.

Given extensive linguistic revisions throughout the manuscript, the above only exemplify representative modifications; comprehensive revisions are detailed in the revised version of main text.

**Issue 3:**

Line 52, the cited paper (11) is really about ITO? Or is it IGZO… please check that.

**Discussion:**

Thank you for your careful review. Reference 11 indeed describes our group's work on the electrochemical recycling of IGZO. Due to our negligence, the citation was inaccurate, and we have replaced the reference accordingly.

**The related revision in the main text:**

**Lines 56-63 in Introduction Section:**

A typical representative of such electrolysis methods is the molten salt electro-deoxidation technology[15]. Our group has conducted extensive research on the recycling of s-ITO via electro-deoxidation. The utilization of s-ITO as the cathode during electrolysis in $CaCl_2$-based molten salt allows the direct production of In-Sn alloy on the cathode. However, the depolarization effect of the liquid alloy formed after electro-deoxidation can lead to the underpotential deposition of metallic calcium on the cathode product. Furthermore, the employment of carbon anodes generates carbon oxide gases, and the anode materials increases the risk of elevated impurities in the product[16,17].

15. Su, X. *et al.* Recovery and reuse of spent ITO targets through electrochemical techniques. *J. Electrochem. Soc.* **169**, 023507 (2022).

16. Su, X., Li, S., Liu, C., He, J. & Song, J. Reduction mechanism of spent ITO target in $CaCl_2$ molten salt. *Sep. Purif. Technol.* **299**, 121778 (2022).

17. Song, J., Li, S., Lv, Z. & He, J. In-Sn alloy extraction through novel process for recycling of spent ITO targets. *Sustain. Mater. Technol.* **41**, e01027 (2024).

**Issue 4:**

In the methodology, there is something that concerns me: when using scraps from processes in your own laboratory from different "flawed products," how do you ensure the homogeneity of the sample? If you cannot guarantee that the material used for electrolysis is always the same, or at least homogeneous, you will be varying two variables at the same time, the composition of the ITO and the parameter you are studying. This needs to be clarified in the text. As it is currently written, it seems that random scraps were cut into cubes and used, but their initial

composition and morphology were disregarded. Is the only characterization performed on the initial material the Raman spectroscopy mentioned in the product characterization chapter?

**Discussion:**

Thank you for your valuable comments. Regarding the issues you raised concerning material composition and structural uniformity, we would like to explain from the following aspects: First of all, as mentioned in the abstract, ITO targets are mainly used in the liquid crystal display (LCD) field, where the mass ratio of indium oxide ($In_2O_3$) to tin oxide ($SnO_2$) is approximately 9:1[1–3]. In other application fields, this ratio may fluctuate slightly but generally remains around 9:1. It is worth noting that processes such as sintering, machining, and sputtering do not have a significant impact on the ratio of the two main metals, which ensures the relative stability of the indium-tin ratio in spent ITO (s-ITO). In addition, the preparation of ITO targets requires high raw material purity (3N, even 4N or higher)[4,5]. Therefore, the impurity content in the generated s-ITO is relatively low, which barely affects the uniformity of their composition and microstructure. Our characterization results of the raw materials (including XRD, Raman, UPS, EPR etc.) further confirm that the elements are uniformly distributed and the phase is single. From the reaction mechanism we proposed, the dissolution of ITO originates from the disturbance of charges and the promotion of oxygen vacancy formation. Even slight fluctuations in raw material composition will not change the mechanism of charge action. Based on the above reasons, we did not specifically study the impact of raw material composition on the electrolysis recovery effect.

**The related figures in the main text:**

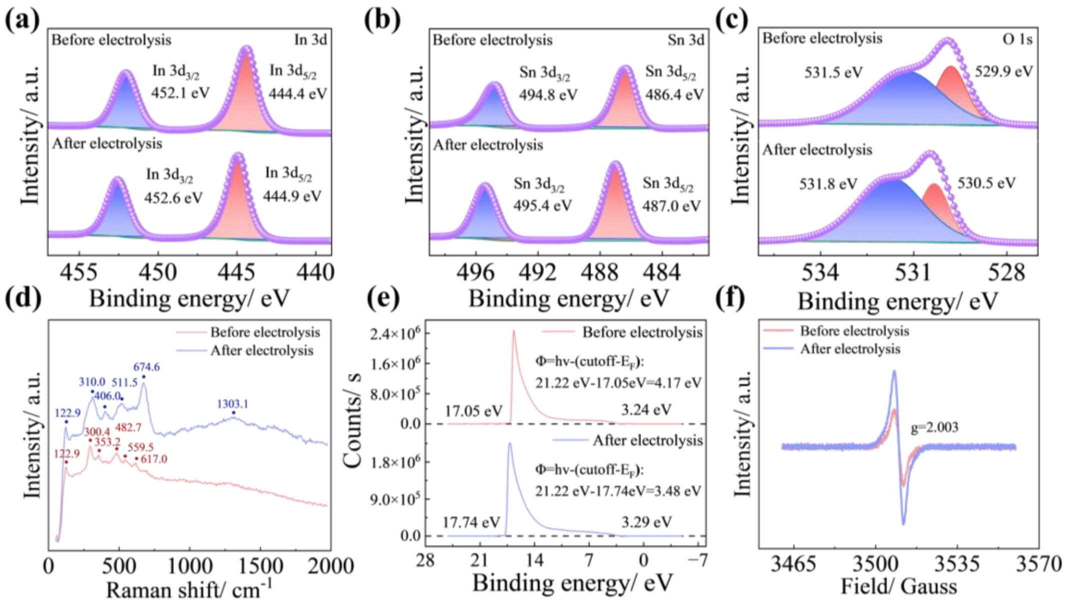


**Figure 2**

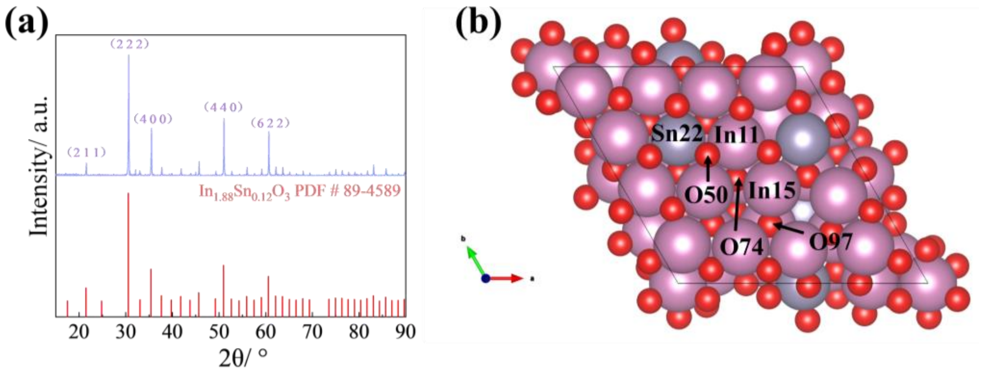


**Figure S6**

## Support references:

S1. Zervos, M. *et al.* Broad compositional tunability of indium tin oxide nanowires grown by the vapor- liquid-solid mechanism. *APL Mater.* **2**, 056104 (2014).

S2. Wang, N., Yang, Y. H., Chen, J., Xu, N. & Yang, G. One-dimensional Zn-doped $In_2O_3$ −$SnO_2$ superlattice nanostructures. *J. Phys. Chem. C* **114**, 2909–2912 (2010).

S3. Oh, S. J., Kwon, J. H., Lee, S., Choi, K. C. & Kim, T.-S. Unveiling the annealing-dependent mechanical properties of freestanding indium tin oxide thin films. *ACS Appl. Mater. Interfaces* **13**, 16650–16659 (2021).

S4. Su, Z., Wang, Y., Zhang, Y. & Jiang, T. A novel method for synchronous separation of In/Sn from waste indium tin oxide (ITO) target: In-situ regeneration of nano-ITO powder. *Separation and Purification Technology* **310**, 123104 (2023).

S5. Wada, Y. *et al.* Sputtering deposition of dense and low-resistive amorphous $In_2O_3$: Sn films

under ZONE-T conditions of Thornton's structural diagram. *Appl. Phys. Lett.* **124**, 242101 (2024).

**Issue 5:**

There are other issues related to the characterization. The authors state that the characterization of the cathodic product is performed by ICP. This material needs to be digested for this procedure, since the analysis is carried out by wet chemistry in ICP. Which methodology was used?

**Discussion:**

We sincerely thank the reviewer for this important clarification regarding the cathodic product characterization.

The cathode products were subjected to acid digestion prior to ICP-MS analysis according to the following standardized procedure. The sample was accurately weighed using an analytical balance, moistened with a minimal volume of deionized water, and subsequently transferred to a polytetrafluoroethylene (PTFE) digestion vessel. A digestion mixture comprising 9 mL of aqua regia (a 3: 1 volumetric mixture of concentrated HCl and $HNO_3$) and 1 mL of hydrofluoric acid (HF) was carefully added. The vessel was sealed and subjected to a programmed microwave digestion protocol: pre-digestion at 120 °C for 30 min, followed by sequential temperature ramping—130 °C (reached within 5 min, held for 3 min), 150 °C (reached within 3 min, held for 10 min), and 180 °C (reached within 3 min, held for 30 min). Upon cooling to 60 °C, the digested solution was quantitatively transferred and diluted to 25 mL with deionized water, homogenized, and prepared for subsequent analysis. An appropriate aliquot was subsequently diluted to fall within the calibrated linear range of the ICP-MS instrument (Agilent 7800 Series). Matrix effects and instrumental drift were corrected using an internal standard, with the instrument operating in ICP-MS mode under the following optimized parameters: pump rate of 20 r/min, nebulizer gas flow of 1.00 L/min, auxiliary gas flow of 1.00 L/min, sample flush time of 40 s, and RF power of 1550 W.

The elemental concentrations were calculated according to the following equations:

$$C_1=C_0 f$$

$$C_x=C_1 V_0/V$$

where $V$ represents the volume (mL) of the sample aliquot taken for analysis, as accurately measured by pipette; $V_0$ denotes the final constant volume (mL) of the digested sample solution; $f$ is the dilution factor; $C_0$ indicates the elemental concentration (μg/L) in the test solution as determined by the instrument; $C_1$ represents the elemental concentration (μg/L) in the stock digestion solution; and $C_x$ is the final calculated concentration (μg/L) of the target element. Triplicate digestions were conducted for each sample to ensure reproducibility, with relative standard deviations below 2%.

We have incorporated the key information of this digestion method into the Method Section of the revised manuscript to ensure the full transparency and reproducibility of our analytical procedure.

**The related revision in the main text:**

**Lines 621-627 in Methods Section:**

**Inductively coupled plasma mass spectrometry** (ICP-MS, Agilent 7800 Series) was used to determine the concentrations of impurity ions in the raw s-ITO, the recycled electrolyte, and the cathode product. For solid samples, acid digestion was conducted prior to ICP-MS analysis. The digestion solution was a mixture composed of 9 mL of aqua regia and 1 mL of hydrofluoric acid. Subsequently, the vessel was sealed, microwave digestion was carried out, and finally, the solution was diluted to within the calibrated linear range of the ICP-MS instrument for measurement.

**Issue 6:**

In section 2.3 First principles calculations, it seems more like a discussion of phenomena than a methodology. I would be more direct: I would place the method itself in this section and move the discussion to the Results and Discussion section.

**Discussion:**

We sincerely appreciate your constructive suggestion on the organization of Section 2.3 (First-principles calculations).

We agree that the methodology should be clearly and concisely presented in the Methods section, with interpretative discussion relocated to the Results and Discussion section. Accordingly, we have revised the manuscript accordingly: the computational details and

methodological procedures are now concentrated and clearly described in **Methods Section**, and all interpretative and phenomenological discussion has been moved to the appropriate parts of the Results and Discussion section to improve the logical structure and readability of the manuscript. Specifically, this section has been relocated to the Supplementary Information.

**The related revision in the Supplementary Information:**

**Lines 11-45: Section 2: Lattice optimization and energy calculation of the basic model**

$In_2O_3$ has a cubic structure and belongs to the point group *Ia3*[1], with the unit cell encompassing 80 atoms, equivalent to 16 $In_2O_3$ molecules. Indium ions occupy two different coordinate positions in the lattice, called "8b" and "24d" positions, while the position of oxygen ions is called "48e", as shown in **Figure S5**. After geometric structure optimization, the lattice constants of $In_2O_3$ doped with different Sn were obtained, the lattice constant of $In_2O_3$ is 10.202 Å, which is close to the experimental value of 10.118 Å with an error of less than 1%, indicating the reliability of the calculated results[2]. After Sn doping, the lattice constant slightly increases, but remains between 10.2 Å and 10.3 Å. The reason is that Sn doping generates residual stress in the lattice, leading to lattice distortion, and $Sn^{4+}$ contributes one more free electron than $In^{3+}$, which enhances the repulsion between excess positive charges and increases the total energy of the system, resulting in an increase in volume[3]. Upon achieving structural convergence, the band gap ($E_g$) of $In_2O_3$ was determined to be 2.594 eV (**Figure S6a**), which is consistent with the results reported in the literature. The total density of states (TDOS) and partial density of states (PDOS) were calculated to understand the band structure of $In_2O_3$ and the contribution of each atomic orbital. The calculation results in **Figure S6b** indicate that the top of the valence band in $In_2O_3$ is mainly contributed by the O 2p and In 4d states, while the valence band at the low-energy end is primarily derived from the interaction between the O 2s, In 5s, and In 5p orbitals. The bottom of the conduction band is mainly attributed to the contribution of the In 5s orbitals. However, when Sn is doped at a concentration of 9.375 at.% to replace In at the 8b site, $E_g$ of ITO decreases to 2.245 eV, as shown in **Figure S6c**. The band structure shifts toward lower energy levels, and the Fermi level ($E_F$) moves toward the conduction band, exhibiting characteristics of metallic conductivity. Thus, the $E_g$ of ITO decreases, accompanied by an increase in electrical conductivity. After Sn doping, the density of states (DOS) shifts toward lower energy, and $E_F$ enters the conduction band, which is consistent with the band structure

results shown in **Figure S6d**. Notably, electrons in the In 5s and Sn 5s orbitals mainly occupy the bottom of the conduction band, which explains the enhanced electrical properties of ITO after Sn doping. Since $Sn^{4+}$ donates one additional free electron compared to $In^{3+}$, Sn doping increases the number of free carriers. The donor levels formed near $E_F$ degenerate with the conduction band, forming a so-called degenerate semiconductor and enhancing electrical conductivity. This observation is consistent with the intrinsic reduction in the $E_g$ of ITO after Sn doping, as revealed by the band structure analysis. The model with a Sn doping concentration of 9.375 at.% matches the mass ratio of $In_2O_3$ to $SnO_2$ (9:1) in ITO; therefore, this doping concentration was selected for the construction of the ITO model and subsequent calculations.

1. Ziemba, M., Schumacher, L. & Hess, C. Reduction behavior of cubic $In_2O_3$ nanoparticles by combined multiple *In Situ* spectroscopy and DFT. *J. Phys. Chem. Lett.* **12**, 3749–3754 (2021).
2. Song, L. *et al.* Sr-doped cubic $In_2O_3$ /rhombohedral $In_2O_3$ homojunction nanowires for highly sensitive and selective breath ethanol sensing: Experiment and DFT simulation studies. *ACS Appl. Mater. Interfaces* **12**, 1270–1279 (2020).
3. Schimpf, A. M., Lounis, S. D., Runnerstrom, E. L., Milliron, D. J. & Gamelin, D. R. Redox Chemistries and plasmon energies of photodoped $In_2O_3$ and Sn-doped $In_2O_3$ (ITO) nanocrystals. *J. Am. Chem. Soc.* **137**, 518–524 (2015).

**Issue 7:**

In an attempt to summarize a large amount of information in a few figures, the authors combined many figures into a single one, which makes the text uncomfortable to read because the reader must keep going back and forth across many pages between the text and the figure, and it also creates ridiculously long titles; Figure 1, for example, has a 15-line title.

**Discussion:**

We greatly appreciate your constructive comment. We agree with your view that excessively long figure captions and excessive content within individual panels may indeed compromise readability. The rationale for combining these figures is that each composite figure is organized around a distinct, cohesive theme. For instance, **Figure 1** focuses comprehensively on the feasibility of anodic

dissolution of s-ITO, including thermodynamic calculations, electrochemical characterizations, and preliminary anodic dissolution experiments. Similarly, **Figure 2** is centered on the mechanism of anodic dissolution.

With this in mind, we would respectfully request that individual figures, such as **Figure 2**, rather than all figures, be disassembled and reorganized. Nevertheless, as you have pointed out, the original figure captions were overly lengthy. We have now substantially revised and streamlined all figure captions while preserving their original meaning and key information. In addition, the issue of frequent page-flipping that you mentioned will be resolved through further refinement of the manuscript text and the final typesetting layout.

**The related revision in the main text:**

**Lines 130-137 in Results Section:**

**Figure 1**. Feasibility investigation of charge-promoted anodic dissolution of s-ITO. (a) Theoretical reduction potentials of $In_2O_3$, $SnO_2$ and $H_2O$ (data from HSC 6.0, The potential calculation method is detailed in **Section 1** of **Supplementary Information**); E-pH diagrams of (b) In-$H_2O$ system and (c) Sn-$H_2O$ system (data from HSC 6.0); (d) LSV curve of s-ITO in $H_2SO_4$-NaCl solution (scan rate: 0.1 V $s^{-1}$); Mass loss (e) of s-ITO anode and In and Sn ions concentrations (f) in the electrolyte under different leaching/electrolysis conditions (1: 5.52 mol $L^{-1}$ $H_2SO_4$, 12 h; 2: 5.52 mol $L^{-1}$ $H_2SO_4$ + 1.0 mol $L^{-1}$ NaCl, 12 h; 3: 5.52 mol $L^{-1}$ $H_2SO_4$, 0.072 A $cm^{-2}$, 1.5 h; 4: 5.52 mol $L^{-1}$ $H_2SO_4$ + 1.0 mol $L^{-1}$ NaCl, 0.072 A $cm^{-2}$, 1.5 h).

**Lines 192-195 in Results Section:**

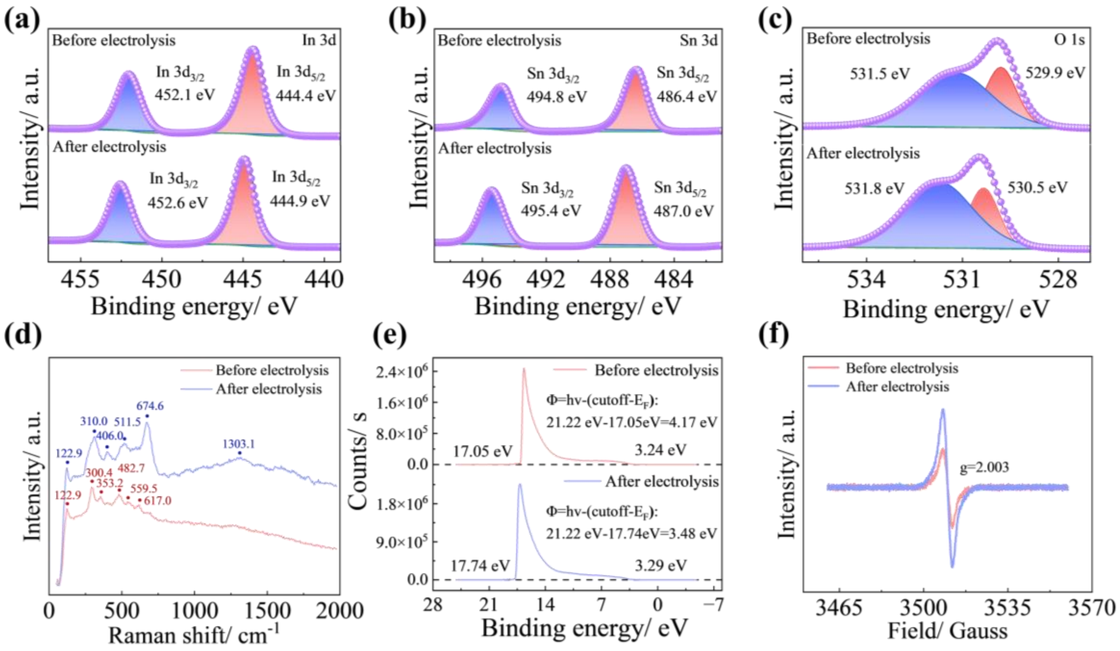


**Figure 2**. Research on anodic deconstruction mechanism. XPS spectra of s-ITO before and after electrolysis: (a) In 3d, (b) Sn 3d, (c) O 1s; Raman spectra (d), UPS spectra (e) and EPR spectra (f) of s-ITO before and after electrolysis.

**Lines 243-249 in Results Section:**

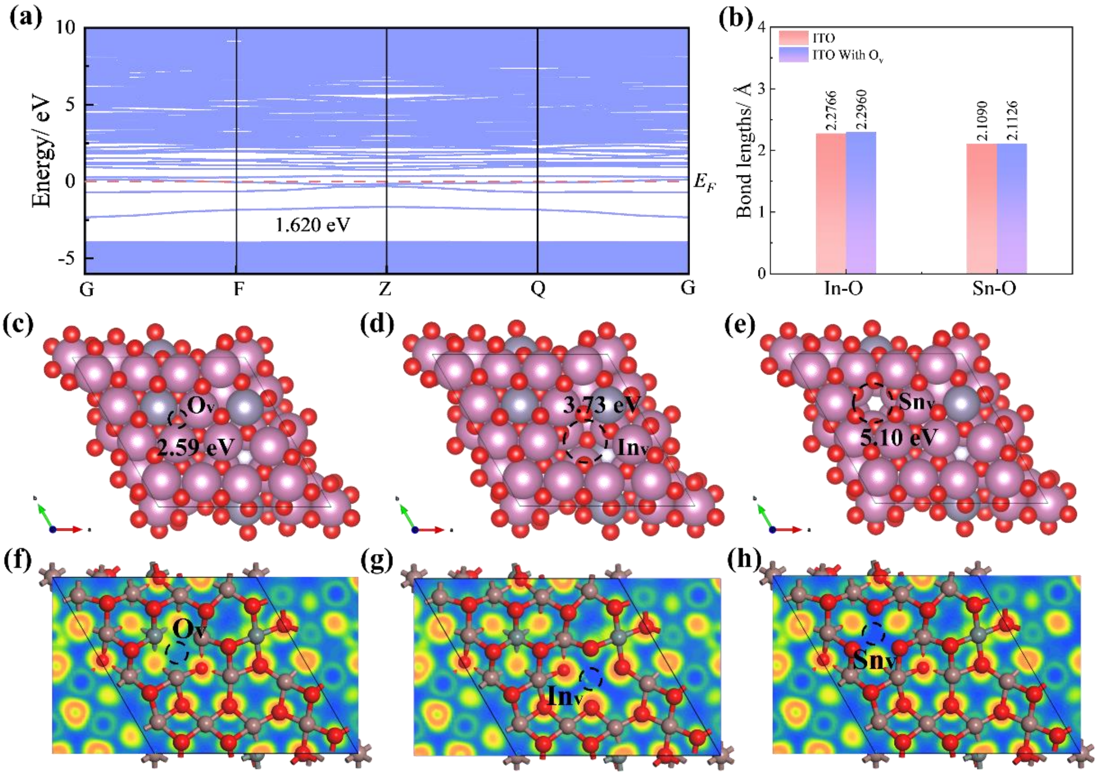


**Figure 3**. Research on the influence mechanism of oxygen vacancies on the crystal structure and electronic structure of ITO. (a) Band structure diagram of ITO ($SnO_2$ doped $In_2O_3$: 9.375

at.%, after removing $O_{50}$); (b) The effect of oxygen vacancies on atomic bond length in ITO; ITO (2 2 2) vacancy model and formation energy: (c) $O_v$ (after removing $O_{50}$), (d) $In_v$ (after removing $In_{15}$), (e) $Sn_v$ (after removing $Sn_{22}$); ELF: (f) $O_v$ (after removing $O_{50}$), (g) $In_v$ (after removing $In_{15}$), (h) $Sn_v$ (after removing $Sn_{22}$).

**Issue 8:**

The methodology and the results do not correspond; many experiments and techniques are not clearly described in the methodology. I recommend a complete reorganization of both sections so that each experiment is properly cited.

**Discussion:**

Thank you for your valuable comments and suggestions.

We have carefully revised and completely reorganized the methods section to ensure that every experiment and technique is described in detail and clearly cited in accordance with the actual content.

**The related revision in the main text:**

**Lines 504-644 in Methods Section:**

**Methods**

**Materials**

S-ITO was derived from machining scraps and sputtering residues produced in our laboratory. In this study, all solutions employed for acid leaching, electrochemical dissolution, and electrolysis recovery were $H_2SO_4$ and $H_2SO_4$-NaCl solutions. $H_2SO_4$ (98%) was purchased from Shanghai Aladdin Biochemical Technology Co., Ltd. NaCl (purity 99.9%), $In_2(SO_4)_3$ (purity 99.99%) and $SnSO_4$ (purity 99%) were purchased from Shanghai McLean Biochemical Technology Co., Ltd.

**Acid leaching and anodic dissolution of s-ITO**

Acid leaching and electrochemical dissolution were employed in $H_2SO_4$ and $H_2SO_4$-NaCl solutions. The concentration of $H_2SO_4$ in the $H_2SO_4$ solution was 5.52 mol $L^{-1}$, while those of $H_2SO_4$ and NaCl in the $H_2SO_4$-NaCl solution were 5.52 mol $L^{-1}$ and 1.0 mol $L^{-1}$, respectively. The anode used for electrochemical dissolution was s-ITO obtained after wire cutting, with a size of approximately 40 mm * 8 mm *5 mm, and the cathode was a high-purity titanium plate

(99.99%) with a size of 100 mm *10 mm * 2 mm, and the current density was 0.072 A $cm^{-2}$. After acid leaching for 12 h and electrolysis for 1.5 h, the mass loss of s-ITO and the concentrations of indium and tin ions in the electrolyte were determined.

### Electrochemical measurement

Electrochemical testing was performed using an Autolab PGSTAT 302N instrument, controlled by the Nova 2.1 software package, within a three-electrode system. In this configuration, a platinum rod served as the auxiliary electrode, while a saturated Ag/AgCl electrode acted as the reference electrode. LSV and Tafel measurements were performed in $H_2SO_4$ (5.52 mol $L^{-1}$) and $H_2SO_4$-NaCl (5.52 mol $L^{-1}$-1.2 mol $L^{-1}$) solutions. For LSV measurements, s-ITO (effective area immersed in electrolyte is 0.08 $cm^2$) and glassy carbon (D = 2 mm, effective area immersed in electrolyte is 0.0314 $cm^2$) were used as the working electrodes at a scan rate of 0.1 V $s^{-1}$. For Tafel measurements, s-ITO (effective area immersed in electrolyte is 0.08 $cm^2$) was employed as the working electrode at a scan rate of 0.02 V $s^{-1}$. CV and SWV were conducted in the same solutions with the addition of 0.01 mol $L^{-1}$ $In^{3+}$ and 0.01 mol $L^{-1}$ $Sn^{2+}$, using a Ti plate (effective area immersed in electrolyte is 0.1 $cm^2$) as the working electrode. The scan rates for CV were 0.02 V $s^{-1}$, 0.04 V $s^{-1}$, 0.06 V $s^{-1}$ and 0.08 V $s^{-1}$, and the frequency for SWV was 2 Hz.

### Electrolysis recovery of s-ITO

Exploration of electrolysis parameters

The anode employed here was s-ITO obtained after wire cutting, with dimensions of approximately 40 mm * 8 mm * 5 mm. The cathode was a high-purity titanium plate (99.99%) with dimensions of 100 mm * 10 mm * 2 mm. The electrolysis cell employed was a cylindrical glass reactor with a maximum working volume of 100 mL. The interelectrode distance between the anode and cathode was fixed at 2.0 cm, and no stirring was applied throughout the electrolysis process. A DC power supply (model UTP1310-II) was used, with a voltage control range of 0-64 V and a current control range of 0-16 A.

Effect of $H_2SO_4$ concentration: Electrolysis was carried out at 0.116 A $cm^{-2}$ for 1.5 h without initial $In^{3+}$ addition, at a fixed NaCl concentration of 1.2 mol $L^{-1}$. The concentrations of $H_2SO_4$ were 3.68 mol $L^{-1}$, 4.60 mol $L^{-1}$, 5.52 mol $L^{-1}$, and 6.44 mol $L^{-1}$, respectively.

Effect of NaCl concentration: Electrolysis was performed at 0.116 A $cm^{-2}$ for 1.5 h without

initial $In^{3+}$ addition, at a fixed $H_2SO_4$ concentration of 5.52 mol $L^{-1}$. The concentrations of NaCl were 0.86 mol $L^{-1}$, 1.03 mol $L^{-1}$, 1.20 mol $L^{-1}$, 1.37 mol $L^{-1}$ and 1.53 mol $L^{-1}$, respectively.

Effect of current density: Electrolysis was conducted for 1.5 h without initial $In^{3+}$ addition, at fixed concentrations of 5.52 mol $L^{-1}$ $H_2SO_4$ and 1.2 mol $L^{-1}$ NaCl. The current densities were set to 0.070 A $cm^{-2}$, 0.093 A $cm^{-2}$, 0.116 A $cm^{-2}$, 0.140 A $cm^{-2}$ and 0.163 A $cm^{-2}$, respectively.

Effect of initial $In^{3+}$ concentration: Electrolysis was carried out for 1.5 h at 0.116 A $cm^{-2}$, at fixed concentrations of 5.52 mol $L^{-1}$ $H_2SO_4$ and 1.2 mol $L^{-1}$ NaCl, with different initial $In^{3+}$ concentrations added. The concentrations of $In^{3+}$ were 0.1 mol $L^{-1}$, 0.2 mol $L^{-1}$ and 0.3 mol $L^{-1}$, respectively.

After electrolysis, the cathodic and anodic current efficiencies were calculated. The detailed calculation method is provided in **Section 3** of **Supplementary Information**.

**Expanded electrolysis recovery of s-ITO**

Expanded electrolysis was conducted in an electrolyte containing 5.52 mol $L^{-1}$ $H_2SO_4$ and 1.2 mol $L^{-1}$ NaCl. A constant current density of 0.116 A $cm^{-2}$ (current is 100 A) was applied. A DC power supply (AGCO Cyber, PDC0317SH) was employed, with a voltage regulation range of 0-30 V and a current regulation range of 0-170 A. The anode was a s-ITO plate (mass: 3.62 kg; dimensions: 400 * 200 * 5 mm), and the cathode was a titanium plate with the same dimensions (**Figure S21**). The s-ITO anode was pre-electrolyzed at 100 A for 2 h to ensure sufficient ion accumulation in the electrolyte. Subsequently, a cathode of identical dimensions was installed, and electrolysis was conducted for 1 h.

After electrolysis, the current efficiency and energy consumption were calculated. The detailed calculation method is provided in **Section 4** of **Supplementary Information**.

**Characterization**

**X-ray photoelectron spectroscopy** (XPS, Thermo Fisher Scientific K-Alpha) was used to investigate the valence state changes of In, Sn, and O on the surface and profile of s-ITO before and after electrolysis.

For surface analysis of s-ITO before and after electrolysis: S-ITO was cleaned with deionized water and ethanol and then dried. S-ITO was electrolyzed in $H_2SO_4$-NaCl solution (5.52 mol $L^{-1}$ $H_2SO_4$ and 1.2 mol $L^{-1}$ NaCl) at a current density of 0.116 A $cm^{-2}$ for 1.5 h. After electrolysis, the s-ITO was rinsed with deionized water and dried

For profile analysis of s-ITO after electrolysis: S-ITO was electrolyzed in $H_2SO_4$-NaCl solution (5.52 mol $L^{-1}$ $H_2SO_4$ and 1.2 mol $L^{-1}$ NaCl) at a current density of 0.116 A $cm^{-2}$ for 1.5 h. After electrolysis, the s-ITO was rinsed with deionized water. The surface of the reacted s-ITO was polished with 2000-mesh sandpaper until the unreacted internal region was exposed, yielding a profile with the reaction zone at the edge and the unreacted zone in the inner part. Subsequently, the profile was cleaned with deionized water and ethanol, followed by drying. XPS analysis was performed using a laser spot with a size of 200 μm, and the analyzed regions were the reacted edge and the unreacted central region.

**Raman spectroscopy** (Horiba Aplora Plus confocal Raman spectrometer) with a 532 nm laser was employed to investigate surface change of s-ITO before and after electrolysis. S-ITO was used as the anode and electrolyzed in $H_2SO_4$-NaCl solution (5.52 mol $L^{-1}$ $H_2SO_4$ and 1.2 mol $L^{-1}$ NaCl) at a current density of 0.116 A $cm^{-2}$ for 1.5 h. After electrolysis, the s-ITO anode was rinsed with deionized water and dried prior to characterization.

**Ultraviolet photoelectron spectroscopy** (UPS, Thermo Fisher Scientific Escalab, USA) was utilized to measure the work function, employing an Ar monatomic ion source with hv = 21.21 eV as the cleaning ion source. The pristine s-ITO was cleaned with deionized water and ethanol before being characterized as the reference sample. S-ITO was used as the anode and electrolyzed in $H_2SO_4$-NaCl solution (5.52 mol $L^{-1}$ $H_2SO_4$ and 1.2 mol $L^{-1}$ NaCl) at a current density of 0.116 A $cm^{-2}$ for 1.5 h. After electrolysis, the s-ITO anode was rinsed with deionized water and dried prior to UPS characterization.

**Electron paramagnetic resonance** (EPR, Bruker Emxano, Germany) was employed to investigate the concentration of oxygen vacancies in s-ITO. The pristine s-ITO was cleaned with deionized water and ethanol for oxygen vacancy characterization. S-ITO was employed as the anode and electrolyzed in $H_2SO_4$-NaCl solution (5.52 mol $L^{-1}$ $H_2SO_4$ and 1.2 mol $L^{-1}$ NaCl) at a current density of 0.116 A $cm^{-2}$ for 1.5 h. After electrolysis, the s-ITO anode was rinsed with deionized water and dried before quantitative analysis of oxygen vacancies.

**Liquid-state nuclear magnetic resonance** (NMR, Bruker 600 MHz, Germany) was employed to detect the change in the $^{17}O$ spectra in different solutions at magnetic field strengths of 400 MHz/500 MHz, using deuterated chloroform as the solvent. All solutions were prepared at room temperature with the same volume, and their compositions were as follows:

5.52 mol $L^{-1}$ $H_2SO_4$ and 0.01 mol $L^{-1}$ indium ions (added as $In_2(SO_4)_3$); 5.52 mol $L^{-1}$ $H_2SO_4$, 0.01 mol $L^{-1}$ $In^{3+}$ (added as $In_2(SO_4)_3$), and 1.2 mol $L^{-1}$ NaCl; 5.52 mol $L^{-1}$ $H_2SO_4$ and 0.01 mol $L^{-1}$ tin ions (added as $SnSO_4$); 5.52 mol $L^{-1}$ $H_2SO_4$, 0.01 mol $L^{-1}$ tin ions (added as $SnSO_4$), and 1.2 mol $L^{-1}$ NaCl.

**Transmission electron microscopy** (TEM, Thermo Fisher Scientific Talos F200X G2, USA) equipped with energy dispersive spectroscopy (EDS) system was used to investigate the cathode product, with an operating voltage of 200 kV. The cathode product was dispersed in ethanol and ultrasonicated for 5 min, followed by sample preparation on a copper grid.

**Scanning Electron Microscopy** (SEM, Zeiss GeminiSEM 300) and the corresponding energy dispersive spectroscopy (EDS) were used to investigate the morphology, microstructure, and element distribution of the cathodic products.

**X-ray diffraction** (XRD, Empyrean) with Cu Kα radiation was employed to identify the phase compositions of the s-ITO and the cathode product.

**Inductively coupled plasma mass spectrometry** (ICP-MS, Agilent 7800 Series) was used to determine the concentrations of impurity ions in the raw s-ITO, the recycled electrolyte, and the cathode product. For solid samples, acid digestion was conducted prior to ICP-MS analysis. The digestion solution was a mixture composed of 9 mL of aqua regia and 1 mL of hydrofluoric acid. Subsequently, the vessel was sealed, microwave digestion was carried out, and finally, the solution was diluted to within the calibrated linear range of the ICP-MS instrument for measurement.

**First-principles calculation**

Perdew Wang (PW91) method[67] in the Generalized Gradient Approximation (GGA) of Density Functional Theory (DFT) is used as the exchange correlation functional, and the pseudopotential is selected as the ultrasoft pseudopotential[68]. Due to $In_2O_3$ being a strongly correlated electronic system, and GGA not fully considering the electronic interactions of strongly correlated electronic systems, whereas the Coulomb potentials of In 4d, Sn 5d, and O 2p electrons were taken into consideration. The Hubbard U values of In 4d, O 2p and Sn 5d electrons are taken as 2 eV, 5 eV and 5 eV, respectively[69,70]. In the geometric structure optimization, the plane wave truncation energy is set to 380 eV, and the Brillouin zone k-point is taken as 2 * 2 * 2[71]. After 100 iterations, the energy convergence range can be ensured to be

less than 1.0 * $10^{-5}$ eV $atom^{-1}$, and the force convergence of each atom is less than 0.03 eV $Å^{-1}$.

**Calculation of Ionic Complex Structure**

Visual MINTEQ 3.1 software was employed to investigate the ionic complex structure. The ion concentration ratio was adjusted according to the optimal process conditions, with the $H_2SO_4$ concentration of 0.00552 mol $L^{-1}$, NaCl concentration of 0.0012 mol $L^{-1}$, In ion concentration of 0.0001 mol $L^{-1}$, and Sn ion concentration of 0.0001 mol $L^{-1}$. The pH calculation range was set to 0-7 with a calculation interval of 0.1.

67. Lv, X. *et al.* Regulation mechanism of selective adsorption-hydration effects in chalcopyrite-molybdenite flotation separation using 2-iminothiazolidin-4-one (ITO) as chalcopyrite depressant: Experimental and DFT studies. *Colloids Surf. Physicochem. Eng. Asp.* **725**, 137510 (2025).

68. Yalcin, O., Wachs, I. E. & Onal, I. Role of chromium in Cr–Fe oxide catalysts for high temperature water-gas shift reaction – A DFT study. *Int. J. Hydrog. Energy* **46**, 17154–17162 (2021).

69. Nolan, M. Charge transfer and formation of reduced $Ce^{3+}$ upon adsorption of metal atoms at the ceria (110) surface. *J. Chem. Phys.* **136**, 134703 (2012).

70. Yang, Z., Luo, G., Lu, Z. & Hermansson, K. Oxygen vacancy formation energy in Pd-doped ceria: A DFT+U study. *J. Chem. Phys.* **127**, 074704 (2007).

71. Sima, Y., Zheng, M. & Zhou, X. DFT study of Al-doped $In_2O_3$ with surface frustrated lewis pair sites for enhanced nitrogen reduction efficiency. *J. Mater. Sci.* **60**, 7374–7392 (2025).

**Issue 9:**

It is not difficult to find studies in the literature that use $H_2SO_4$ for the solubilization of ITO, which differs from the result shown in Figure 1F. Wouldn't it be better to carry out this leaching first and, after this process, apply electrolysis? Regenerating the acid in the process and

recovering the metals? Is the use of ITO as an electrode really that advantageous? I was not convinced.

**Discussion:**

Thank you for your valuable comments and careful analysis.

As you mentioned, numerous studies have investigated ITO recovery via acid leaching. However, conventional acid leaching typically involves more complicated operations, greater dosages of additives, and longer leaching times. Specifically, acid leaching processes usually require heating improve leaching efficiency, and strong oxidants such as $H_2O_2$ are commonly used to enhance the leaching efficiency of indium and tin ions. This not only reflects the low recovery efficiency of traditional acid leaching but also indicates its strict requirements for reaction temperature and electrolyte composition. In addition, acid leaching imposes high pretreatment demands on ITO feedstocks, which usually require crushing and grinding into fine and uniform powders to improve ITO leaching efficiency. This undoubtedly increases operational complexity and the risk of impurity introduction. Therefore, the two-step approach of leaching followed by electrolysis for ITO recovery suffers from long processing times and low recovery efficiency, which severely limit improvements in production efficiency.

In the present process, ITO is directly employed as the anode. Under the effect of an applied electric charge, the metal–oxygen bonds in ITO are broken, enabling rapid dissolution of ITO, which is fundamentally different from the pure chemical dissolution in acid leaching. Furthermore, the dissolved indium and tin ions can be directly reduced to indium–tin alloy products at the cathode. This not only achieves process integration but also significantly reduces the time required for the recovery process. In addition, compared with the two-step acid leaching-electrolysis method, the proposed method uses waste ITO targets as the anode, avoiding the use of heterogeneous anodes in the electrolysis step of the two-step process, thereby further reducing the risk of impurity introduction. As shown in Figures 1e and 1f, under charge-induced assistance, the dissolution amount of the ITO anode within 1.5 h far exceeds that without applied current over 12 h, and the enrichment of metal ions in the solution proceeds much more rapidly. The purity analysis of the product (**Figure 6d**) also confirms a substantial reduction in impurity levels.

Based on the above analysis, compared with the traditional two-step acid leaching–electrolysis

method, the proposed technology exhibits distinct advantages in improving dissolution efficiency, shortening the process flow, and reducing impurity content.

**Reviewer #2:**

I co-reviewed this manuscript with one of the reviewers who provided the listed reports. This is part of the *Nature Communications* initiative to facilitate training in peer review and to provide appropriate recognition for Early Career Researchers who co-review manuscripts.

**Discussion:**

We sincerely appreciate your co-review.

**Reviewer #3:**

**Issue 1:**

Figure 1 demonstrates a sharp increase in the dissolution rate of anodic ITO under applied current compared to non-electrified conditions. This intriguing phenomenon underpins the authors' approach to recycling spent ITO. The mentioned electrolysis was conducted in acidic media, would anodic dissolution occur in neutral or alkaline electrolytes? Moreover, would the dissolved metal ions undergo direct precipitation? Such behavior could potentially diversify the recoverable products of this technology, and the authors are encouraged to briefly discuss this possibility.

**Discussion:**

We gratefully acknowledge your question. The solution used in this study was an acidic medium, where metal ions were released from the ITO anode into the electrolyte under the action of charges, and subsequently transferred to the cathode for reduction and deposition. Since ion dissolution is driven by charge-induced effects, ITO should theoretically undergo anodic dissolution even in neutral or alkaline electrolytes. To verify this feasibility, electrolysis experiments were carried out in aqueous solutions of $Na_2SO_4$/NaCl-NaOH, with ITO as the anode and a titanium plate as the cathode. After applying current, white flocculent precipitates were clearly observed near the anode. After collection, and drying at 60 °C, the main phase was identified as $In(OH)_3$, confirming that the white precipitates formed during electrolysis were primarily $In(OH)_3$ (**Figure s1**).

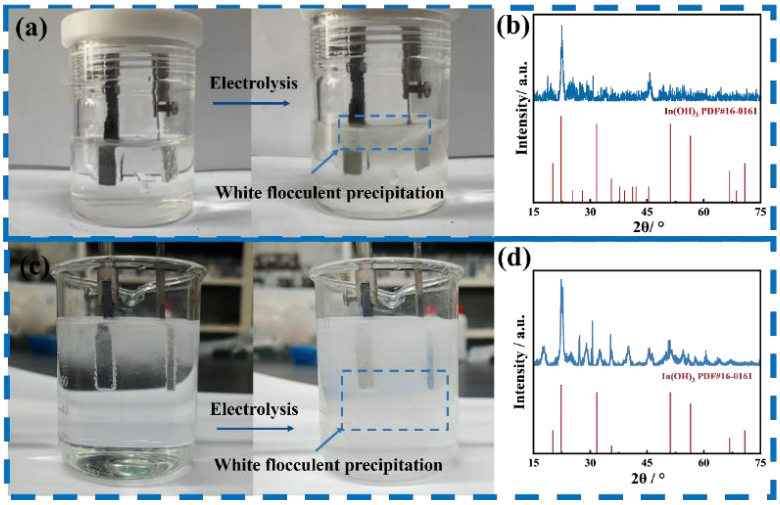


**Figure s1.** Anodic dissolution of s-ITO under neutral and alkaline conditions. (a) Optical photos of precipitate produced in $Na_2SO_4$ solution ($Na_2SO_4$ concentration: 1 mol $L^{-1}$, current density: 0.116 A $cm^{-2}$), (b) XRD patterns of cathode products; (c) Optical photos of precipitate produced in NaOH-NaCl solution (pH=8.5, NaCl concentration: 1 mol $L^{-1}$, current density: 0.116 A $cm^{-2}$); (d) XRD patterns of cathode products.

These results validate the hypothesis you proposed: ITO undergoes anodic dissolution under charge effects not only in acidic media but also in neutral and alkaline media. In acidic media, metal ions migrate to the cathode and are reduced to form alloys; in neutral or alkaline media, ions dissolved from the anode rapidly combine with hydroxide ions in the electrolyte to form hydroxide precipitates. Such pH-dependent product diversity renders the present technology capable of producing recyclable products with versatility, and also points to a new research direction.

**Issue 2:**

In order to improve the cathodic current efficiency, the authors introduced initial indium ions into the electrolyte to reduce current wastage during the initial electrolysis stage—a strategy strongly supported by the key data in Figure 5d. How does the indium ion concentration evolve throughout the electrolytic process, and to what extent can dynamic equilibrium be maintained? It is recommended that the authors perform time-dependent measurements of the electrolyte's indium ion concentration to elucidate this behavior.

**Discussion:**

We greatly appreciate the reviewer's recognition of the effectiveness of our initial indium ion

supplementation strategy in improving cathode current efficiency.

In the absence of added indium ions, the concentrations of indium and tin ions in the electrolyte are theoretically very low at the early stage of electrolysis, which is the main cause of the low cathode current efficiency. As electrolysis proceeds, indium and tin ions gradually accumulate in the electrolyte until the ion concentrations near the cathode and anode reach equilibrium. To verify this hypothesis, we designed the experiments shown in **Figure S15**. We systematically measured the time-dependent composition of the electrolyte at three different positions—near the anode, in the bulk electrolyte, and near the cathode—at 20-minute intervals. The results are presented below.

(1) Temporal evolution of indium ion concentration

As shown in **Figure S15a**, at 20 min after the start of electrolysis, the indium ion concentrations near the anode, in the middle region of the electrolyte, and near the cathode were 2355 mg $L^{-1}$, 2105 mg $L^{-1}$, and 2170 mg $L^{-1}$, respectively. The higher concentration near the anode indicates active anodic dissolution. At 40 min, the concentrations increased to 6350 mg $L^{-1}$ (anode side), 3535 mg $L^{-1}$ (middle region), and 4275 mg $L^{-1}$ (cathode side), demonstrating gradual ion accumulation. Due to mass transport limitations, the ion concentration near the cathode was lower than that near the anode. At 80 min, the concentrations were 6115 mg $L^{-1}$ (anode side), 5585 mg $L^{-1}$ (middle region), and 6400 mg $L^{-1}$ (cathode side). The concentration near the anode remained almost unchanged, while that near the cathode increased to a level comparable to the anode side, and the indium ion concentration in the electrolyte became stable.

(2) Temporal evolution of tin ion concentration

**Figure S15b** presents the corresponding tin ion concentration distribution. At 20 min, the concentrations near the anode, in the middle region of the electrolyte, and near the cathode were 91.0 mg $L^{-1}$, 74.5 mg $L^{-1}$, and 79.5 mg $L^{-1}$, respectively. By 40 min, these values increased to 192.5 mg $L^{-1}$, 107.5 mg $L^{-1}$, and 122.2 mg $L^{-1}$, following a similar trend to that of indium ion. At 80 min, the concentrations were 172.3 mg $L^{-1}$ (anode side), 154.5 mg $L^{-1}$ (middle region), and 174.2 mg $L^{-1}$ (cathode side). The concentration near the anode decreased slightly, while those in the middle region and near the cathode increased, with the cathode-side concentration becoming comparable to the anode side, and the tin ion concentration in the electrolyte became stable.

These results confirm that the metal ion concentrations in the electrolyte increase gradually with electrolysis time and eventually reach a stable state, with comparable concentrations at the anode and cathode sides. This provides experimental support for adding indium ions prior to electrolysis, which can artificially accelerate the enrichment of metal ions in the electrolyte and balance the ion concentrations between the anode and cathode sides.

**The related revision in the main text:**

**Lines 373-383 in Results Section:**

To monitor the variations in the concentration of metal ions during the electrolysis process, samples were collected from different regions of the electrolyte at various electrolysis stages for the determination of indium and tin ion concentrations (**Figure S15**). Prior to the electrolysis process, no indium and tin ions were present in the electrolyte. After 20 min of electrolysis, the concentrations of indium and tin ions increased significantly, with the highest concentration appearing at the anode side. As electrolysis continued, the metal ion concentrations increased further. At 80 min, the metal ion concentration at the anode became nearly stable, while the concentration at the cathode was comparable to that at the anode. Therefore, to achieve a synergy between the anodic and cathodic current efficiencies, additional indium ions should be introduced into the electrolyte to compensate for the current loss caused by insufficient ion concentration at the initial stage.

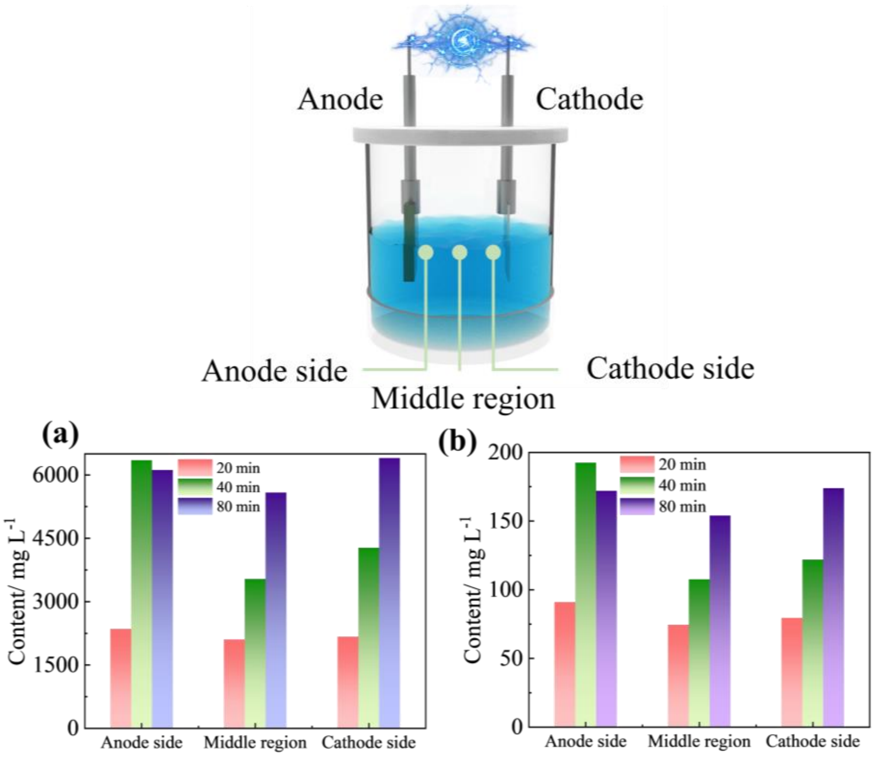


**Figure S15**. Concentrations of indium and tin ions at different electrolysis time in $H_2SO_4$-NaCl aqueous solution using spent ITO target as the anode ($H_2SO_4$ concentration: 5.52 mol $L^{-1}$; NaCl concentration: 1.2 mol $L^{-1}$; current density: 0.116 A $cm^{-2}$): (a) Indium ion concentrations; (b) Tin ion concentrations.

**Issue 3:**

The fitted curves in Figures S6 and S7 are intended to further explain the electrochemical behavior of indium and tin ions in electrolytes, but the fitting degree of the curves is not high. Although this outcome does not invalidate the authors' conclusions, it is advisable to repeat the experiments to obtain better-fitted curves to address the minor issue.

**Discussion:**

We greatly appreciate the reviewer's valuable suggestions.

We have repeated the corresponding experiments and refitted the datasets to improve the fitting quality of the curves in Figures S6 and S7. Furthermore, considering that the cyclic voltammetry profiles together with their quantitative fittings can sufficiently elucidate the electrochemical reaction behavior of the ions, the supplementary square wave voltammetry (SWV) curves, originally intended for further verification, have been removed from the revised manuscript.

**The related revision in the Supplementary Information:**

**Figures S10 and S11:**

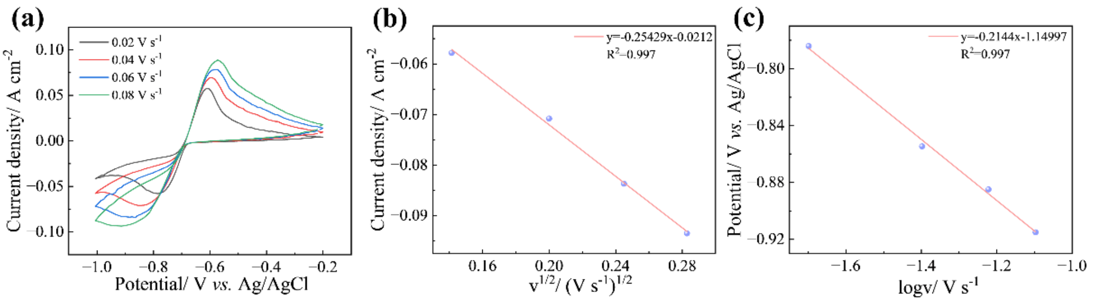


**Figure S10**. CV and derived curves in $H_2SO_4$-NaCl solution containing indium ions (indium ion concentration: 0.01 mol $L^{-1}$, $H_2SO_4$ concentration: 5.52 mol $L^{-1}$, NaCl concentration: 1.2 mol $L^{-1}$): (a) CV curves at different scan rates; (b) The fitting curve of peak current and square root of scan rate; (c) The fitting curve of potential and logarithm of the scan rate.

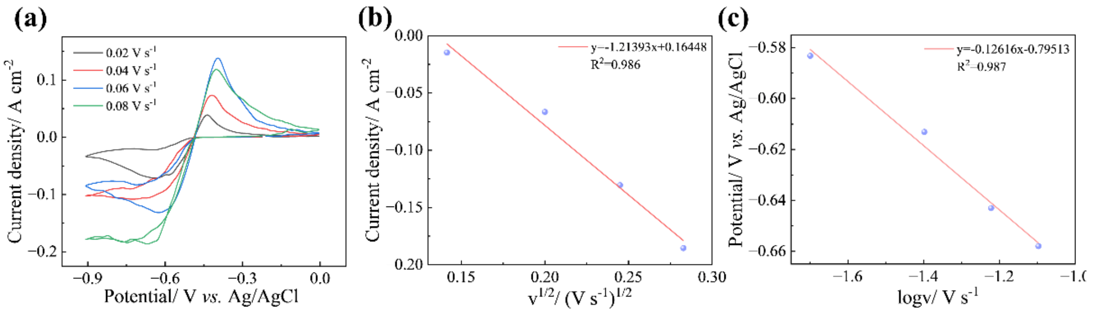


**Figure S11**. CV and derived curves in $H_2SO_4$-NaCl solution containing tin ions (tin ion concentration: 0.01 mol $L^{-1}$, $H_2SO_4$ concentration: 5.52 mol $L^{-1}$, NaCl concentration: 1.2 mol $L^{-1}$): (a) CV curves at different scan rates; (b) The fitting curve of peak current and square root of scan rate; (c) The fitting curve of potential and logarithm of the scan rate.

**Issue 4:**

The anode itself is composed of spent ITO target material, which eliminates the need for heterogeneous anodes and thereby prevents the risk of impurity introduction from foreign materials—a key advantage of this technology. However, as electrolysis progresses, impurities inevitably accumulate in the electrolyte. When the product purity falls below the requirements, electrolyte replacement becomes necessary. How might the spent electrolyte be treated? Is it analogous to the treatment strategies for electrolytes in copper electrorefining?

**Discussion:**

Thank you for your insightful comment. The management of waste electrolyte is closely

associated with the comprehensive sustainability evaluation of the proposed technology. During electrolysis, inevitable accumulation of impurities occurs in the electrolyte, as clearly evidenced by our supplementary experimental results. With increasing electrolysis cycles, the concentrations of major impurities (Al, Mn, Fe, and Si) exhibit a slight increment. After ten cycles, their contents rise from 0.2 ppm to 1.4 ppm, 0.02 to 0.09 ppm, 0.7 ppm to 1.9 ppm, and 7.3 ppm to 10.9 ppm, respectively. Despite such elevation, the impurity increase remains marginal, thereby imposing a negligible impact on product purity. These results verify the recyclability of the adopted electrolyte. Nevertheless, continuous accumulation of impurities will eventually compromise product purity upon prolonged operation, necessitating periodic electrolyte renewal when the impurity threshold is exceeded. As suggested, the waste electrolyte can be treated following the conventional purification strategies applied in copper electrolytic refining, wherein impurities are removed via segmented processing routes. This represents a general and well-established approach for electrolyte management in aqueous electrolytic refining processes.

**The related revision in the main text and Supplementary Information:**

**Lines 82-100 in Supplementary Information:**

**Section 5: Reusability experiment of the electrolyte**

To evaluate the reusability of the electrolyte, the same electrolysis process was continuously repeated in the original electrolyte after each electrolysis run, with a total of 10 consecutive runs conducted. The duration of each electrolysis is 1.5 h. The electrolyte was composed of 100 mL of 5.52 mol $L^{-1}$ $H_2SO_4$ and 1.2 mol $L^{-1}$ NaCl. For each electrolysis run, cathodes (titanium plates) and anodes (s-ITO) with the same dimensions were employed, and both the electrode spacing and current density (0.116 A $cm^{-2}$) were maintained constant. Impurity analysis was conducted on the products and electrolyte collected subsequent to the second run, accompanied by quantitative determination of the hydrogen ion concentration within the electrolyte. Subsequent sampling and characterization were implemented at intervals of every other cycle throughout the entire electrolysis process.

The determination of hydrogen ion concentration was achieved by measuring the pH value of the diluted solution: 100 μL of the electrolyte was pipetted and diluted to 10 mL with deionized water, followed by pH measurement using a PHS-25 benchtop pH meter, and finally

the hydrogen ion concentration in the solution was calculated according to Formula 5. The calculated $H^+$ concentration was then multiplied by 100 to obtain the $H^+$ concentration in the original electrolyte.

$$pH=-\lg C \quad (5)$$

where $C$ is the hydrogen ion concentration (mol $L^{-1}$).

**Lines 462-474 in Results Section:**

In contrast, the electrolyte employed in this technology exhibits excellent reusability. After 10 cycles of repeated electrolysis (each cycle duration: 1.5 h, current density: 0.116 A $cm^{-2}$), the $H^+$ concentration in the electrolyte remained nearly constant (**Figure S20a**; the measurement method of $H^+$ concentration is provided in **Section 5** of **Supplementary Information**), whereas the concentrations of the main impurities increased gradually (**Figure S20b**). This observation indirectly demonstrates the impurity purification effect of the proposed process, in which most impurities are retained in the electrolyte during electrolysis. Accordingly, the contents of main impurities in the final cathodic product were still markedly reduced (**Figure S20c**), similar to those of the product obtained from a single electrolysis run. With an increasing number of cycles, the electrolysis process gradually stabilized, and the current efficiency in the later stage remained steady at approximately 71% (**Figure S20d**). These results clearly confirm that the proposed technology enables the repeated reuse of the electrolyte, thereby significantly reducing wastewater generation.

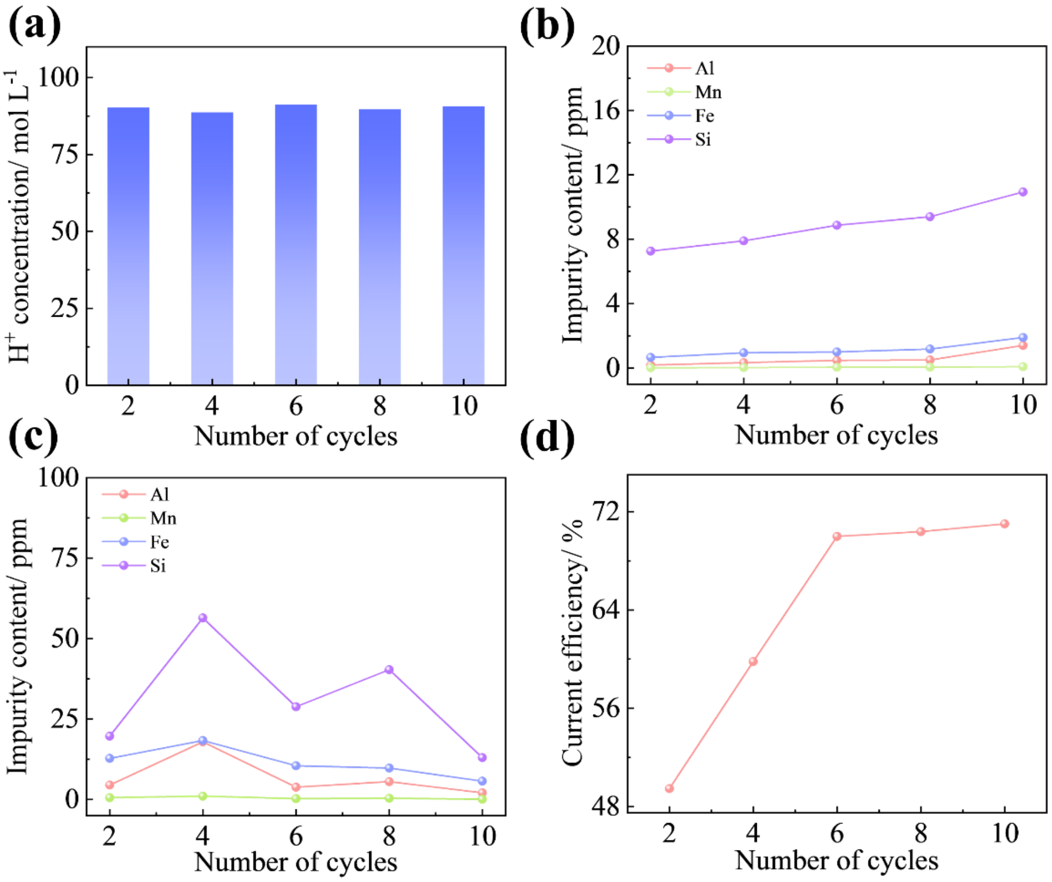


**Figure S20**. Evaluation of electrolyte stability under multi-cycle electrolysis operation (the duration of each electrolysis is 1.5 h and current density is 0.116 A $cm^{-2}$; the electrolyte is composed of 100 mL of 5.52 mol $L^{-1}$ $H_2SO_4$ and 1.2 mol $L^{-1}$ NaCl.). (a) Changes of $H^+$ concentration of electrolyte; The changes of the main impurity content after 10 cycles of electrolysis: (b) electrolyte, (c) cathode product; (d) Cathode current efficiency.

**Issue 5:**

The 100 A scale-up experiment depicted in Figure 6 convincingly validates the technical scalability and establishes foundation for industrial implementation. Regarding a minor issue: Are there specific requirements for the dimensions and geometry of waste ITO targets when used as anodes? and do the spent ITO need to be pre-processed?

**Discussion:**

We appreciate your comment. During electrolysis, the charge drives the cleavage of metal–oxygen bonds in the ITO waste anode, leading to the dissolution of In and Sn ions, which subsequently migrate to the cathode for reduction. According to the above dissolution mechanism, the shape of the ITO anode does not affect its anodic dissolution behavior, and thus

no specific requirement is imposed on the geometry of the ITO waste target. In practice, from our 1 A-scale recovery to the scaled-up 100 A-scale recovery, the ITO anodes used exhibited different geometries, yet none affected their anodic dissolution during electrolysis (**Figure s2**, the anodic current efficiencies are all above 70%). In summary, this technique imposes no restriction on the anode geometry, which reduces the machining procedures prior to electrolysis.

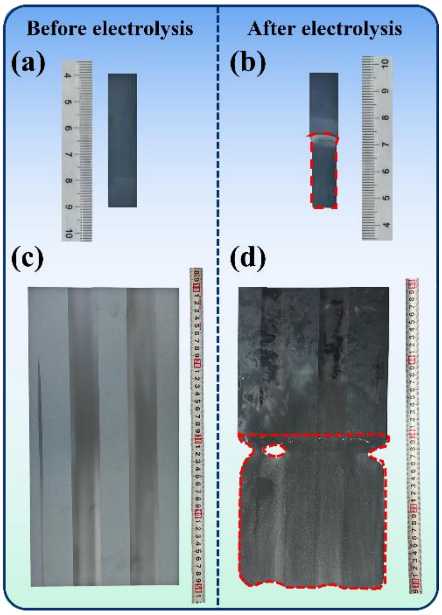


**Figure s2.** Optical images showing the consumption of ITO anodes with different shapes and sizes during electrolysis in aqueous solution containing 5.52 mol $L^{-1}$ $H_2SO_4$ and 1.2 mol $L^{-1}$ NaCl at a current density of 0.116 A $cm^{-2}$. (a) before electrolysis; (b) after electrolysis (current: 0.5 A, time: 1.5 h); (c) before electrolysis; (d) after electrolysis (current: 100 A, time: 1 h).

**Issue 6:**

The gas symbol for the reaction product in Figure 1 has been omitted by the author, and it should be supplemented. The font size in the figures of the manuscript is somewhat small (such as in Figure 3a), and the authors can slightly increase it to improve readability. The author should carefully proofread the text to eliminate grammatical errors.

**Discussion:**

We greatly appreciate your constructive suggestions, which are of great importance for improving the quality of this paper.

Regarding the issues of font size and gas symbols in the figures, we have carefully checked and revised all figures in the manuscript to ensure the accuracy and presentation quality of the

information displayed. In addition, we have carefully proofread the language throughout the text to ensure fluency and grammatical correctness.

**The related revision in the main text:**

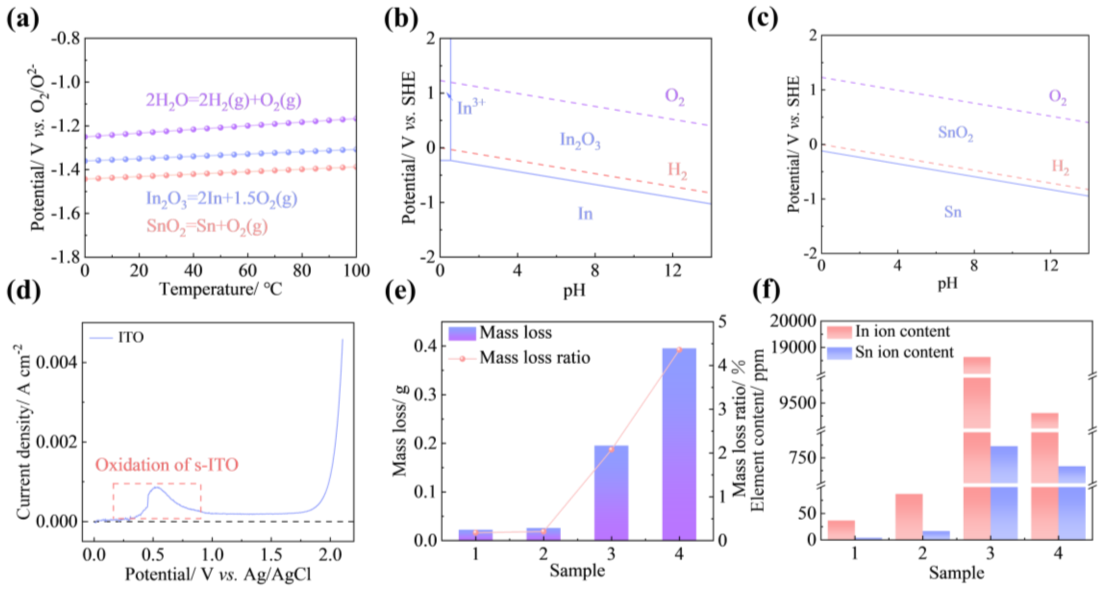


**Figure 1**

**Reviewer #4:**

Overall Assessment: The manuscript addresses an important topic related to electrochemical recovery of indium from spent ITO materials. However, in its current form, the conclusions are frequently drawn without sufficient mechanistic evidence or experimental validation. In several instances, interpretations rely heavily on speculation rather than direct data or appropriate literature support, which does not meet the level of rigor expected for *Nature Communications*. In addition, critical experimental details are missing throughout the manuscript, limiting reproducibility and undermining confidence in the reported results. Taken together, the conceptual overstatements, lack of mechanistic substantiation, and insufficient methodological transparency represent serious shortcomings. As a result, I believe, the manuscript, even after revision, would not be suitable for publication in *Nature Communications*.

**Discussion:**

We sincerely appreciate your careful review and valuable comments, which are of great guiding significance for improving the quality of this manuscript. We fully accept all comments and will revise the manuscript point by point to further refine it and meet the rigorous publication standards of *Nature Communications*.

**Issue 1:**

The Introduction would benefit from a more balanced and structured comparison between pyrometallurgical and hydrometallurgical recycling routes. While the limitations of conventional pyro- and hydro-metallurgical processes are emphasized, their continued industrial adoption suggests that these routes also possess practical advantages (e.g., robustness, scalability, or tolerance to feedstock variability) that should be acknowledged. A concise discussion of both strengths and weaknesses would establish a more objective baseline for positioning electrochemical strategies. In addition, Line 46 mentions electrochemical treatments integrated with hydrometallurgy; however, the distinction between previously reported electrochemical approaches and the specific electrochemical strategy employed in this work remains unclear. Given that the present study is fundamentally electrochemical in nature, the authors should explicitly clarify how their approach differs from, or advances beyond, existing electrochemical recycling and recovery methods. Expanding the discussion of prior

electrochemical metal recovery studies that exploit cathodic or combined anodic–cathodic reactions for high-purity metal recovery would significantly strengthen the Introduction (e.g., *The Scientific World Journal*, 2015, 494015; *Energy Storage Materials*, 2025, 82, 104646).

**Discussion:**

We sincerely appreciate the reviewer's constructive comments on the Introduction section, which have significantly helped us improve the rigor, balance, and clarity of our manuscript. We have carefully addressed all the raised issues, and the corresponding revisions in the Introduction are detailed as follows:

(1) Balanced comparison between pyrometallurgical and hydrometallurgical recycling routes

As suggested by the reviewer, we have supplemented the practical advantages of conventional pyrometallurgical and hydrometallurgical processes, which were not fully acknowledged in the original manuscript. Specifically, we have clarified that pyrometallurgy possesses prominent industrial merits such as strong adaptability, good process robustness, and high scalability for large-scale feedstock treatment; hydrometallurgy, on the other hand, has the advantages of relatively high metal selectivity and mature engineering applications. We have also retained the discussion on their inherent limitations. This balanced discussion of both strengths and weaknesses of the two conventional routes has established a more objective baseline for positioning our electrochemical recycling strategy, as recommended.

(2) Distinction between electrolysis involved in hydrometallurgical recovery, direct molten salt electro-deoxidation and the proposed strategy

We fully agree with the reviewer's comment that the distinction between existing electrolytic-related approaches (including electrolysis involved in hydrometallurgical recovery and direct molten salt electro-deoxidation) and our proposed strategy was unclear. To address this issue, we have explicitly clarified the fundamental differences and advancements of our work by comparing the three methods in detail.

First, regarding the electrolysis involved in hydrometallurgical recovery, it is merely a post-treatment step in the entire hydrometallurgical process. This electrolytic step can only be carried out after a prolonged pre-leaching process, where s-ITO is leached using large amounts of acids, alkalis, or extractants to dissolve indium and tin ions into the leachate. The subsequent electrolysis is only used to recover indium metal or related compounds from the leachate, rather

than directly treating s-ITO. Notably, this electrolytic method relies heavily on the pre-leaching step, which inevitably generates massive chemical wastewater and leads to complex processes and long cycles.

Second, the direct molten salt electro-deoxidation method (including our previous related work) belongs to a cathodic electro-deoxidation process. In this method, s-ITO is used as the cathode, and electro-deoxidation is performed in molten salt systems (e.g., $CaCl_2$-based molten salts) to reduce s-ITO into In-Sn alloys. However, the depolarization effect of the liquid alloy formed after electro-deoxidation can lead to the underpotential deposition of metallic calcium onto the cathode product. Furthermore, the employment of carbon anodes generates carbon oxide gases, and the heterogeneous anode materials increases the risk of elevated impurities in the product.

In sharp contrast, our proposed strategy is an innovative anode dissolution-based electrochemical method, which is essentially different from the above two existing approaches. Specifically, our method directly uses s-ITO as a soluble anode for electrolysis in an aqueous electrolyte system ($H_2SO_4$-NaCl), without the need for any pre-leaching steps and operating at room temperature. Unlike cathodic electro-deoxidation in molten salts, our strategy leverages the intrinsic properties of s-ITO. Under an applied charge, the bonding force between metal and oxygen within s-ITO is weakened, thereby facilitating the dissolution of metal ions. The dissolved indium and tin ions then migrate to the cathode and are reduced to form high-purity In-Sn alloys. This fundamental difference not only overcomes the drawbacks of the existing methods but also achieves more efficient, green, and simple recycling of s-ITO, fully reflecting the novelty and advancement of our proposed strategy.

All revisions have been incorporated into the updated Introduction of the manuscript, and we believe these changes have significantly improved the quality and persuasiveness of this section. We would like to express our sincere gratitude again for the reviewer's valuable suggestions.

**The related revision in the main text:**

**Lines 41-63 in Methods Section:**

S-ITO is mainly recycled through traditional pyrometallurgical and hydrometallurgical processes, both of which have distinct industrial merits. Pyrometallurgical recovery is characterized by strong industrial adaptability, excellent process robustness, and high scalability, rendering it suitable for large-scale s-ITO feedstock processing; hydrometallurgical recovery

possesses relatively high metal selectivity and a well-established engineering application system[7–9]. Despite these advantages, both traditional processes have inherent limitations. Pyrometallurgy, which typically employs carbon-based materials as reducing agents, is plagued by massive $CO_2$ emissions and extremely high energy consumption[10]. Hydrometallurgy involves the extensive use of acids, alkalis, and other reagents, generates substantial chemical wastewater, features complex processes and lengthy processing cycles, and faces severe environmental pressure from wastewater treatment[11,12]. In recent years, electrolysis methods, featuring short process flows and environmental benignity, have also been adopted for s-ITO recycling. Unlike the electrolysis process in hydrometallurgical recovery, which merely serves as a process for extracting metals via electrolysis after prolonged pre-leaching[6,13,14], the electrolysis methods mentioned in this paper serve as the core process and enable the direct extraction of metals from s-ITO. A typical representative of such electrolysis methods is the molten salt electro-deoxidation technology[15]. Our group has conducted extensive research on the recycling of s-ITO via electro-deoxidation. The utilization of s-ITO as the cathode during electrolysis in $CaCl_2$-based molten salt allows the direct production of In-Sn alloy on the cathode. However, the depolarization effect of the liquid alloy formed after electro-deoxidation can lead to the underpotential deposition of metallic calcium on the cathode product. Furthermore, the employment of carbon anodes generates carbon oxide gases, and the anode materials increases the risk of elevated impurities in the product[16,17].

7. Tian, Q., Meng C., Xu Z., Qiao J., & Guo X. A review of indium separation and extraction technologies from secondary waste resources. *Hydrometallurgy* **242**, 106690 (2026).

8. Sedlakova-Kadukova, J. *et al.* Advanced treatment of flat panel displays through chemical and biological methods: promises for the future. *Rev. Environ. Sci. Biotechnol.* **25**, 13 (2026).

9. Bellemans, I., De Wilde, E., Moelans, N. & Verbeken, K. Metal losses in pyrometallurgical operations - A review. *Adv. Colloid Interface Sci.* **255**, 47–63 (2018).

10. Ma, E., Lu, R. & Xu, Z. An efficient rough vacuum-chlorinated separation method for the recovery of indium from waste liquid crystal display panels. *Green Chem.,* **14**, 3395-3401 (2012).

11. Liu, J. S. *et al.* Extraction and separation of In(III), Ga(III) and Zn(II) from sulfate solution

using extraction resin. *Hydrometallurgy* **82**, 137-143 (2006).
12. Matsumiya, M., Sumi, M., Uchino, Y. & Yanagi, I. Recovery of indium based on the combined methods of ionic liquid extraction and electrodeposition. *Sep. Purif. Technol.* **201**, 25–29 (2018).
13. Cao, Y. *et al.* Leaching and purification of indium from waste liquid crystal display panel after hydrothermal pretreatment: Optimum conditions determination and kinetic analysis. *Waste Manag.* **102**, 635–644 (2020).
14. Zhang, K. *et al.* Recycling indium from waste LCDs: A review. *Resour. Conserv. Recycl.* **104**, 276–290 (2015).
15. Su, X. *et al.* Recovery and reuse of spent ITO targets through electrochemical techniques. *J. Electrochem. Soc.* **169**, 023507 (2022).
16. Su, X., Li, S., Liu, C., He, J. & Song, J. Reduction mechanism of spent ITO target in $CaCl_2$ molten salt. *Sep. Purif. Technol.* **299**, 121778 (2022).
17. Song, J., Li, S., Lv, Z. & He, J. In-Sn alloy extraction through novel process for recycling of spent ITO targets. *Sustain. Mater. Technol.* **41**, e01027 (2024).
18. Lv, X. *et al.* Upcycling waste ITO powders through integrated leaching enhancement and selective deposition. *Sustain. Mater. Technol.* **46**, e01710 (2025).

**Issue 2:**

An important unresolved issue concerns whether the observed deposits correspond to simple codeposition or true alloy formation. Considering that the In-O bond is weaker than the Sn-O bond, preferential formation of In-rich phases rather than homogeneous alloys would be expected during electrolysis. Quantitative ICP analysis of In and Sn in the deposits should be provided to substantiate claims of alloy formation. In addition, high-resolution TEM and STEM analyses are necessary to clarify the phase structure and elemental distribution at the nanoscale.

**Discussion:**

Thank you for your insightful comment. To accurately identify whether the product originates from co-deposition to form a mechanical mixture or alloy, we have performed comprehensive characterizations on the as-obtained samples.

ICP analysis reveals that the contents of indium and tin in the product reach 93.03 wt% and

6.66 wt%, respectively, which alone cannot confirm alloy formation. The most critical evidence derives from XRD characterization. A comparison between the XRD patterns of the cathode product and standard reference cards reveals characteristic diffraction peaks corresponding to In–Sn alloys, verifying the formation of $In_{0.959}Sn_{0.041}$. This unambiguously confirms that the cathode product is an In–Sn alloy rather than a mechanical mixture.

Furthermore, TEM measurements were conducted for supplementary validation. The product exhibits diverse lattice orientations, and the diffraction rings show no obvious preferred orientation, indicating a poorly crystalline polycrystalline structure. Additional elemental mapping demonstrates homogeneous distribution of In and Sn at the nanoscale. Such uniform elemental dispersion further corroborates the alloy nature of the product, since it is difficult for a mechanical mixture to achieve uniform mixing of the two metallic components.

The above analyses have been integrated into the revised manuscript for clarity and rigor.

**The related revision in the main text:**

**Lines 335-339 in Results Section:**

After electrolysis in $H_2SO_4$ solution, a thin layer of silvery-white metal was deposited on the cathode, and XRD analysis confirmed that the product was In-Sn alloy (**Figure S14a**). With the addition of NaCl, the cathode product exhibited a porous and sponge-like morphology with a significantly increased mass. XRD results verified that the product was still In-Sn alloy (**Figure S14b**).

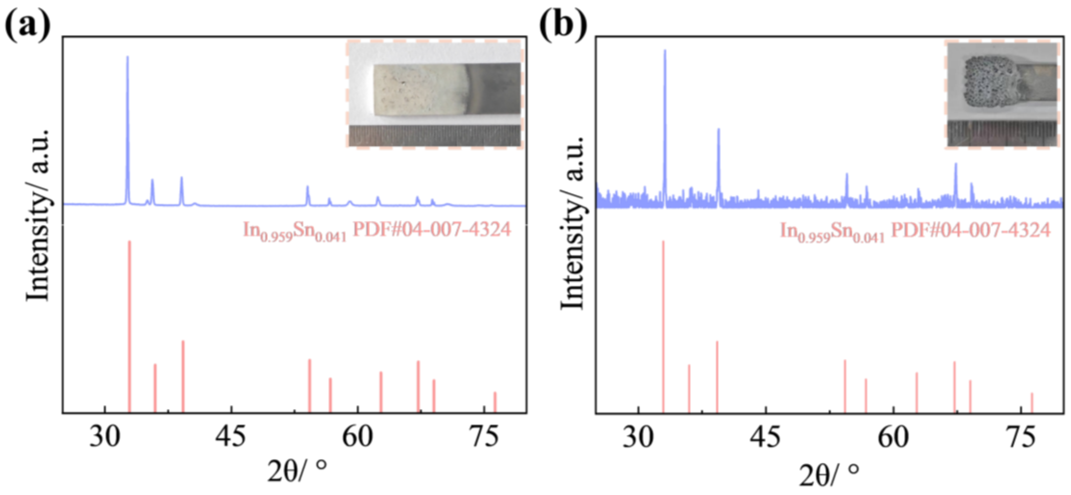


**Figure S14**. XRD pattern of cathodic products: (a) $H_2SO_4$ solution, (b) $H_2SO_4$-NaCl solution ($H_2SO_4$ concentration: 5.52 mol $L^{-1}$, NaCl concentration: 1.0 mol $L^{-1}$, current density: 0.072 A $cm^{-2}$, duration: 1.5 h).

**Lines 404-406 in Results Section:**

TEM results of the cathode product further demonstrate that it exhibits a polycrystalline structure with low crystallinity (**Figure S17a**) and uniform elemental distribution at the nanoscale (**Figure S17b** and **S17c**).

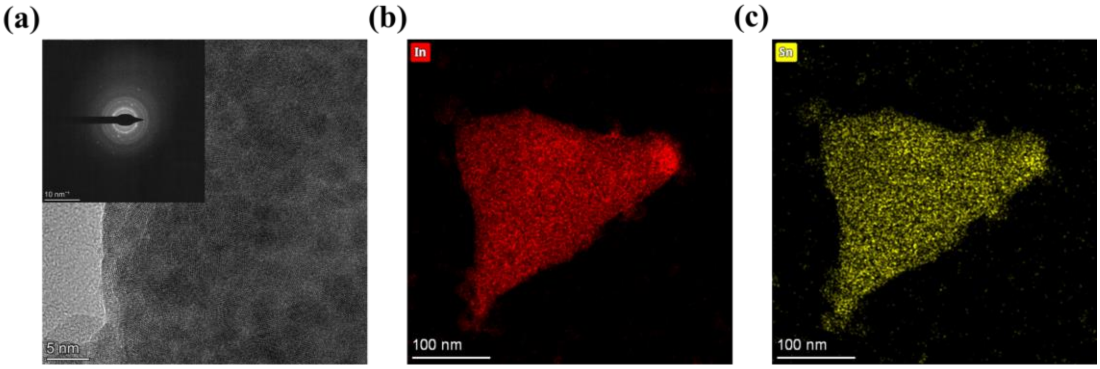


**Figure S17.** TEM images of cathode products and EDS spectra of In and Sn elements (current density: 0.116 A $cm^{-2}$, duration: 1.5 h, $H_2SO_4$ concentration: 5.52 mol $L^{-1}$, NaCl concentration: 1.2 mol $L^{-1}$).

**Issue 3:**

The observed metal release behavior may partially overlap with chloride-induced corrosion processes rather than being purely electrochemical in origin. Additional characterization is required to distinguish electrochemical dissolution from chemical corrosion, particularly under $Cl_2$-evolving or chloride-mediated conditions. Further investigation into the role of chlorine species is therefore necessary.

**Discussion:**

Thank you for your valuable comments. The reviewer's skepticism regarding the underlying mechanism arises from the potential formation of $Cl_2$ at the anode during electrolysis, wherein the generated $Cl_2$ may react with water to produce HCl. It remains necessary to further verify whether other chlorine-containing species exert any influence on the chemical dissolution of s-ITO.

To clarify the effect of $Cl_2$, $Cl_2$ was purged into an $H_2SO_4$–NaCl solution, and acid leaching of s-ITO was simultaneously performed in an $H_2SO_4$-HCl solution under open-circuit conditions. After maintaining 1.5 h, the mass loss of s-ITO was evaluated systematically. The results reveal negligible mass loss of s-ITO in the absence of current. This demonstrates that such chlorine

species alone can hardly facilitate the dissolution of s-ITO under non-electrolytic conditions. Furthermore, Tafel curves indicate that the addition of NaCl to the $H_2SO_4$ solution induces a negative shift in the corrosion potential of s-ITO. This further corroborates that NaCl promotes the electrochemical corrosion of s-ITO.

The above analyses have been incorporated into the revised manuscript in full detail.

**The related revision in the main text:**

**Lines 104-116 in Results section:**

Upon the application of current, $Cl^-$ on the anode surface may be oxidized to $Cl_2$, which can further react with water to generate HCl. Therefore, the potential effects of other chlorine species on the dissolution of s-ITO were also investigated. Specifically, $Cl_2$ was introduced into the $H_2SO_4$-NaCl electrolyte (the preparation method of $Cl_2$ is detailed in **Figure S2a**), and the $Cl_2$ gas escaped from the edge of the s-ITO electrode. S-ITO experienced a mass loss of only 0.016 g after a continuous introduction period. Additionally, an acid leaching experiment of s-ITO was conducted in the $H_2SO_4$-HCl solution, and s-ITO experienced a mass loss of only 0.014 g (**Figure S2b**). These results indicate that in the absence of current, these chlorine species ($Cl_2$ and HCl) are hardly capable of independently accelerating the dissolution of s-ITO. The Tafel curves (**Figure S3**) further reveal that the incorporation of NaCl into the $H_2SO_4$ electrolyte induces a negative shift in the corrosion potential of s-ITO, which confirms that the promotional effect of $Cl^-$ on s-ITO dissolution can be effectively exerted under the action of charge.

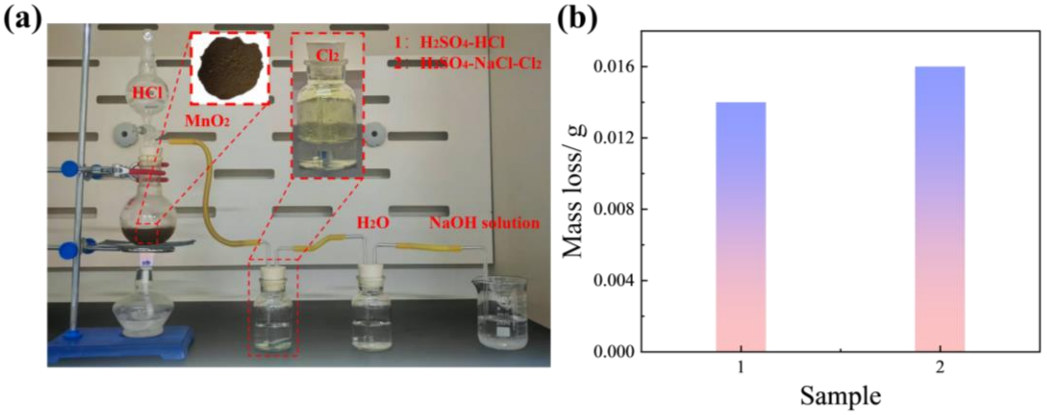


**Figure S2**. Chlorine generation and leaching experiments under different chlorine species conditions. (a) $Cl_2$ generation device (reaction equation: $4HCl+MnO_2=MnCl_2+Cl_2(g)+2H_2O$; the concentration of HCl is 36%-38%, 30 mL (in excess); 5.22 g of $MnO_2$ (approximately 0.06

mol)); (b) Acid leaching results of s-ITO under different conditions (1: 5.52 mol $L^{-1}$ $H_2SO_4$+1.2 mol $L^{-1}$ HCl, 2: 5.52 mol $L^{-1}$ $H_2SO_4$+1.2 mol $L^{-1}$ NaCl+0.06 mol $Cl_2$; Duration: 1.5 h).

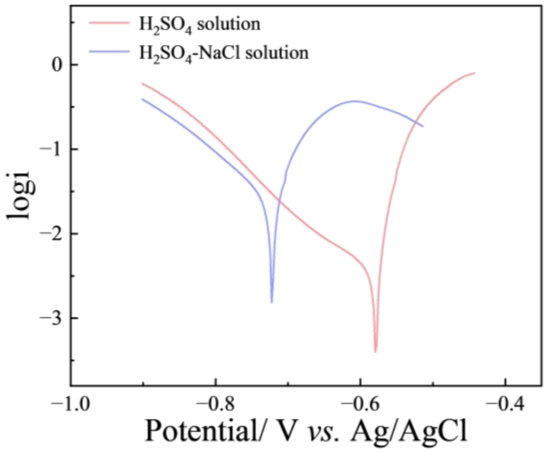


**Figure S3**. Tafel curves of s-ITO in $H_2SO_4$ solution before and after adding NaCl ($H_2SO_4$ concentration: 5.52 mol $L^{-1}$, NaCl concentration: 1.2 mol $L^{-1}$, scan rate: 0.02 V $s^{-1}$).

**Issue 4:**

There is a fundamental terminological inconsistency regarding voltage and potential. In Line 146, the authors refer to “voltage exceeding 1.5 V,” whereas Fig. 1a presents the y-axis as potential (V *vs.* $O_2/O^{2-}$). Voltage and potential are not interchangeable terms and must be clearly distinguished. It should be clarified whether the authors are referring to the cell voltage in a two-electrode configuration or the electrode potential referenced to a specific redox couple.

**Discussion:**

We sincerely thank the reviewer for pointing out the issues regarding the use of the two terms, voltage and potential, and we also express our sincere gratitude to the reviewer for the careful guidance on the standardization of research expression. In response to the terminological inconsistency raised by the reviewers—"the authors refer to 'voltage exceeding 1.5 V' in Line 146, whereas Fig. 1a presents the y-axis as potential (*vs* $O_2/O^{2-}$)"—since all potentials used throughout the entire text are electrode potentials referenced to specific redox couples (the reference for the thermodynamic calculations in Figure1a is $O_2/O^{2-}$; the reference for the thermodynamic calculations in Figure1b and 1c is SHE; the reference used for electrochemical tests is Ag/AgCl) and only the voltage is adopted during the electrolytic recovery of s-ITO (electrolysis under a two-electrode configuration). The detailed specifics have been elaborated in the Methods section. We have comprehensively reviewed and sorted out all relevant

expressions in the full text, clearly indicating the reference couples to ensure the consistency and logical consistency between the text and the figures.

**The related revision in the main text:**

**Lines 78-82 in Results Section:**

According to the results of thermodynamic calculations (**Figure 1a**), the theoretical reduction potentials (*vs.* $O_2/O^{2-}$) of the metal ions in $In_2O_3$ and $SnO_2$ are slightly more negative than the hydrogen evolution reaction (HER) potential (*vs.* $O_2/O^{2-}$) in water[19,20]. In practice, hydrogen evolution usually requires a substantial potential more negative than -1.5 V (*vs.* $O_2/O^{2-}$) due to the high overpotential required for HER.


19. Zou, X. & Zhang, Y. Noble metal-free hydrogen evolution catalysts for water splitting. *Chem. Soc. Rev.* **44**, 5148 (2015).

20. Li, S., Lv, Z., He, J. & Song, J. Electrochemical upcycling of indium-gallium-zinc oxide scraps. *Process Saf. Environ. Prot.* **191**, 2303-2310 (2024).


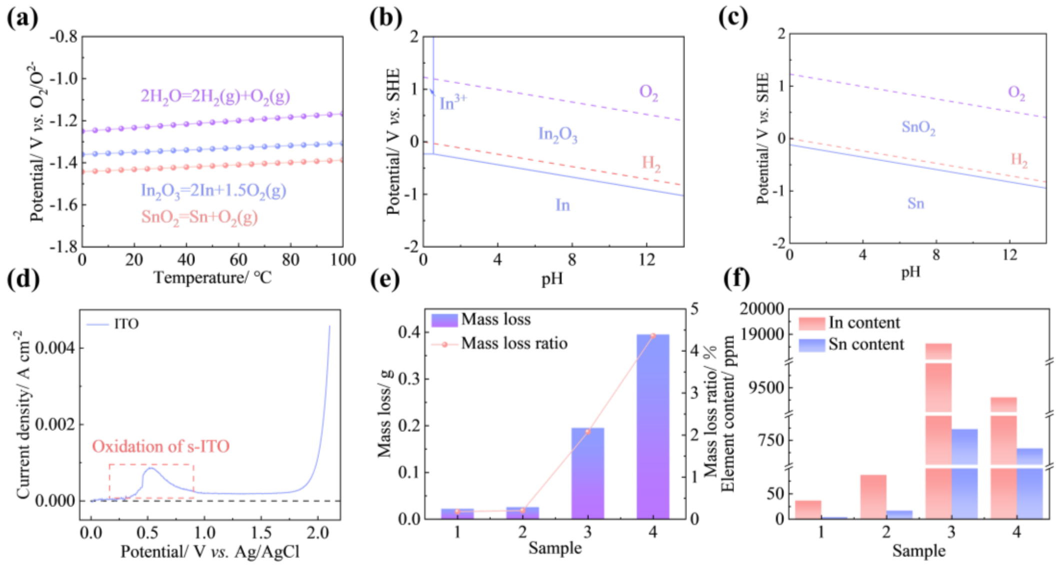


**Figure 1**

**Issue 5:**

In Lines 148–149, the statement that “with decrease in pH metallic indium and tin demonstrate notable regional advantage” is unclear and insufficiently explained. The meaning of “regional advantage” is ambiguous and should be clarified with a more precise electrochemical or physicochemical interpretation.

**Discussion:**

We appreciate the reviewer for identifying this ambiguity. The phrase 'regional advantage' is indeed imprecise and lacks clear electrochemical or physicochemical interpretation. We shall revise this statement with rigorous clarification.

With decreasing pH, metallic indium and tin exhibit expanded thermodynamic stability domains relative to their respective oxide phases. As illustrated in the Figure 1b, 1c, the stability fields of metallic In and Sn progressively broaden under acidic conditions, while the stability regions of $In_2O_3$ and $SnO_2$ contract. This creates a thermodynamically favorable region where metallic indium and tin become the predominant stable phases, thereby conferring a clear advantage over oxide formation under the applied electrochemical conditions.

**The related revision in the main text:**

**Lines 83-87 in Results Section:**

As depicted in the E-pH diagrams (**Figure 1b** and **1c**), with decreasing pH, metallic indium and tin exhibit expanded thermodynamic stability domains relative to their respective oxide phases. This creates a thermodynamically favorable region where metallic indium and tin become the predominant stable phases. At relatively more positive potentials, metallic indium and tin can be obtained.

**Issue 6:**

In Line 154, the authors attribute the observed current fluctuation at the initial stage to electrochemical dissolution of s-ITO. However, this conclusion appears to be drawn solely from current responses without direct supporting evidence. Could these fluctuations instead arise from early-stage surface adsorption, restructuring, or transient interfacial processes? This interpretation requires further justification or additional supporting data.

**Discussion:**

We appreciate the reviewer for raising this important mechanistic question regarding the interpretation of current fluctuations observed in the initial stage of linear sweep voltammetry (LSV) measurements. To rigorously verify our attribution of these fluctuations to the electrochemical dissolution of s-ITO anodes, we performed systematic and repeated comparative LSV studies using different working electrode materials.

When glassy carbon was employed as the working electrode for anodic scanning in the $H_2SO_4$-

NaCl solution, no obvious current response signal was observed prior to electrolyte decomposition, indicating the absence of electrochemical oxidation reactions (**Figure S1**). In contrast, when s-ITO was used as the working electrode for anodic scanning in both $H_2SO_4$ and $H_2SO_4$-NaCl solutions, significant current response signals were detected before electrolyte decomposition (**Figure 1d and S1**). The potentials of these current response signals varied slightly depending on the electrolyte composition, and this phenomenon exhibited excellent reproducibility in repeated experiments.

Through comparative analysis, it can be concluded that the observed current response signals are attributed to the oxidation of s-ITO.

**The related revision in the main text:**

**Lines 88-95 in Results Section:**

Linear sweep voltammetry (LSV) curves (**Figure 1d** and **Figure S1**) also demonstrate that when s-ITO is employed as the working electrode, an obvious current response appears prior to electrolyte decomposition with the potential scanned positively (see the dashed box region in **Figure 1d**). When glassy carbon is used as the working electrode, no current response on LSV curve is observed prior to electrolyte decomposition. It can be concluded from the comparison of LSV curves that the current response corresponds to the electrochemical dissolution of s-ITO, including the oxidation of oxygen ions and the release of indium and tin ions.

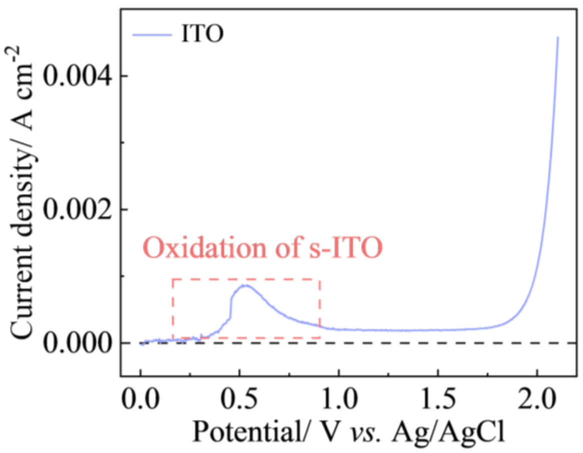


**Figure 1d**. LSV curve of s-ITO in $H_2SO_4$-NaCl solution (scan rate: 0.1 V $s^{-1}$)

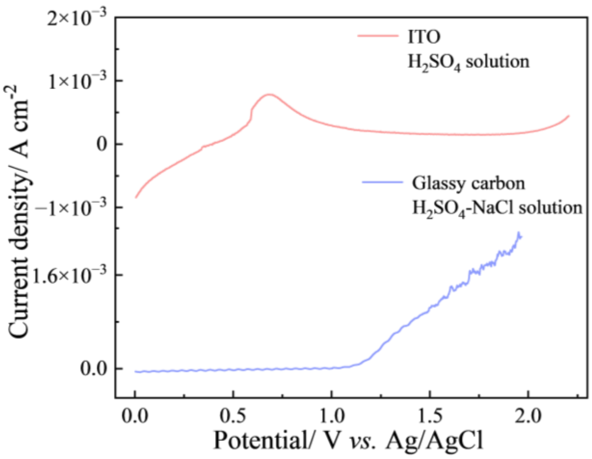


**Figure S1**. LSV curves obtained using s-ITO and glassy carbon as working electrodes in $H_2SO_4$ and $H_2SO_4$-NaCl solutions ($H_2SO_4$ concentration: 5.52 mol $L^{-1}$, NaCl concentration: 1.2 mol $L^{-1}$, scan rate: 0.1 V $s^{-1}$).

**Issue 7:**

In Fig. 2, conclusions are drawn primarily from XPS analysis. Given the highly surface-sensitive nature of XPS, it is difficult to draw representative conclusions about bulk composition or phase evolution. Depth-profile XPS or complementary bulk-sensitive characterization should be provided to support the interpretations.

**Discussion:**

We appreciate the reviewer for raising this critical methodological concern regarding the surface sensitivity of X-ray photoelectron spectroscopy (XPS) and its limitations in representing bulk composition and phase evolution. Exclusive reliance on XPS data of surface may introduce interpretation bias due to the technique's inherent surface-sensitive. Complementary bulk-sensitive characterization is essential for robust conclusions regarding material evolution during electrolysis.

For profile analysis of s-ITO after electrolysis: S-ITO was electrolyzed in $H_2SO_4$-NaCl solution (5.52 mol $L^{-1}$ $H_2SO_4$ and 1.2 mol $L^{-1}$ NaCl) at a current density of 0.116 A $cm^{-2}$ for 1.5 h. After electrolysis, the s-ITO was rinsed with deionized water. The surface of the reacted s-ITO was polished with 2000-mesh sandpaper until the unreacted internal region was exposed, yielding a profile with the reaction zone at the edge and the unreacted zone in the inner part. Subsequently, the profile was cleaned with deionized water and ethanol, followed by drying. XPS analysis was performed using a laser spot with a size of 200 μm, and the analyzed regions

were the reacted edge and the unreacted central region.

The depth-profiling XPS results after electrolysis are presented in Figure S4. The In 3d5/2 binding energies in the near-surface reaction region and the middle unreacted region are identical. Similarly, the Sn 3d5/2 binding energies show a similar variation trend. The absence of binding energy shift or peak asymmetry indicates that the oxidation states and local chemical environments of indium and tin remain unchanged in both electrolyzed and non-electrolyzed regions. This observation confirms that In and Sn do not undergo redox transitions during the electrochemical process. In sharp contrast, oxygen exhibits obvious regional heterogeneity. The near-surface region shows a significant reduction in the lattice oxygen component at 529.7 eV. This is consistent with the phenomenon observed on the s-ITO surface.

These micro-area XPS results provide direct spectroscopic evidence for the dissolution of the s-ITO electrode: the formation of oxygen vacancies precedes the leaching of indium and tin ions, rather than direct metal oxidation. This spatially resolved analysis verifies that surface-sensitive measurements truly capture the kinetics of electrochemical transitions, rather than artifacts from surface contamination or atmospheric exposure.

**The related revision in the main text:**

**Lines 578-585 in Methods Section：**

For profile analysis of s-ITO after electrolysis: S-ITO was electrolyzed in $H_2SO_4$-NaCl solution (5.52 mol $L^{-1}$ $H_2SO_4$ and 1.2 mol $L^{-1}$ NaCl) at a current density of 0.116 A $cm^{-2}$ for 1.5 h. After electrolysis, the s-ITO was rinsed with deionized water. The surface of the reacted s-ITO was polished with 2000-mesh sandpaper until the unreacted internal region was exposed, yielding a profile with the reaction zone at the edge and the unreacted zone in the inner part. Subsequently, the profile was cleaned with deionized water and ethanol, followed by drying. XPS analysis was performed using a laser spot with a size of 200 μm, and the analyzed regions were the reacted edge and the unreacted central region.

**Lines 156-162 in Results Section:**

To eliminate the potential errors arising from the surface sensitivity of XPS measurements, depth-profiling XPS measurements were further performed on the post-electrolysis samples, and the results are shown in **Figure S4**. A spot with a smaller size was adopted for the tests. Compared with the unreacted central region in the depth profile, the near-surface reaction

region exhibited significant changes, which were consistent with the results illustrated in **Figure 2a-c**. This further confirms the preferential interaction between charges and oxygen in s-ITO.

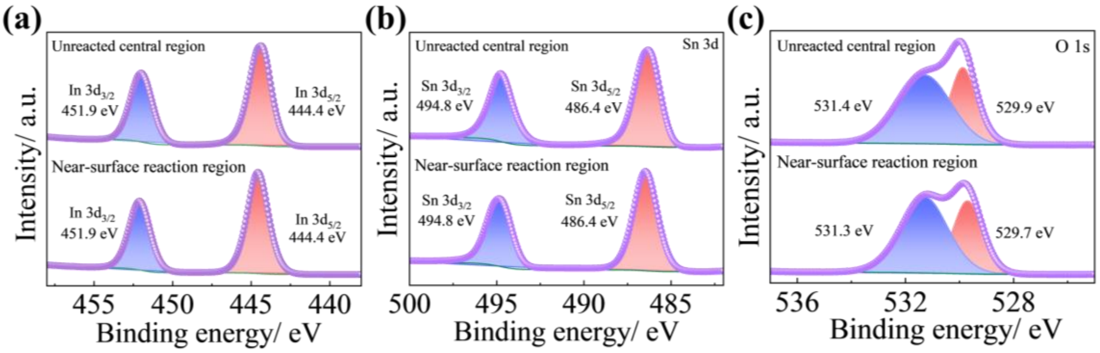


**Figure S4**. Microzone XPS spectra of s-ITO profile after electrolysis: (a) In 3d, (b) Sn 3d, (c) O 1s.

**Issue 8:**

In several instances, conclusions are presented with insufficient caution. For example, in Line 375, the statement that "this shift is primarily attributed to the shield effect" is made without clear experimental or theoretical evidence. Such claims should be either substantiated with data or revised to reflect a more tentative interpretation.

**Discussion:**

We appreciate the reviewer for carefully examining our statement regarding the shielding effect. We have removed this conceptual expression and replaced it with a more comprehensible and unambiguous description to illustrate the phenomena and mechanisms of the influence of Na ions on the cathodic reaction, all of which are based on authentic experimental or computational results.

**The related revision in the main text:**

**Lines 271-274 in Results Section:**

Likewise, a well-defined reduction peak corresponding to indium ions was observed on the CV curve. Accordingly, it can be deduced that the introduction of NaCl promoted the reduction of these ions at the cathode, with a more pronounced effect exerted on indium ions.

**Lines 288-307 in Results Section:**

After the addition of NaCl, the $H_2SO_4$-NaCl solution exhibited a more negative hydrogen

evolution potential (**Figure 4f**), indicating that the addition of NaCl effectively inhibited HER. As shown in **Figure 4g**, the adsorption energy of $H^+$ on the Ti cathode surface is -6.31 eV. The blue region indicates electron gain, and the red region indicates electron loss[51]. In the differential charge density diagram of the vertical section, an obvious red electron cloud appears around $H^+$, and an obvious blue electron cloud is generated at the adsorption site, indicating that electrons are transferred from $H^+$ to the electrode surface. In contrast, the adsorption energy of $Na^+$ is -6.49 eV (**Figure 4h**), indicating that $Na^+$ is more likely to be adsorbed on the Ti cathode than $H^+$. An obvious blue electron cloud appears around $Na^+$, and the adsorption site presents a red electron cloud, indicating that electrons on the electrode surface are transferred to $Na^+$. **Figure 4i** shows that the average differential charge density of $H^+$ in the Z direction is -0.012 e $Å^{-1}$, indicating that $H^+$ adsorption leads to electron enrichment on the surface[52]. The results in **Figure 4j** show that the average differential charge density of $Na^+$ is -0.075 e $Å^{-1}$, and $Na^+$ adsorption leads to more electron accumulation[53]. The electron distribution on the electrode surface has an obvious impact on the attraction and reduction process of metal ions. Compared with $H^+$ adsorption, $Na^+$ adsorption leads to an increase in the electron content on the electrode surface, and a significant change in the surface charge density, forming a localized electron-rich structure[54], which results in a stronger interaction between the Ti cathode and indium/tin ions[55,56], thereby promoting the adsorption and reduction processes of indium ions and tin ions on the cathode surface.


51. Geng, Z. *et al.* Charge density redistribution mechanism in dual-functional electrodes for enhanced the sulfophilic and lithiophilic of Li-S cells. *Chem. Eng. J.* **505**, 159333 (2025).
52. Guo, H., Pan, J. & Du, S. First-principles study of the schottky contact, tunneling probability, and optical properties of MX/$TiB_4$ heterojunctions (M = Ge, Sn; X = S, Se, Te): Strain engineering tunability. *ACS Appl. Mater. Interfaces* **16**, 31513–31523 (2024).
53. Gao, X., Zhang, L.-W. & Liew, K. M. Specific adsorption induced electric double layer on gallium-based liquid metal electrode. *Appl. Surf. Sci.* **636**, 157804 (2023).
54. Liu, J. *et al.* Reconstruction of electric double layer on the anode interface by localized electronic structure engineering for aqueous Zn ion batteries. *Adv. Energy Mater.* **14**, 2401118 (2024).
55. Jiang, L. *et al.* Electric double layer design for Zn-based batteries. *Energy Storage Mater.*

**62**, 102932 (2023).

56. Shi, X. *et al.* Compacting electric double layer enables carbon electrode with ultrahigh Zn ion storage capability. *Angew. Chem. Int. Ed.* **61**, e202214773 (2022).

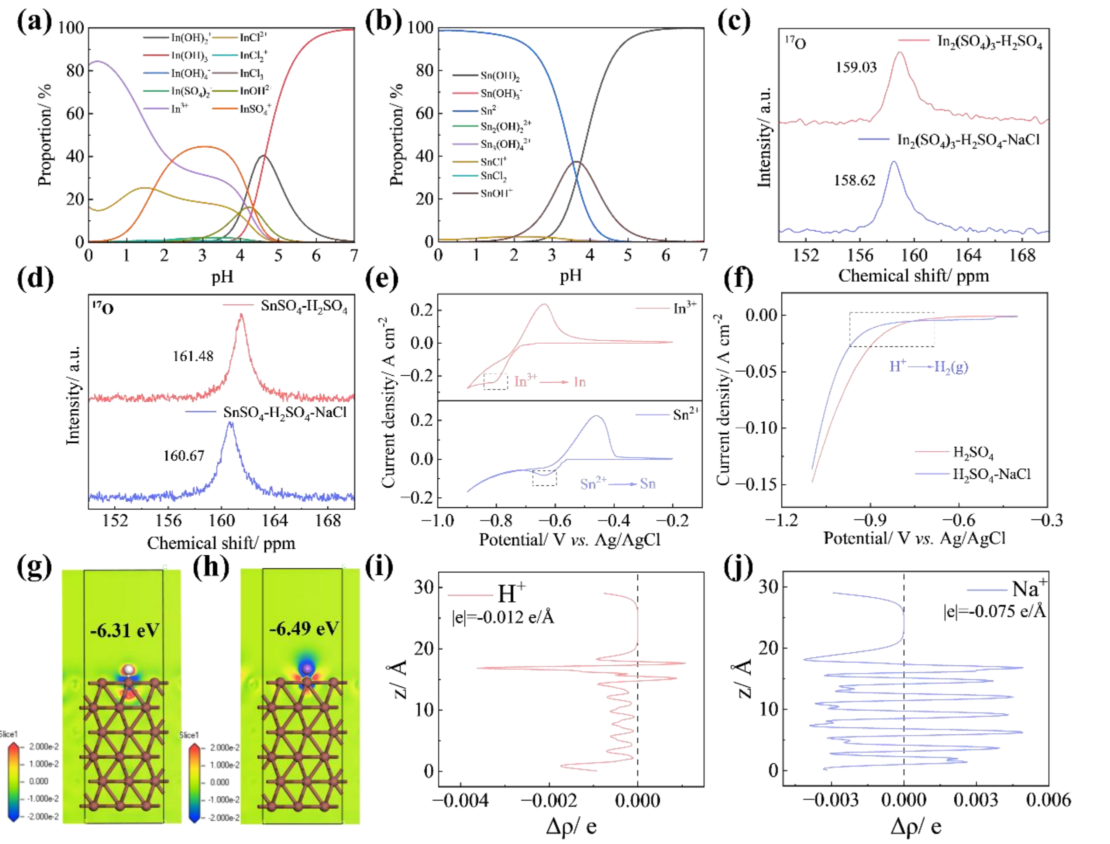


**Figure 4**. Research on the existential forms and electrochemical behaviors of In ions and Sn ions. The ion distribution maps of $In^{3+}$ (a) and $Sn^{2+}$ (b) in NaCl-$H_2SO_4$ solution; $^{17}O$ NMR spectra of $In_2(SO_4)_3$-$H_2SO_4$ (c) and $SnSO_4$-$H_2SO_4$ (d) solutions before and after adding NaCl; (e) CV curves of indium ions and tin ions ($H_2SO_4$ concentration: 5.52 mol $L^{-1}$, NaCl concentration: 1.2 mol $L^{-1}$, indium ions concentration: 0.01 mol $L^{-1}$, tin ions concentration: 0.01 mol $L^{-1}$, scan rate: 0.02 V $s^{-1}$); (f) LSV curves obtained in $H_2SO_4$ and $H_2SO_4$-NaCl solutions ($H_2SO_4$ concentration: 5.52 mol $L^{-1}$, NaCl concentration: 1.2 mol $L^{-1}$, scan rate: 0.1 V $s^{-1}$); Differential charge density sectional projection: (g) $H^+$ adsorption, (h) $Na^+$ adsorption; Distribution of cumulative differential charges in the z-axis direction: (i) $H^+$ adsorption, (j) $Na^+$ adsorption.

**Issue 9:**

In Line 386, the authors refer to "the square root of the peak current density," whereas the

classical electrochemical relationship involves the square root of the scan rate and the peak current itself. This statement appears to be incorrect and should be carefully revised.

**Discussion:**

We sincerely appreciate your valuable correction. The classic electrochemical relationship is between the square root of the scan rate and the peak current (or peak current density), rather than the square root of the peak current density. Although the fitting in our figure is correct, we inadvertently used an incorrect expression in the text due to oversight. We have now revised the text in accordance with your suggestion to ensure accuracy and rigor.

**The related revision in the main text:**

**Lines 278-282 in Results Section:**

Linear fitting analyses reveal a direct proportional relationship between the peak current density and the square root of the scan rate, while a linear correlation is also observed between the reduction peak potential and the logarithm of the scan rate. These findings collectively demonstrate that the reduction of indium ions is a diffusion-controlled quasi-reversible process.

**Issue 10:**

The experimental details provided for Section 3.4 are severely insufficient. While photographs of electrodes are included in the Supporting Information, essential information such as cell configuration, electrode dimensions, cell geometry, agitation or stirring conditions, and electrolyte flow rate is missing. In particular, the rationale for selecting a current density of 0.072 A $cm^{-2}$ is not provided. The authors should also clarify why potentiostatic operation was chosen instead of galvanostatic control and provide a sound theoretical justification for this choice.

**Discussion:**

We sincerely appreciate the reviewer's thorough and constructive comments regarding the experimental details presented in Section 3.4. This section has been comprehensively revised to address the raised concerns.

Furthermore, regarding the selection of current density and electrolysis mode. Galvanostatic electrolysis was adopted throughout this work, which represents a widely employed operational strategy in aqueous electrowinning and electrorefining processes. Especially in electrolyte

systems where the electrochemical discrepancies between target metal ions and impurity species are difficult to precisely quantify, galvanostatic polarization facilitates effective impurity elimination, thereby rationalizing our choice of electrolysis mode. With respect to the selected current density of 0.072 A $cm^{-2}$, such a magnitude falls within the conventional range applied in industrial practice. For instance, typical current densities for copper electrowinning and electrorefining generally lie between 0.01 and 0.1 A $cm^{-2}$. Notably, the present current density was primarily intended to verify the charge-induced acceleration of anodic dissolution. As illustrated in the parametric optimization results in **Figure 6**, this value does not correspond to the optimal condition; nevertheless, it is sufficiently appropriate to validate the proposed mechanism of charge-promoted anodic dissolution behavior.

The above discussion clarifies the fundamental rationale for adopting galvanostatic operation together with the specified current density in this study.

**The related revision in the main text:**

**Lines 535-558 in Methods Section:**

**Electrolysis recovery of s-ITO**

Exploration of electrolysis parameters

The anode employed here was s-ITO obtained after wire cutting, with dimensions of approximately 40 mm * 8 mm * 5 mm. The cathode was a high-purity titanium plate (99.99%) with dimensions of 100 mm * 10 mm * 2 mm. The electrolysis cell employed was a cylindrical glass reactor with a maximum working volume of 100 mL. The interelectrode distance between the anode and cathode was fixed at 2.0 cm, and no stirring was applied throughout the electrolysis process. A DC power supply (model UTP1310-II) was used, with a voltage control range of 0-64 V and a current control range of 0-16 A.

Effect of $H_2SO_4$ concentration: Electrolysis was carried out at 0.116 A $cm^{-2}$ for 1.5 h without initial $In^{3+}$ addition, at a fixed NaCl concentration of 1.2 mol $L^{-1}$. The concentrations of $H_2SO_4$ were 3.68 mol $L^{-1}$, 4.60 mol $L^{-1}$, 5.52 mol $L^{-1}$, and 6.44 mol $L^{-1}$, respectively.

Effect of NaCl concentration: Electrolysis was performed at 0.116 A $cm^{-2}$ for 1.5 h without initial $In^{3+}$ addition, at a fixed $H_2SO_4$ concentration of 5.52 mol $L^{-1}$. The concentrations of NaCl were 0.86 mol $L^{-1}$, 1.03 mol $L^{-1}$, 1.20 mol $L^{-1}$, 1.37 mol $L^{-1}$ and 1.53 mol $L^{-1}$, respectively.

Effect of current density: Electrolysis was conducted for 1.5 h without initial $In^{3+}$ addition, at fixed concentrations of 5.52 mol $L^{-1}$ $H_2SO_4$ and 1.2 mol $L^{-1}$ NaCl. The current densities were set to 0.070 A $cm^{-2}$, 0.093 A $cm^{-2}$, 0.116 A $cm^{-2}$, 0.140 A $cm^{-2}$ and 0.163 A $cm^{-2}$, respectively.

Effect of initial $In^{3+}$ concentration: Electrolysis was carried out for 1.5 h at 0.116 A $cm^{-2}$, at fixed concentrations of 5.52 mol $L^{-1}$ $H_2SO_4$ and 1.2 mol $L^{-1}$ NaCl, with different initial $In^{3+}$ concentrations added. The concentrations of $In^{3+}$ were 0.1 mol $L^{-1}$, 0.2 mol $L^{-1}$ and 0.3 mol $L^{-1}$, respectively.

After electrolysis, the cathodic and anodic current efficiencies were calculated. The detailed calculation method is provided in **Section 3** of **Supplementary Information**.

**Issue 11:**

According to Lines 460-470, indium-only recovery is achieved in the absence of NaCl, whereas In-Sn alloy formation occurs when NaCl is added (Fig. S10). However, this observation appears inconsistent with the CV data shown in Fig. S5 and Fig. 3e, where indium deposition is predicted to be suppressed without NaCl, while tin deposition should remain feasible. The discrepancy between electrochemical predictions and experimental outcomes must be carefully addressed.

**Discussion:**

We sincerely appreciate the reviewer for identifying the inconsistency between our electrochemical predictions and experimental results. Regarding this issue, we have conducted experiments and characterizations from the following aspects to explain it more clearly and accurately.

For electrolysis recovery in the $H_2SO_4$-NaCl solution, XRD characterization of the product confirms it to be an In-Sn alloy ($In_{0.959}Sn_{0.041}$), whereas the product recovered in $H_2SO_4$ was initially identified as metallic In via XRD. Due to the close similarity of diffraction peaks between In and $In_{0.959}Sn_{0.041}$ phases, we did not strictly distinguish them during the initial peak assignment, as the presence of Sn had already been verified by EDS and other characterization techniques. However, as noted by the reviewer, to ensure the rigor of characterization, we have re-conducted a careful comparison of the XRD diffraction peaks in both $H_2SO_4$-NaCl and $H_2SO_4$ solutions. The results clearly demonstrate that the products in both systems are

$In_{0.959}Sn_{0.041}$ (**Figure S14**).

During the electrochemical testing stage, in the $H_2SO_4$-NaCl solution, reduction peaks corresponding to Sn ions and In ions can be observed in the cyclic voltammetry (CV) curves, confirming that both Sn ions and In ions can be reduced in this system. In contrast, in the $H_2SO_4$ solution, the reduction peak of Sn ions is detectable, while the reduction peak of In ions is not observed initially. Nevertheless, when we supplemented the more sensitive square wave voltammetry (SWV) measurements, a significant reduction peak of In ions was clearly identified (**Figure S9**). The above results indicate that both In ions and Sn ions can be reduced in the $H_2SO_4$ solution, and the addition of NaCl can promote this reduction process.

In summary, it can be concluded that in the $H_2SO_4$ solution, both In ions and Sn ions can be reduced and deposited, yielding an In-Sn alloy at the cathode. Upon the addition of NaCl, the reductive deposition process is enhanced, and the cathode product remains an In-Sn alloy. The relevant analyses have been incorporated into the revised manuscript.

**The related revision in the main text:**

**Lines 264-274 in Results Section:**

In the $H_2SO_4$ solution containing tin ions, a prominent current signal was observed at -0.61 V (*vs*. Ag/AgCl) on the cyclic voltammetry (CV) curve (**Figure S9a**), which indicates the occurrence of the reduction reaction of tin ions. In contrast, no distinguishable reduction peak could be identified on the CV curve recorded in the $H_2SO_4$ solution with indium ions; however, a reduction signal emerged at -0.72 V (*vs*. Ag/AgCl) on the square wave voltammetry (SWV) curve with higher detection sensitivity (**Figure S9b**), suggesting that indium ion is reducible in the $H_2SO_4$ solution. Nevertheless, upon the addition of NaCl to the $H_2SO_4$ solution, the reduction signal of tin ions on the CV curve was significantly enhanced (**Figure 4e**). Likewise, a well-defined reduction peak corresponding to indium ions was observed on the CV curve. Accordingly, it can be deduced that the introduction of NaCl promoted the reduction of these ions at the cathode, with a more pronounced effect exerted on indium ions.

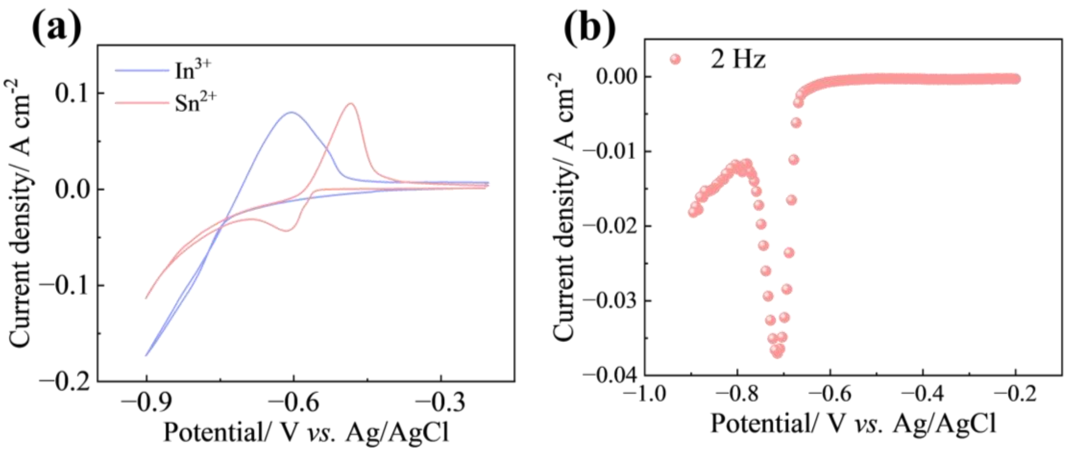


**Figure S9**. CV and SWV curves in 5.52 mol L$^{-1}$ $H_2SO_4$ solution. (a) CV curves of indium ion and tin ion (indium ion concentration: 0.01 mol L$^{-1}$, $Sn^{2+}$ concentration: 0.01 mol L$^{-1}$, scan rate: 0.1 V s$^{-1}$); (b) SWV curve of indium ion (indium ion concentration: 0.01 mol L$^{-1}$)

**Lines 335-339 in Results Section:**

After electrolysis in $H_2SO_4$ solution, a thin layer of silvery-white metal was deposited on the cathode, and XRD analysis confirmed that the product was In-Sn alloy (**Figure S14a**). With the addition of NaCl, the cathode product exhibited a porous and sponge-like morphology with a significantly increased mass. XRD results verified that the product was still In-Sn alloy (**Figure S14b**).

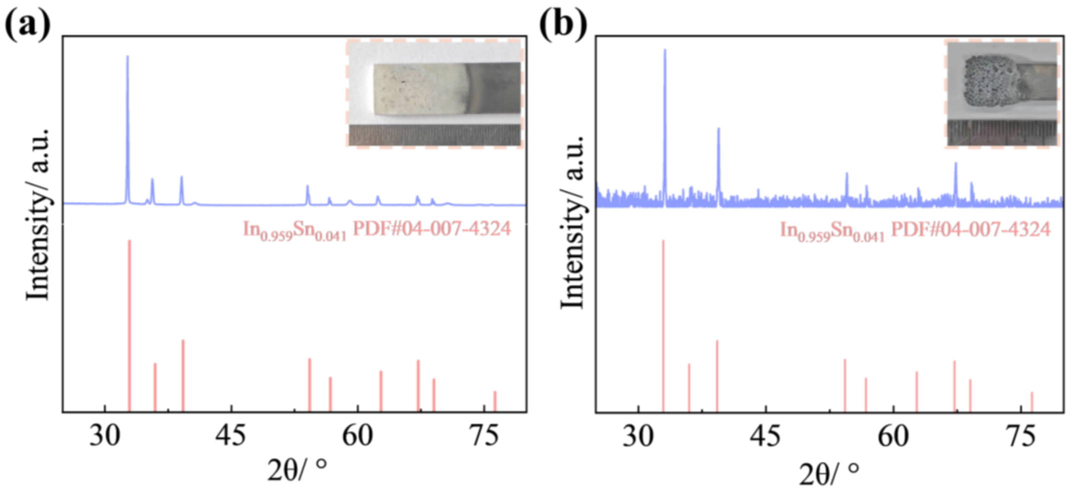


**Figure S14**. XRD pattern of cathodic products: (a) $H_2SO_4$ solution, (b) $H_2SO_4$-NaCl solution ($H_2SO_4$ concentration: 5.52 mol L$^{-1}$, NaCl concentration: 1.0 mol L$^{-1}$, current density: 0.072 A cm$^{-2}$, duration: 1.5 h).

**Issue 12:**

The Supporting Information also lacks sufficient detail. For example, in the calculation of

current efficiency, the number of electrons transferred ($z$) is mentioned but not explicitly defined. The actual values used in the calculations should be clearly stated to ensure transparency and reproducibility.

**Discussion:**

We appreciate the reviewer's valuable comment that our Supplementary information was insufficiently detailed. The reviewer is correct that transparency in quantitative calculations is critical for ensuring experimental reproducibility. We have comprehensively revised the Supplementary information to rectify this issue.

**The related revision in the Supplementary information:**

**Lines 46-72 in Section 3:**

**Section 3: Calculation of current efficiency**

Current efficiency is an important indicator for measuring the electrolysis effect, and it was calculated according to the following formula[4]:

$$m = \frac{It\mathrm{M}}{\mathrm{F}z} \tag{2}$$

$$\eta = \frac{m_l}{m} \tag{3}$$

In the formula, $m$ and $m_l$ are the theoretical and actual masses of the product obtained by electrolysis, g; $I$ represents the current, A; $t$ is the electrolysis time, s; M is the atomic mass; $z$ represents the number of moles of transferred electrons.

A calculation was performed using a certain electrolysis process as an example. The electrolysis time was 1.5 h, the current density was 0.116 A $cm^{-2}$ (with a current of 0.5 A), and the actual mass of the cathodic product (In-Sn alloy) was 0.5555 g. According to the XRD results, the atomic ratio of In to Sn in the obtained alloy was 0.959: 0.041. The relative atomic mass of In is 114.82, and that of Sn is 118.71; thus, the relative atomic mass of the In-Sn alloy was calculated as 114.82×0.959 + 118.71×0.041 = 114.98. The number of electrons transferred during the reduction of $In^{3+}$ to metal In is 3, and that during the reduction of $Sn^{4+}$ to metal Sn is 4; therefore, the total number of electrons transferred during the reduction to form the alloy was 3×0.959 + 4×0.041 = 3.041. Based on the above formula, the theoretical product mass was 1.058 g, and the current efficiency was 52.5%.

When the influence of a small amount of Sn was not considered and pure indium was assumed

to be obtained, the number of transferred electrons and the relative atomic mass were set as 3 and 114.82, respectively. The theoretical product mass was 1.071 g and the calculated current efficiency was 51.87%. The difference between this value and the result obtained using the In-Sn alloy was very small. In view of the low Sn content in the cathodic product, using In instead of the In-Sn alloy for current efficiency calculation had little impact on the final result and would not affect the trend of efficiency change. Therefore, to simplify the calculation of current efficiency, In was used instead of the In-Sn alloy. In the calculation, the value of $z$ was set to 3, and the relative atomic mass was 114.82.

**Issue 13:**

Although the manuscript focuses primarily on recovery purity, the behavior of the system under prolonged electrodeposition aimed at achieving high recovery yields is not discussed. How does extended operation affect selectivity, recovery efficiency, and solution pH? These aspects are essential for evaluating the practical viability of the process and should be addressed.

**Discussion:**

We sincerely appreciate the reviewers for their insightful comments regarding the practical feasibility of our proposed process. The reviewer's concern is highly pertinent. Prolonged operational stability is critical for evaluating the industrial applicability of an electrochemical process. We acknowledge that these key engineering parameters were not sufficiently addressed in the original manuscript. Accordingly, we have performed supplementary experiments and extensively revised the original text accordingly.

A total electrolysis duration of 15 h was implemented, with each cycle lasting 1.5 h. Sampling of both electrolyte and cathode products was conducted every two cycles. Systematic characterizations were performed, including determination of electrolyte acidity and impurity concentrations, analysis of impurity contents in products, and calculation of current efficiency. During electrolysis, a portion of the metal ions dissolved from the anode undergoes reduction and deposition at the cathode, while the residual metal species remain dissolved in the electrolyte. Upon completion of electrolysis, these residual ions can be fully recovered by replacing the anode with an inert electrode to induce further electrodeposition. Accordingly, calculating recovery efficiency merely based on cathode deposition mass and anode weight loss

is inappropriate. In principle, nearly zero raw material loss ensures a theoretically achievable recovery close to 100%. For the above reasons, we focused our evaluation on the variation of proton concentration, impurity accumulation in electrolyte and products, as well as cathode current efficiency, rather than recovery efficiency.

Over ten consecutive electrolysis cycles (five sampling points in total), the $H^+$ concentration in the electrolyte remained stable at approximately 90 mol $L^{-1}$. The concentrations of major impurity ions exhibited a cumulative increment, wherein the contents of Al, Mn, Fe, and Si gradually increased from initial levels of 0.2 ppm, 0.02 ppm, 0.7 ppm and 7.3 ppm to 1.4 ppm, 0.09 ppm, 1.9 ppm and 10.9 ppm, respectively. Correspondingly, the impurity contents in the harvested products showed minor variations yet remained at a consistently low level, with Al, Mn, Fe, and Si all below 17.9 ppm, 1.0 ppm, 18.3 ppm and 56.4 ppm. In addition, the current efficiency progressively increased and eventually stabilized at approximately 71% upon repeated cycling (**Figure S20**).

These results demonstrate that the electrolyte maintains stable acidity with slow and mild impurity accumulation throughout cyclic electrolysis, reflecting excellent operational stability of the proposed system. The above analyses have been incorporated into the revised manuscript.

**The related revision in the main text and Supplementary Information:**

**Lines 82-100 in Supplementary Information:**

**Section 5: Reusability experiment of the electrolyte**

To evaluate the reusability of the electrolyte, the same electrolysis process was continuously repeated in the original electrolyte after each electrolysis run, with a total of 10 consecutive runs conducted. The duration of each electrolysis is 1.5 h. The electrolyte was composed of 100 mL of 5.52 mol $L^{-1}$ $H_2SO_4$ and 1.2 mol $L^{-1}$ NaCl. For each electrolysis run, cathodes (titanium plates) and anodes (s-ITO) with the same dimensions were employed, and both the electrode spacing and current density (0.116 A $cm^{-2}$) were maintained constant. Impurity analysis was conducted on the products and electrolyte collected subsequent to the second run, accompanied by quantitative determination of the hydrogen ion concentration within the electrolyte. Subsequent sampling and characterization were implemented at intervals of every other cycle throughout the entire electrolysis process.

The determination of hydrogen ion concentration was achieved by measuring the pH value

of the diluted solution: 100 μL of the electrolyte was pipetted and diluted to 10 mL with deionized water, followed by pH measurement using a PHS-25 benchtop pH meter, and finally the hydrogen ion concentration in the solution was calculated according to Formula 5. The calculated $H^+$ concentration was then multiplied by 100 to obtain the $H^+$ concentration in the original electrolyte.

$$pH=-\lg C \tag{5}$$

where $C$ is the hydrogen ion concentration (mol $L^{-1}$).

**Lines 462-474 in Results Section:**

In contrast, the electrolyte employed in this technology exhibits excellent reusability. After 10 cycles of repeated electrolysis (each cycle duration: 1.5 h, current density: 0.116 A $cm^{-2}$), the $H^+$ concentration in the electrolyte remained nearly constant (**Figure S20a**; the measurement method of $H^+$ concentration is provided in **Section 5** of **Supplementary Information**), whereas the concentrations of the main impurities increased gradually (**Figure S20b**). This observation indirectly demonstrates the impurity purification effect of the proposed process, in which most impurities are retained in the electrolyte during electrolysis. Accordingly, the contents of main impurities in the final cathodic product were still markedly reduced (**Figure S20c**), similar to those of the product obtained from a single electrolysis run. With an increasing number of cycles, the electrolysis process gradually stabilized, and the current efficiency in the later stage remained steady at approximately 71% (**Figure S20d**). These results clearly confirm that the proposed technology enables the repeated reuse of the electrolyte, thereby significantly reducing wastewater generation.

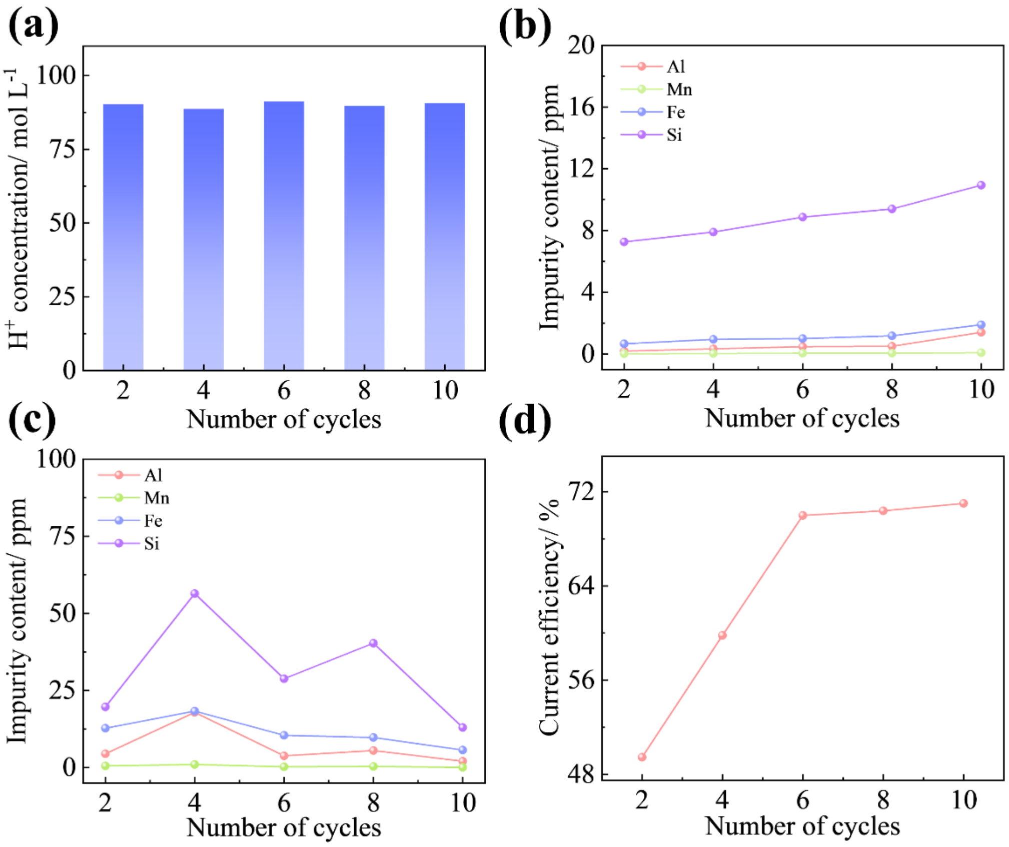


**Figure S20**. Evaluation of electrolyte stability under multi-cycle electrolysis operation (the duration of each electrolysis is 1.5 h and current density is 0.116 A $cm^{-2}$; the electrolyte is composed of 100 mL of 5.52 mol $L^{-1}$ $H_2SO_4$ and 1.2 mol $L^{-1}$ NaCl.). (a) Changes of $H^+$ concentration of electrolyte; The changes of the main impurity content after 10 cycles of electrolysis: (b) electrolyte, (c) cathode product; (d) Cathode current efficiency.

**Issue 14:**

In Fig. S17, reusability is assessed mainly from the perspective of impurity accumulation. However, a more holistic evaluation is required, including recovery efficiency, recovery purity, and current efficiency over multiple cycles. Without such data, the claim of process reusability remains insufficiently supported.

**Discussion:**

We appreciate the comment from the reviewer. This issue is analogous to **Issue 13**. We performed ten successive cyclic experiments under constant electrolyte conditions. The recyclability of the electrolyte was comprehensively evaluated by systematically examining the $H^+$ concentration evolution, impurity accumulation in the electrolyte, product purity, and

cathode current efficiency. Detailed experimental descriptions and corresponding result analyses have been presented in the discussion of **Issue 13**. The obtained results unambiguously demonstrate the excellent reusability and operational stability of the as-investigated electrolyte system.

**Issue 15:**

The authors compare the reported energy consumption primarily with pyro- and hydrometallurgical processes, which does not reflect the current state of the art. A more meaningful comparison would be with acid-leaching-based hydrometallurgical routes. For example, the statement "Through systematic optimization of electrolysis parameters, this method achieved a cathode current efficiency of 71.12% and an energy consumption of 4.87 kWh $kg^{-1}$ in hundred-ampere-scale trials" lacks proper context without benchmarking against comparable electrochemical or hydrometallurgical processes.

**Discussion:**

We appreciate this insightful comment and agree with the reviewer's suggestion.

In accordance with the recommendation, we have supplemented a more targeted and state-of-the-art benchmark comparison in the revised manuscript. Instead of only comparing with conventional pyrometallurgical and general hydrometallurgical routes, we have now systematically contrasted our Technology with representative acid-leaching-based hydrometallurgical processes.

All supplementary benchmark analyses have been incorporated into the revised manuscript.

**The related revision in the main text:**

**Lines 443-463 in Results Section:**

To better demonstrate the advantages of the present process, a comprehensive comparison and evaluation with the conventional acid leaching process was conducted from multiple dimensions, including energy consumption, process flow, temperature, product purity, and wastewater yield (**Figure 7e**). According to calculations reported in the literature, the energy consumption for recycling 1 kg of s-ITO via acid leaching-extraction processes ranges from 13.0 to 16.5 kWh[61]. In contrast, the proposed process exhibits much lower energy consumption. Traditional acid leaching suffers from complicated operation procedures, including raw

material crushing, stirring or ultrasonic treatment to accelerate leaching, solution fractionation, solvent extraction, and metal displacement reduction[62–64]. The cumbersome operations and lengthy procedures greatly restrict the efficient development of acid leaching technologies. In contrast, the proposed process only involves electrolysis as the core step, and its simple operation and single procedure endow it with great prospects for industrial application. With respect to reaction temperature, in contrast to the 75-90 °C required for acid leaching[93], the present process can be performed at room temperature, which not only reduces energy consumption but also lowers the requirements for the operating environment. Acid leaching usually requires post-treatment to obtain products with a purity higher than 99%[66]. The product obtained by the present process (after removal of main impurities) reaches a purity as high as 99.99%. In terms of wastewater, processes such as acid leaching and extraction in hydrometallurgical recovery generate large amounts of wastewater containing acids and organic solvents. In contrast, the electrolyte employed in this technology exhibits excellent reusability.

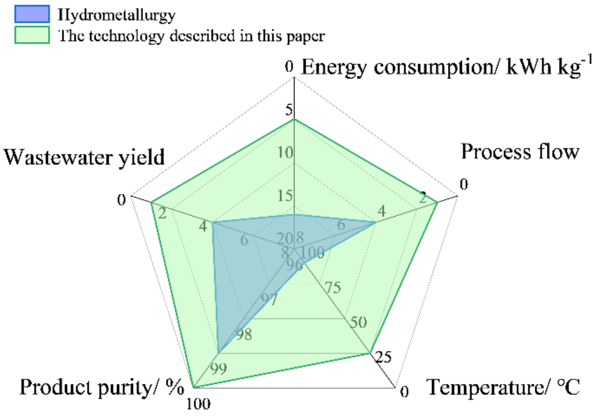


**Figure 7e**

61. Gu, S., Fu, B., Dobbie, G., Fujita, T. & Fang, B. A sustainable approach to separate and recover indium and tin from spent indium–tin oxide targets. *RSC Adv.* **7**, 52017–52023 (2017).

62. Swain, B., Mishra, C., Hong, H. S. & Cho, S.-S. Beneficiation and recovery of indium from liquid-crystal-display glass by hydrometallurgy. *Waste Manag.* **57**, 207–214 (2016).

63. Zhang, K. *et al.* Recycling of indium from waste LCD: A promising non-crushing leaching with the aid of ultrasonic wave. *Waste Manag.* **64**, 236–243 (2017).

64. Lokanc, M., Eggert, R. & Redlinger, M. The availability of indium: The present, medium

term, and long term. *NREL/SR*--**6A20-62409**, 1327212 (2015).

65. Li, Y., Liu, Z., Li, Q., Liu, Z. & Zeng, L. Recovery of indium from used indium–tin oxide (ITO) targets. *Hydrometallurgy* **105**, 207–212 (2011).

66. Hsieh, S.-J., Chen, C.-C. & Say, W. C. Process for recovery of indium from ITO scraps and metallurgic microstructures. *Mater. Sci. Eng. B* **158**, 82–87 (2009).

**Issue 16:**

During s-ITO leaching, experiments were conducted using 5.52 M $H_2SO_4$ and 1–1.2 M NaCl (approximately a 5:1 ratio, Fig. 5). However, the analyses presented in Fig. 3 were performed under different electrolyte compositions, where the $H_2SO_4$-to-NaCl ratio is 1:1 and overall concentrations also differ. It is therefore unclear whether the conclusions drawn from Fig. 3 are directly applicable to the actual s-ITO leaching conditions. Clarification is required as to whether similar trends would be observed under electrolyte compositions identical to those used in the leaching experiments.

**Discussion:**

We appreciate the reviewer's insightful comments. The inconsistency between the electrolyte composition used in electrochemical tests and other related experiments and that employed in actual electrolysis could indeed raise concerns about the applicability of the conclusions obtained from the tests to practical electrolysis processes. To verify the applicability of the technology and ensure the manuscript is more logically reasonable, we unified the electrolyte composition and re-conducted the relevant experiments presented in **Figure 3**. The results of the repeated experiments are consistent with those of the previous experiments, leading to the same conclusions.

**The related revision in the main text:**

**Lines 251-263 in Results Section:**

In the $H_2SO_4$ solution, as the pH decreases, $In^{3+}$ becomes the main existing form, and the content of $InSO_4^+$ complex ion decreases (**Figure S8a**). Similarly, in the low-pH environment, $Sn^{2+}$ tends to exist independently without complexing with $SO_4^{2-}$ (**Figure S8b**). However, with the addition of NaCl, the content of $InCl^{2+}$ increases significantly in the low-pH environment, indicating that $In^{3+}$ has a stronger affinity for $Cl^-$ (**Figure 4a**). The generation of $SnCl^+$ indicates

that the binding tendency of $Sn^{2+}$ with $Cl^-$ is much greater than that with $SO_4^{2-}$ (**Figure 4b**). The results demonstrate that $Cl^-$ exhibits a strong binding affinity toward $In^{3+}$ and $Sn^{2+}$, leading to the formation of chloro-coordinated complex ions. The Nuclear Magnetic Resonance (NMR) spectroscopic results reveal that the addition of NaCl to $H_2SO_4$ solution leads to a pronounced negative shift of the $^{17}O$ signal, which is ascribed to the decreased electron density around the oxygen atoms, indicating that NaCl weakens the interaction between $SO_4^{2-}$ and $In^{3+}$ [49] (**Figure 4c**). Notably, the introduction of $Cl^-$ also gives rise to a reduction in the chemical shifts of $Sn^{2+}$ and $SO_4^{2-}$ (**Figure 4d**), which is attributed to the strong binding affinity of $Cl^-$ with $Sn^{2+}$ [50].

49. Liang, P. *et al.* Competitive coordination of ternary anions enabling fast Li-ion desolvation for low-temperature lithium metal batteries. *Adv. Funct. Mater.* **34**, 2309858 (2024).

50. Liu, Y. Anion binding in solution: Beyond the electrostatic regime. *Chem.* **3**, 411-427 (2017).

**Lines 288-289 in Results Section:**

After the addition of NaCl, the $H_2SO_4$-NaCl solution exhibited a more negative hydrogen evolution potential (**Figure 4f**), indicating that the addition of NaCl effectively inhibited HER.

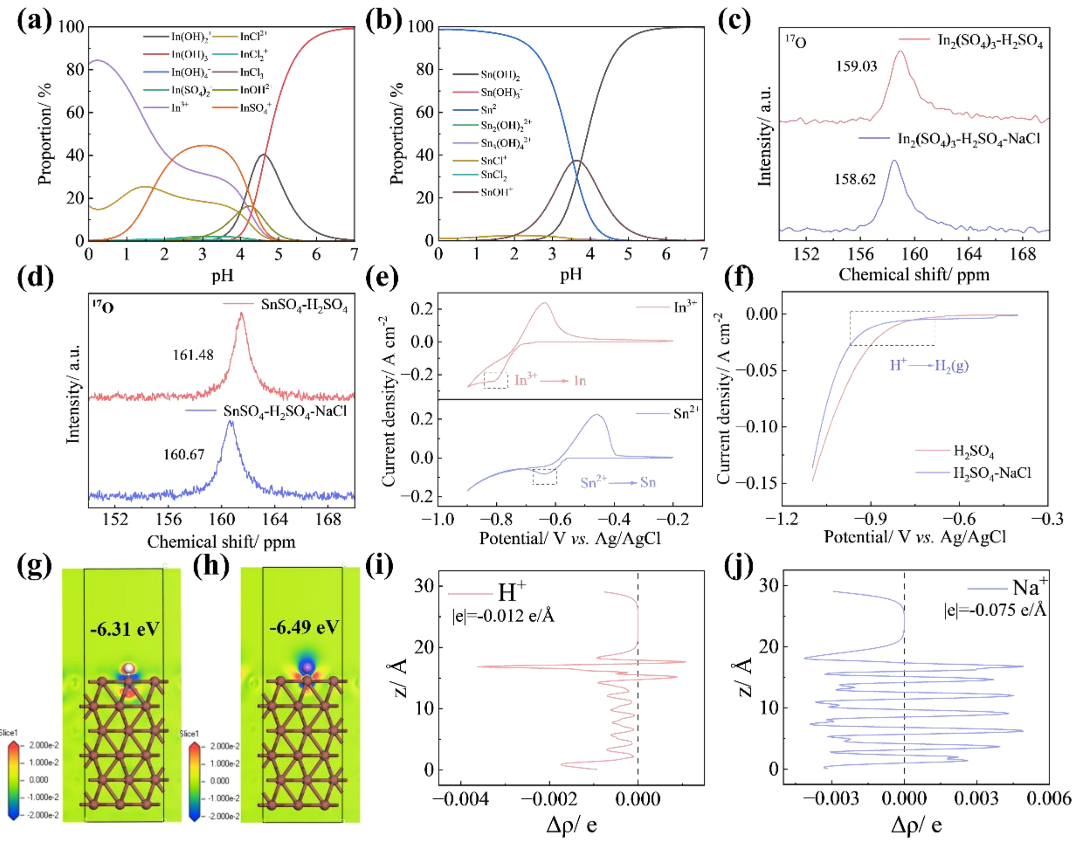


**Figure 4**. Research on the existential forms and electrochemical behaviors of In ions and Sn

ions. The ion distribution maps of $In^{3+}$ (a) and $Sn^{2+}$ (b) in NaCl-$H_2SO_4$ solution; $^{17}O$ NMR spectra of $In_2(SO_4)_3$-$H_2SO_4$ (c) and $SnSO_4$-$H_2SO_4$ (d) solutions before and after adding NaCl; (e) CV curves of indium ions and tin ions ($H_2SO_4$ concentration: 5.52 mol $L^{-1}$, NaCl concentration: 1.2 mol $L^{-1}$, indium ions concentration: 0.01 mol $L^{-1}$, tin ions concentration: 0.01 mol $L^{-1}$, scan rate: 0.02 V $s^{-1}$); (f) LSV curves obtained in $H_2SO_4$ and $H_2SO_4$-NaCl solutions ($H_2SO_4$ concentration: 5.52 mol $L^{-1}$, NaCl concentration: 1.2 mol $L^{-1}$, scan rate: 0.1 V $s^{-1}$); Differential charge density sectional projection: (g) $H^+$ adsorption, (h) $Na^+$ adsorption; Distribution of cumulative differential charges in the z-axis direction: (i) $H^+$ adsorption, (j) $Na^+$ adsorption.

**Issue 17:**

Related to the previous point, the manuscript should explicitly demonstrate whether the mechanistic interpretations derived from simplified electrolyte systems remain valid under the highly concentrated acid and chloride conditions employed for practical s-ITO leaching. Without such validation, extrapolation of the conclusions to realistic operating conditions remains questionable.

**Discussion:**

We appreciate the reviewer's valuable comment. The electrolyte composition used in the experiments shown in **Figure 1** was 5.52 mol $L^{-1}$ $H_2SO_4$ + 1.0 mol $L^{-1}$ NaCl, whereas the practical electrolysis adopted 5.52 mol $L^{-1}$ $H_2SO_4$ + 1.2 mol $L^{-1}$ NaCl. The reviewer questions whether the proposed mechanism remains valid despite such a variation in electrolyte formulation.

Undoubtedly, in a $H_2SO_4$ solution, the application of charge promotes the anodic dissolution of s-ITO (**Figure 1**). With the addition of NaCl, the corrosion potential of s-ITO shifts negatively, further accelerating the kinetic behavior of anodic dissolution (**Figures 1 and S3**). The experimental results in **Figure 1** validate the effectiveness of anodic dissolution, whereas the underlying mechanism is elucidated based on the data presented in **Figures 2 and 3**. Notably, the experimental conditions adopted in **Figures 2 and 3** correspond to the optimized parameters, which are identical to those used for scaled-up electrolysis. This confirms the applicability of the proposed mechanism in scale-up operations. Specifically, the inherent property that charge

accelerates the dissolution of s-ITO remains unchanged, while variations in electrolyte composition exert a regulatory effect on its kinetic processes. The newly supplemented explanations regarding the reaction mechanism have been incorporated into the revised manuscript.

**The related revision in the main text:**

**Lines 222-242 in Results Section:**

The electron localization function (ELF) of the oxygen vacancy formed by removing $O_{50}$ is shown in **Figure 3f**. A blue-green region appears at the center of the oxygen vacancy, which is caused by the two electrons released after the removal of oxygen being shared by the adjacent indium and tin ions. The electron localization density of the adjacent indium and tin ions increases, especially that of the adjacent tin ions, which increases significantly. The electrons generated by the oxygen vacancy are mainly shared by the adjacent tin ions, reflecting an increase in carriers. The ELF of the ITO (2 2 2) crystal plane with indium vacancies is shown in **Figure 3g**. A blue-green region appears at the center of the indium vacancy; since the three electrons generated by the indium vacancy are shared by the adjacent oxygen ions, the electron clouds of the adjacent oxygen ions are strongly attracted, leading to a significant expansion of the electron localization density of the adjacent oxygen ions. As shown in **Figure 3h**, the orange-yellow regions around the oxygen ions adjacent to the tin vacancy are significantly expanded, indicating a significant enhancement of the electron localization density of the oxygen ions. This is caused by the fact that the effective charge of tin ions is higher than that of indium ions, resulting in a stronger polarization effect on the electron cloud of oxygen ions. In summary, oxygen is the easiest to dissolve and form oxygen vacancies. The ionic radius of oxygen (1.71 Å) is significantly smaller than that of indium (2.46 Å) and tin (2.48 Å) [48]; therefore, the lattice distortion caused by the formation of oxygen vacancies is smaller, and the surrounding lattice relaxation energy is larger. Meanwhile, the charge difference of 2 electrons introduced after oxygen dissolution is significantly smaller than that caused by indium and tin dissolution, requiring less compensation energy; thus, oxygen vacancies are easier to form.

48. Rahm, M., Hoffmann, R. & Ashcroft, N. W. Corrigendum: Atomic and ionic radii of elements 1–96. *Chem. – Eur. J.* **23**, 4017–4017 (2017)
